# Hyperforin as a Multifunctional Specialized Metabolite of Natural Origin for Chronic Diseases: From Molecular Mechanisms to Clinical Translation

**DOI:** 10.3390/cimb48090937

**Published:** 2026-09-14

**Authors:** Efthymios Poulios, Sousana K. Papadopoulou, Exakousti-Petroula Angelakou, Gerasimos Tsourouflis, Aspasia Serdari, Constantinos Giaginis

**Affiliations:** 1 Department of Food Science and Nutrition, University of the Aegean, 81400 Lemnos, Greece; epoulios@aegean.gr (E.P.); xeniaggelakou@hotmail.com (E.-P.A.); 2 Department of Nutritional Sciences and Dietetics, International Hellenic University, 57001 Thessaloniki, Greece; souzpapa@gmail.com; 3 2nd Propaedeutic Department of Surgery, Laiko General Hospital of Athens, National and Kapodistrian University of Athens, 11527 Athens, Greece; gtsourouflis@med.uoa.gr; 4 Department of Psychiatry, School of Medicine, Democritus University of Thrace, 68100 Alexandroupolis, Greece; aserntar@med.duth.gr

**Keywords:** natural products, phytochemicals, therapeutic potential, translational medicine, clinical applications, bioavailability, pharmacokinetics, depression P-glycoprotein

## Abstract

Hyperforin, a polyprenylated acylphloroglucinol and major bioactive constituent of *Hypericum perforatum* L. (*H. perforatum*, St. John’s Wort), has attracted considerable interest because of its pleiotropic pharmacological properties. Although historically investigated primarily for its contribution to the antidepressant activity of *H. perforatum*, experimental studies have identified broader neuroprotective, anti-inflammatory, antioxidant, immunomodulatory, antimicrobial, metabolic, anticancer, and tissue-regenerative actions. However, the evidence remains heterogeneous, and an important distinction exists between findings obtained with purified hyperforin and those derived from multicomponent *H. perforatum* preparations. Unlike previous reviews that have primarily summarized the pharmacological activities of hyperforin, the present review adopts a translational evidence framework that distinguishes purified hyperforin from multicomponent *H. perforatum* preparations and evaluates the maturity of evidence from molecular mechanisms and preclinical models through pharmacokinetics, human studies, safety, and clinical development. A comprehensive narrative review was conducted according to the Scale for the Assessment of Narrative Review Articles (SANRA) recommendations. PubMed/MEDLINE, Scopus, Web of Science, Embase, and Google Scholar were searched from database inception through 25 July 2026. Mechanistic, in vitro, animal, translational, pharmacokinetic, and clinical studies, together with relevant systematic reviews and meta-analyses, were critically synthesized according to intervention type, disease-specific evidence, pharmacokinetic and formulation characteristics, safety, and translational relevance. Hyperforin acts as a pleiotropic signaling modulator affecting interconnected pathways involved in neurotransmission and neuroplasticity, inflammatory and redox regulation, mitochondrial and cellular homeostasis, immune function, and metabolism. Extensive cellular and animal evidence supports biological activity across neuropsychiatric, neurodegenerative, inflammatory, immune-mediated, cardiometabolic, oncological, gastrointestinal, infectious, dermatological, and other chronic disease settings. Nevertheless, evidence maturity differs markedly among indications. The strongest human evidence concerns standardized hyperforin-containing *H. perforatum* preparations for mild-to-moderate depression, whereas most other applications remain preliminary or predominantly preclinical. Adequately powered randomized controlled trials of purified hyperforin are lacking, preventing attribution of the clinical effects of *H. perforatum* preparations specifically to hyperforin. Translation is further constrained by poor aqueous solubility, chemical instability, variable bioavailability and formulation composition, limited compound-specific pharmacokinetic data, and clinically important herb–drug interactions, particularly through PXR-mediated induction of CYP3A4 and P-glycoprotein. Hyperforin is a pharmacologically promising multifunctional natural compound, but its experimental activity currently exceeds the strength of compound-specific clinical evidence. Future research should prioritize chemically defined and preferably purified formulations, rigorous pharmacokinetic and dose-finding studies, biomarker-supported target-engagement assessment, and adequately powered randomized controlled trials, together with long-term safety, interaction, standardization, and regulatory evaluation. Demonstrating predictable human exposure, biologically relevant target engagement, clinically meaningful efficacy, and an acceptable long-term safety profile will be essential for determining whether hyperforin can progress from experimental promise to evidence-based therapeutic application.

## 1. Introduction

### 1.1. Hyperforin as a Bioactive Natural Compound

Natural products remain important sources of pharmacologically active compounds for drug discovery and therapeutic development. Among plant-derived bioactive compounds, hyperforin, a polycyclic polyprenylated acylphloroglucinol from *Hypericum perforatum* L. (*H. perforatum*, St. John’s Wort), has attracted substantial interest because of its pleiotropic biological activities and potential therapeutic relevance across multiple pathological conditions [1,2].

The medicinal use of *H. perforatum* extends over two millennia, with historical applications for wounds, burns, inflammatory disorders, and neuropsychiatric conditions [1,2]. Although early pharmacological investigations focused largely on hypericin and related naphthodianthrones, subsequent studies identified hyperforin as an important contributor to several biological activities of *H. perforatum*, particularly those associated with its antidepressant effects [1,2].

*H. perforatum*, a perennial species of the *Hypericaceae* family, contains a chemically diverse phytocomplex comprising naphthodianthrones, flavonoids and other phenolic compounds, and phloroglucinol derivatives [3,4]. Hyperforin is considered one of its principal bioactive constituents because it modulates multiple molecular and cellular processes involving neurotransmission, inflammatory and redox signaling, cellular proliferation, apoptosis, and immune regulation [3,4].

More recently, advances in molecular pharmacology and translational research have broadened the investigation of hyperforin beyond mood disorders [4,5]. Experimental and preclinical studies using purified hyperforin and hyperforin-containing *H. perforatum* preparations have reported neuroprotective, anti-inflammatory, antioxidant, antimicrobial, anticancer, metabolic, and tissue-regenerative activities [4,5]. These findings have supported increasing interest in hyperforin as a multifunctional natural compound with potential relevance to a broad range of chronic and complex diseases.

### 1.2. Chemical Characteristics

Hyperforin belongs to the family of polyprenylated acylphloroglucinols (PPAPs), structurally distinctive secondary metabolites characterized by a phloroglucinol-derived core substituted with multiple lipophilic prenyl moieties [6,7]. It has the molecular formula C_35_H_52_O_4_ and a complex polycyclic architecture enriched with isoprenoid side chains that substantially influence its physicochemical properties, membrane interactions, and pharmacological behavior [6,7].

The multiple prenyl substituents confer pronounced lipophilicity, facilitating membrane partitioning, intracellular distribution, interactions with membrane-associated targets, and access to the central nervous system [8,9]. However, this high lipophilicity is accompanied by very low aqueous solubility and variable oral exposure, creating important limitations for reproducible pharmaceutical delivery [8,9].

Hyperforin is also chemically labile and susceptible to degradation induced by light, heat, oxygen, and oxidative conditions, which may affect the stability, composition, and reproducibility of hyperforin-containing preparations [4,8,10]. Thus, although its structural and physicochemical characteristics contribute to its membrane-associated pharmacological activity, poor aqueous solubility and chemical instability remain important barriers to reliable systemic exposure and pharmaceutical development [8,10,11]. These pharmacokinetic, bioavailability, and formulation-related limitations are discussed in detail in Section 3.

### 1.3. Rationale, Objectives, and Scope of the Review

Growing interest in plant-derived bioactive compounds as potential therapeutic agents has been reinforced by concerns regarding adverse effects associated with some conventional pharmacotherapies and by increasing attention to integrative healthcare approaches [12,13]. Within this context, hyperforin has emerged as an extensively investigated constituent of *H. perforatum*. Although initially studied primarily for its contribution to the antidepressant activity of St. John’s Wort preparations, subsequent research has revealed a broader pharmacological profile involving interconnected mechanisms related to neurotransmission and neuroplasticity, inflammatory and redox regulation, mitochondrial and cellular homeostasis, immune function, and metabolism [5,14]. These pleiotropic actions have prompted investigation of hyperforin across a broad range of chronic and complex diseases.

Despite the expanding literature, the evidence remains fragmented across mechanistic, preclinical, and clinical research. Moreover, major translational issues—including pharmacokinetic and bioavailability limitations, formulation and product standardization, safety and drug-interaction liability, and particularly the distinction between purified hyperforin and hyperforin-containing *H. perforatum* preparations—have not been consistently integrated into assessments of its therapeutic potential. A critical synthesis is therefore required to determine not only where hyperforin shows biological promise, but also how closely that promise has progressed toward clinically relevant evidence.

Previous reviews have provided valuable summaries of the chemistry and broad pharmacological activities of hyperforin, including its antidepressant, anticancer, neuroprotective, metabolic, and other biological effects, as well as aspects of extraction, synthesis, pharmacokinetics, structural modification, and pharmaceutical development [5,6,8,9,10,12,13]. However, the present review differs in both scope and analytical framework. Rather than primarily cataloguing reported pharmacological activities, it evaluates hyperforin along the complete translational continuum from molecular mechanisms and preclinical efficacy to human evidence, pharmacokinetic feasibility, formulation, safety, drug interactions, standardization, and regulatory considerations. Particular emphasis is placed on distinguishing purified hyperforin from multicomponent *H. perforatum* preparations, grading the relative maturity of evidence across disease-specific applications, and identifying the preclinical–clinical and compound–preparation attribution gaps that currently limit therapeutic translation. In addition, this review incorporates the literature published through 25 July 2026 and critically defines the experimental, pharmacokinetic, clinical, and regulatory requirements necessary for progression of hyperforin from a multifunctional natural compound to a chemically defined and evidence-based therapeutic agent.

The distinctive contribution of this review is its translational evidence framework. Accordingly, its principal added value is not simply an updated compilation of hyperforin pharmacology, but a critical assessment of whether the available evidence supports translation toward clinically applicable, hyperforin-specific interventions. To this end, we integrate molecular mechanisms, disease-specific efficacy, pharmacokinetics, formulation, safety, and human evidence while explicitly distinguishing purified hyperforin from hyperforin-containing *H. perforatum* preparations. We further compare the maturity of evidence across therapeutic domains and identify the principal experimental, pharmaceutical, clinical, and regulatory barriers that must be addressed before hyperforin can progress toward standardized, evidence-based therapeutic development.

## 2. Methods

### 2.1. Review Design

This study was conducted as a comprehensive narrative review aimed at critically evaluating and integrating current evidence on the biological activities, molecular mechanisms, therapeutic potential, and clinical translation of hyperforin. The review followed the recommendations of the Scale for the Assessment of Narrative Review Articles (SANRA), with emphasis on transparent literature searching, predefined eligibility criteria, and structured critical synthesis [15,16,17].

Because the review was designed as a broad, conceptually integrative narrative synthesis rather than a systematic review or meta-analysis, PRISMA guidelines were not applied, and procedures specific to systematic reviews—including independent duplicate screening and data extraction, formal risk-of-bias assessment, certainty-of-evidence grading, and quantitative evidence synthesis—were not performed [15,16,17]. Instead, mechanistic, in vitro, animal, translational, and human clinical studies, together with relevant systematic reviews and meta-analyses, were integrated qualitatively to evaluate the consistency, biological plausibility, clinical relevance, and translational maturity of the available evidence.

Particular attention was given to identifying knowledge gaps and methodological limitations and to distinguishing evidence obtained with purified hyperforin from that derived from hyperforin-containing *H. perforatum* preparations. Where hyperforin-specific evidence was limited, studies investigating *H. perforatum* preparations were considered when relevant, with the intervention type explicitly taken into account during interpretation. Principles of reproducible narrative evidence synthesis, including explicit documentation of the search strategy, predefined eligibility criteria, and structured qualitative synthesis, were applied throughout the review [15,16,17].

### 2.2. Literature Search Strategy

A comprehensive literature search was conducted in PubMed/MEDLINE, Scopus, Web of Science, Embase, and Google Scholar to identify experimental and clinical publications addressing the biological, pharmacological, and therapeutic properties of hyperforin. The search strategy combined controlled vocabulary, database-specific indexing terms, and free-text keywords. Core search terms included “hyperforin,” “hyperforin and health,” and “hyperforin and disease,” supplemented by combinations addressing specific mechanisms and applications, including “hyperforin and depression,” “hyperforin and inflammation,” “hyperforin and neuroprotection,” “hyperforin and cancer,” “hyperforin and antimicrobial,” “hyperforin and antioxidant,” “hyperforin and metabolism,” “hyperforin and cardiovascular,” and “hyperforin and clinical trial.” Additional searches incorporated the terms “*Hypericum perforatum*,” “St. John’s Wort,” “TRPC6 activation,” “mechanisms of action,” “pharmacology,” “bioavailability,” “drug interactions,” and “therapeutic applications.”

Reference lists of relevant primary studies and review articles were also manually screened to identify additional publications not retrieved through the electronic searches. The search covered the period from database inception to 25 July 2026. Studies from all publication years were eligible, although greater emphasis was placed on the literature from the preceding 20–25 years to reflect major advances in the molecular, pharmacological, and translational investigation of hyperforin.

### 2.3. Eligibility Criteria

Peer-reviewed English-language publications were eligible if they provided original or synthesized evidence concerning the biological, pharmacological, therapeutic, mechanistic, pharmacokinetic, or safety-related properties of hyperforin. Eligible evidence included in vitro and animal studies, translational and pharmacokinetic investigations, observational and interventional human studies, randomized controlled trials (RCTs), systematic reviews, and meta-analyses. Studies were required to evaluate hyperforin directly or to report findings relevant to hyperforin-containing preparations, including *H. perforatum* extracts, with the intervention type explicitly considered during evidence interpretation.

Non-peer-reviewed reports, conference abstracts lacking sufficient methodological or outcome data, editorials, commentaries, opinion articles, and letters without original scientific evidence were excluded. Studies focusing exclusively on other constituents of *H. perforatum* without information relevant to hyperforin were also excluded, as were duplicate publications, overlapping datasets, and articles for which full-text access was unavailable.

### 2.4. Literature Selection and Information Extraction

Following the literature search, titles, abstracts, and full texts were reviewed by the authors for relevance to the objectives of this narrative review. Rather than applying a formal systematic-review selection procedure, publications were considered according to their scientific relevance, intervention characteristics, contribution to the thematic synthesis, and qualitative methodological robustness using study-design-specific considerations described below.

Methodological rigor was considered qualitatively using criteria appropriate to each study design rather than through a formal risk-of-bias scoring system. For in vitro studies, consideration was given to the suitability of the experimental model, use of appropriate controls, characterization and concentration of the tested intervention, adequacy of outcome assessment, and reproducibility or consistency of the reported effects. Animal studies were considered with respect to the suitability of the disease model, use of appropriate control groups, sample size where reported, intervention dose and duration, outcome assessment, and consistency between mechanistic and phenotypic findings. Observational human studies were interpreted according to sample size and population characteristics, adequacy of exposure and outcome assessment, consideration of relevant confounding factors, and appropriateness of the statistical analyses. Pharmacokinetic studies were considered with respect to characterization of the administered hyperforin or *H. perforatum* preparation, study population, dosing and sampling schedule, analytical methodology, and reporting of relevant pharmacokinetic parameters. For interventional and clinical studies, particular consideration was given to study design, randomization and blinding where applicable, sample size, comparator or control groups, intervention characterization, treatment duration, outcome assessment, attrition, and appropriateness of statistical analysis. These design-specific considerations informed the qualitative interpretation and relative weighting of the evidence but were not used to generate formal methodological-quality or risk-of-bias scores.

Study selection and interpretation were conducted collaboratively by the author team. Uncertainties or disagreements concerning study eligibility, relevance, intervention classification, interpretation of findings, or contribution to the thematic synthesis were resolved through detailed analytical discussion among all authors. Final decisions regarding inclusion and interpretation were reached by consensus after consideration of study design, methodological rigor, scientific relevance, intervention characteristics, and consistency with the broader evidence base.

For each included study, relevant information was extracted and thoroughly summarized, including study design and experimental model, sample characteristics where applicable, hyperforin source and formulation, dose and exposure conditions, principal molecular mechanisms or biological pathways, primary outcomes, therapeutic effects, safety observations, and main conclusions. The synthesis also considered recurring mechanisms, therapeutic applications, translational limitations, and areas of consistency or controversy across the literature.

### 2.5. Evidence Synthesis

Given the heterogeneity of the available literature in study design, experimental models, interventions, outcome measures, and disease contexts, quantitative synthesis was not considered appropriate. Evidence was therefore synthesized narratively through thematic integration of mechanistic, preclinical, translational, and clinical findings.

The synthesis was organized around three complementary levels of evidence. First, mechanistic studies were evaluated to identify the principal molecular and cellular pathways underlying the pharmacological actions of hyperforin. Second, disease-specific cellular, animal, and human evidence was integrated to assess biological plausibility, preclinical efficacy, and the extent of clinical validation across the major therapeutic domains considered in this review. Third, pharmacokinetic, formulation, safety, drug-interaction, and regulatory evidence was examined to assess translational feasibility and identify barriers to clinical development.

Throughout the synthesis, particular attention was given to the identity of the intervention and maturity of the supporting evidence. Findings obtained with purified hyperforin were distinguished from those derived from hyperforin-containing *H. perforatum* preparations, and mechanistic or preclinical activity was not considered equivalent to clinical efficacy. Where direct hyperforin-specific evidence was limited, relevant studies of *H. perforatum* preparations were considered but interpreted according to their multicomponent nature. Evidence was critically compared for consistency across experimental systems, disease relevance, intervention specificity, concordance between mechanistic, animal, and human findings, and overall translational significance.

Where exposure information was available, translational interpretation also considered whether experimentally active doses or concentrations were compatible with reported in vivo or human pharmacokinetic exposure; effects demonstrated only under substantially higher or poorly comparable exposure conditions were weighted primarily as mechanistic evidence rather than evidence of clinically achievable target engagement.

### 2.6. Methodological Limitations of the Review

Several methodological limitations should be considered when interpreting this review. First, although a structured literature search, predefined eligibility criteria, and a transparent study-selection process were applied, this work was designed as a narrative rather than a systematic review. Accordingly, procedures specific to systematic reviews, including duplicate independent screening, formal risk-of-bias assessment, certainty-of-evidence grading, and quantitative meta-analysis, were not undertaken. Accordingly, the design-specific methodological considerations described in Section 2.4 were used to support qualitative interpretation and weighting of the evidence rather than to provide a formal risk-of-bias classification. Although study selection and interpretation were conducted collaboratively and disagreements were resolved through analytical discussion and consensus among the authors, some influence of reviewer judgment on literature selection and thematic synthesis cannot be excluded.

Second, substantial methodological and clinical heterogeneity limited direct comparison across studies and precluded meaningful quantitative synthesis. The available evidence varied considerably in experimental models, study populations, interventions and formulations, doses and exposure conditions, treatment duration, comparators, and outcome measures. Consequently, this review emphasizes qualitative critical synthesis and the convergence of evidence across mechanistic, preclinical, translational, and clinical levels rather than pooled estimates of therapeutic efficacy.

Finally, publication and selective-reporting biases cannot be excluded. Positive or statistically significant findings are generally more likely to be published than neutral or negative results, potentially leading to overrepresentation of favorable evidence and overestimation of therapeutic effects [17,18,19]. Selective outcome reporting and dissemination bias may further affect the completeness and balance of the available literature [17,18,19]. These limitations should therefore be considered when interpreting the overall evidence and translational conclusions of this review.

## 3. Natural Sources, Biosynthesis and Pharmacokinetics of Hyperforin

### 3.1. Occurrence in Hypericum Species

Hyperforin is a specialized polyprenylated acylphloroglucinol occurring predominantly within the genus *Hypericum* (*Hypericaceae*), with *H. perforatum* L. (St. John’s Wort) representing its principal natural source and the most extensively investigated species from phytochemical and pharmacological perspectives [7,8]. Although hyperforin and structurally related polyprenylated phloroglucinols have also been identified in species such as *H. hirsutum*, *H. maculatum*, *H. olympicum*, *H. richeri*, *H. calycinum*, and *H. androsaemum*, their reported concentrations are generally lower than those in *H. perforatum*, limiting their significance as alternative commercial sources [8,20,21,22]. Consequently, *H. perforatum* remains the predominant source for the production of hyperforin-containing extracts and phytopharmaceutical preparations.

The occurrence of hyperforin within the genus Hypericum should nevertheless be considered within the broader phytochemical diversity of polyprenylated acylphloroglucinols (PPAPs). Bridi et al. provided a comprehensive survey of phloroglucinol derivatives reported across *Hypericum* species, demonstrating the considerable structural diversity of this metabolite class and providing an important basis for evaluating the taxonomic distribution of hyperforin and related compounds [22]. Subsequent phytochemical investigations have further expanded knowledge of PPAP diversity within the genus [22]. Available evidence nevertheless indicates that *H. perforatum* remains distinctive as the species in which hyperforin occurs as a quantitatively prominent constituent, whereas its occurrence in other *Hypericum* species is generally more limited and variable. This distinction is relevant not only phytochemically but also translationally, because botanical source, genotype, tissue, developmental stage, and environmental conditions can influence the hyperforin content of preparations used in pharmacological studies.

Hyperforin accumulation within *H. perforatum* is highly tissue-specific. The highest concentrations are generally found in reproductive tissues, particularly flowers, floral buds, and immature fruits, whereas leaves and young aerial shoots contain lower amounts and roots contain only trace levels [21,22]. Biosynthesis and accumulation occur predominantly within specialized secretory structures known as translucent glands distributed throughout the aerial tissues, whose density, developmental stage, and metabolic activity contribute to variations in hyperforin content among plant populations and cultivars [20,21,22,23].

**Figure 1:**
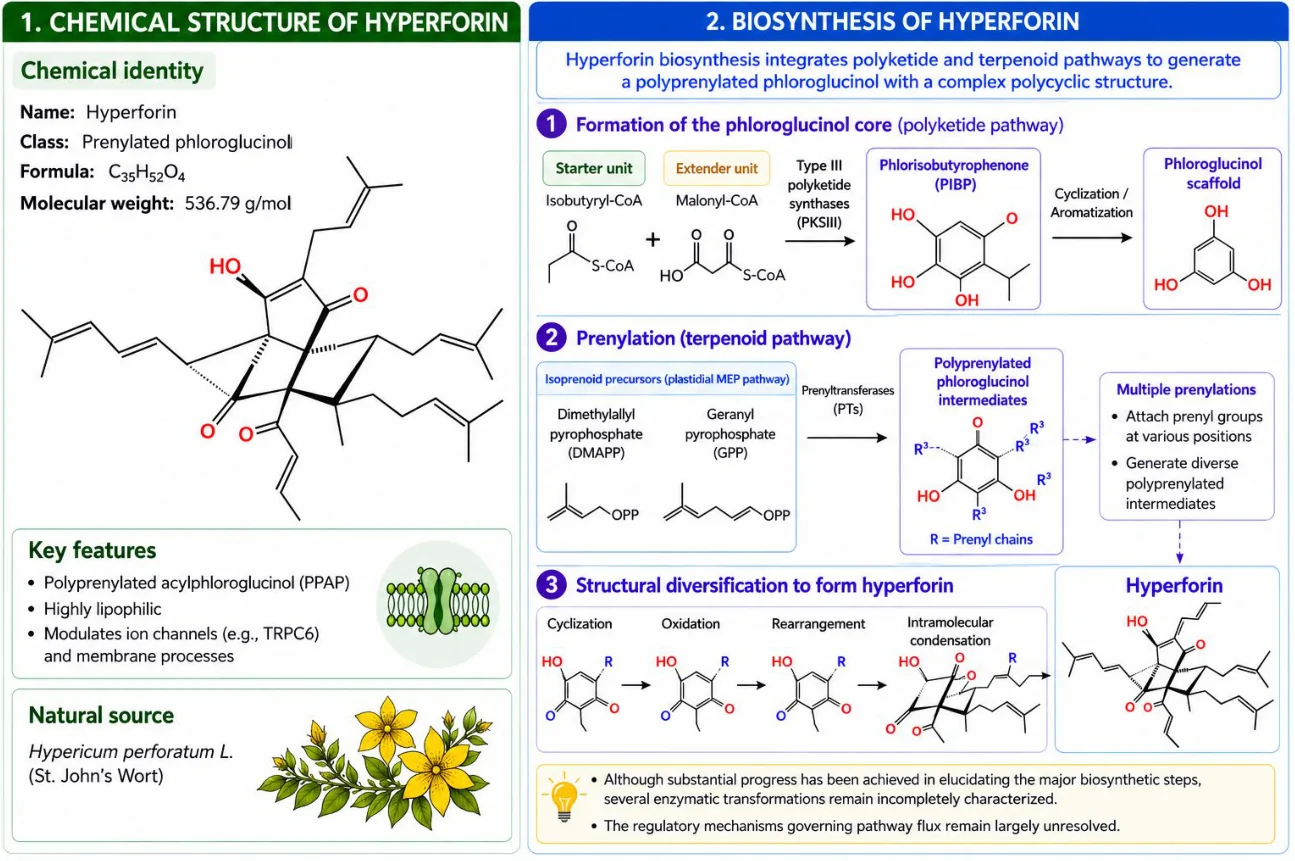
Chemical structure and schematic representation of the proposed biosynthetic pathway of hyperforin in *H. perforatum*. Hyperforin biosynthesis integrates (1) polyketide-derived formation of the phloroglucinol core, (2) terpenoid-derived prenylation involving DMAPP- and GPP-derived precursors, and (3) subsequent structural diversification leading to the hyperforin scaffold. Several enzymatic steps and regulatory mechanisms remain incompletely characterized.

Hyperforin concentrations are also influenced by genetic, environmental, developmental, geographical, and agronomic factors [7,8]. Environmental conditions, including light, temperature, water availability, soil characteristics, altitude, and seasonal variation, can substantially affect its biosynthesis and accumulation [20,21,22,23,24]. Concentrations generally increase during plant development and frequently reach their highest levels around flowering, making harvest timing an important determinant of extract composition, quality, and standardization [20,21,22,23,24]. These sources of phytochemical variability are particularly relevant when comparing hyperforin-containing botanical preparations across experimental and clinical studies. The chemical structure and proposed biosynthetic pathway of hyperforin are illustrated in Figure 1.

### 3.2. Biosynthesis of Hyperforin

The proposed biosynthetic pathway of hyperforin, schematically illustrated in Figure 1, integrates polyketide- and terpenoid-derived metabolic routes. Its characteristic polycyclic structure is generated through formation of a phloroglucinol-derived core, followed by extensive prenylation and subsequent structural modifications [25,26,27]. A key intermediate is phlorisobutyrophenone (PIBP), produced by type III polyketide synthases through sequential condensation of isobutyryl-CoA with malonyl-CoA units to form the phloroglucinol scaffold [8,26,27].

Subsequent prenylation introduces multiple isoprenoid side chains derived from dimethylallyl pyrophosphate (DMAPP) and geranyl pyrophosphate (GPP), generated through the plastidial methylerythritol phosphate (MEP) pathway (Figure 1) [8,26,27]. Prenyltransferases catalyze attachment of these groups to the phloroglucinol nucleus, while further cyclization, oxidation, rearrangement, and condensation reactions contribute to the structural diversification and formation of the complex hyperforin scaffold [8,26,27,28]. Although the principal biosynthetic framework has been established, several enzymatic transformations and regulatory mechanisms controlling pathway flux remain incompletely characterized.

Advances in genomic, transcriptomic, proteomic, and metabolomic approaches have facilitated the identification of candidate biosynthetic genes and regulatory elements involved in hyperforin production [26,27,29,30]. These findings also provide a basis for metabolic-engineering strategies using plant cell cultures, synthetic biology, and heterologous expression systems, with the potential to support more sustainable and standardized hyperforin production [26,27,29,30].

### 3.3. Extraction and Purification Methods

Efficient extraction and purification of hyperforin are important for phytochemical investigation and the development of standardized pharmaceutical and nutraceutical preparations. Its pronounced lipophilicity, chemical instability, and susceptibility to oxidative degradation require extraction conditions that maximize recovery while preserving molecular integrity.

Conventional extraction commonly employs organic solvents, including ethanol, methanol, acetone, hexane, ethyl acetate, or their mixtures, with extraction efficiency influenced by solvent composition and processing conditions [31,32,33]. Ethanol is particularly suitable for pharmaceutical applications because of its extraction efficiency, relatively low toxicity, and regulatory acceptability. Supercritical fluid extraction (SFE) with carbon dioxide (CO_2_) provides an alternative approach offering enhanced selectivity, minimal solvent residues, lower processing temperatures, and improved preservation of thermolabile constituents [29,33,34]. The addition of polar co-solvents such as ethanol can further enhance the recovery of hyperforin-enriched fractions [35,36], supporting the application of SFE for standardized *H. perforatum* extracts [19,33].

Advanced extraction approaches, including ultrasound-assisted, microwave-assisted, pressurized-liquid, enzyme-assisted, and accelerated-solvent extraction, have also been investigated to improve extraction efficiency while reducing processing time and solvent consumption [33,37,38]. Following extraction, chromatographic techniques such as preparative high-performance liquid chromatography (HPLC), flash chromatography, and counter-current chromatography can be used to obtain highly purified hyperforin for analytical, pharmacological, and pharmaceutical applications [33,37].

Importantly, extraction, purification, standardization, and formulation procedures can influence both hyperforin concentration and the relative abundance of other bioactive constituents in *H. perforatum* preparations. Consequently, pharmacological or clinical effects observed with multicomponent preparations cannot necessarily be attributed specifically to hyperforin, because coexisting phytochemicals and their interactions may contribute to the observed response. Such compositional variability represents an important source of heterogeneity across preclinical and clinical studies and should therefore be considered when interpreting the evidence.

### 3.4. Pharmacokinetics and Bioavailability

The pharmacokinetic profile of hyperforin is a major determinant of its translational potential but also represents an important obstacle to clinical development. Despite its broad experimental biological activity, therapeutic application is complicated by poor and variable oral bioavailability, pronounced lipophilicity, limited aqueous solubility, extensive tissue distribution, chemical and metabolic instability, and clinically relevant drug-interaction potential. Collectively, these characteristics complicate dose optimization and the establishment of reproducible systemic and tissue exposure.

Following oral administration, hyperforin is absorbed through the gastrointestinal tract, but its pronounced lipophilicity and very low aqueous solubility limit dissolution and contribute to incomplete and variable absorption [29,38,39]. Dietary lipids may enhance absorption through micellar solubilization and lymphatic transport, although this does not appear to eliminate formulation-dependent variability [29,38,39]. Consequently, systemic exposure may vary substantially among individuals and preparations.

Notably, human pharmacokinetic data, although limited, provide direct evidence that hyperforin reaches measurable systemic concentrations following oral administration of standardized *H. perforatum* preparations. In two open phase-I studies in healthy male volunteers, Schulz et al. (2005) evaluated the pharmacokinetics of hyperforin and other constituents following single-dose administration and once-daily administration for 14 days of a standardized *H. perforatum* extract [40]. Following a single dose, hyperforin reached a maximum plasma concentration of approximately 83.5 ng/mL at approximately 4.4 h, with an elimination half-life of approximately 19.6 h [40]. These findings demonstrate systemic exposure after oral administration but relate to a multicomponent *H. perforatum* preparation rather than purified hyperforin. Consequently, they do not resolve the pharmacokinetic uncertainties surrounding purified hyperforin, formulation-dependent exposure, or the relationship between circulating concentrations and pharmacologically active tissue concentrations.

A fundamental limitation in interpreting the pharmacokinetic literature is the lack of direct human comparisons between purified hyperforin and hyperforin administered within standardized botanical preparations. Pharmacokinetic parameters obtained with *H. perforatum* extracts cannot necessarily be extrapolated to isolated hyperforin because the surrounding phytochemical and formulation matrix may influence dissolution, gastrointestinal solubilization, intestinal permeability, metabolic stability, transporter activity, and ultimately systemic exposure. Conversely, purified hyperforin is highly lipophilic, poorly water-soluble, and chemically unstable, properties that may result in substantially different absorption and disposition when it is administered without the extract matrix or without an optimized pharmaceutical delivery system. Differences among commercial and experimental *H. perforatum* preparations in hyperforin content, extraction procedure, excipients, coexisting phytochemicals, and storage stability may further explain variability in reported pharmacokinetic parameters. At present, therefore, the available human pharmacokinetic evidence should primarily be interpreted as describing the disposition of hyperforin delivered within specific *H. perforatum* preparations rather than defining the intrinsic pharmacokinetic profile of purified hyperforin. Direct head-to-head pharmacokinetic studies using chemically characterized purified hyperforin and standardized extracts are needed to resolve this distinction.

Once absorbed, hyperforin exhibits extensive tissue distribution because of its affinity for lipid-rich biological membranes [14,41,42]. Its ability to cross the blood–brain barrier is relevant to its neuropsychiatric and neuroprotective pharmacology [14,41,42]. However, extensive tissue partitioning and high plasma protein binding may influence the free circulating fraction, tissue exposure, and elimination kinetics, further complicating the relationship between administered dose and biological response [14,41,42].

Hepatic metabolism is another major determinant of hyperforin disposition. In addition to undergoing biotransformation by cytochrome P450 (CYP450) enzymes, hyperforin activates the pregnane X receptor (PXR), leading to induction of CYP3A4, P-glycoprotein, and other xenobiotic-metabolizing systems [42,43,44]. This mechanism contributes substantially to the clinically relevant herb–drug interactions associated with hyperforin-containing *H. perforatum* preparations and may reduce exposure to co-administered medications [42,43,44], representing an important consideration particularly in patients receiving multiple pharmacological treatments.

Elimination appears to occur predominantly through biliary excretion, with a smaller contribution from renal clearance of metabolites [29,39,43]. However, several aspects of hyperforin disposition remain insufficiently characterized, including absolute oral bioavailability, elimination kinetics, tissue-specific distribution, interindividual variability, and the influence of formulation on systemic exposure.

Chemical instability further complicates the pharmacokinetic and pharmaceutical profile of hyperforin. Oxidative degradation during extraction, storage, formulation, and physiological exposure may reduce biological activity and contribute to variability among experimental and commercial preparations [10,31,45]. Hyperforin is also photosensitive, with ultraviolet and visible light accelerating its degradation [10,31,45]. Appropriate manufacturing, storage, packaging, and formulation conditions are therefore required to preserve molecular stability and improve batch-to-batch consistency.

**Figure 2:**
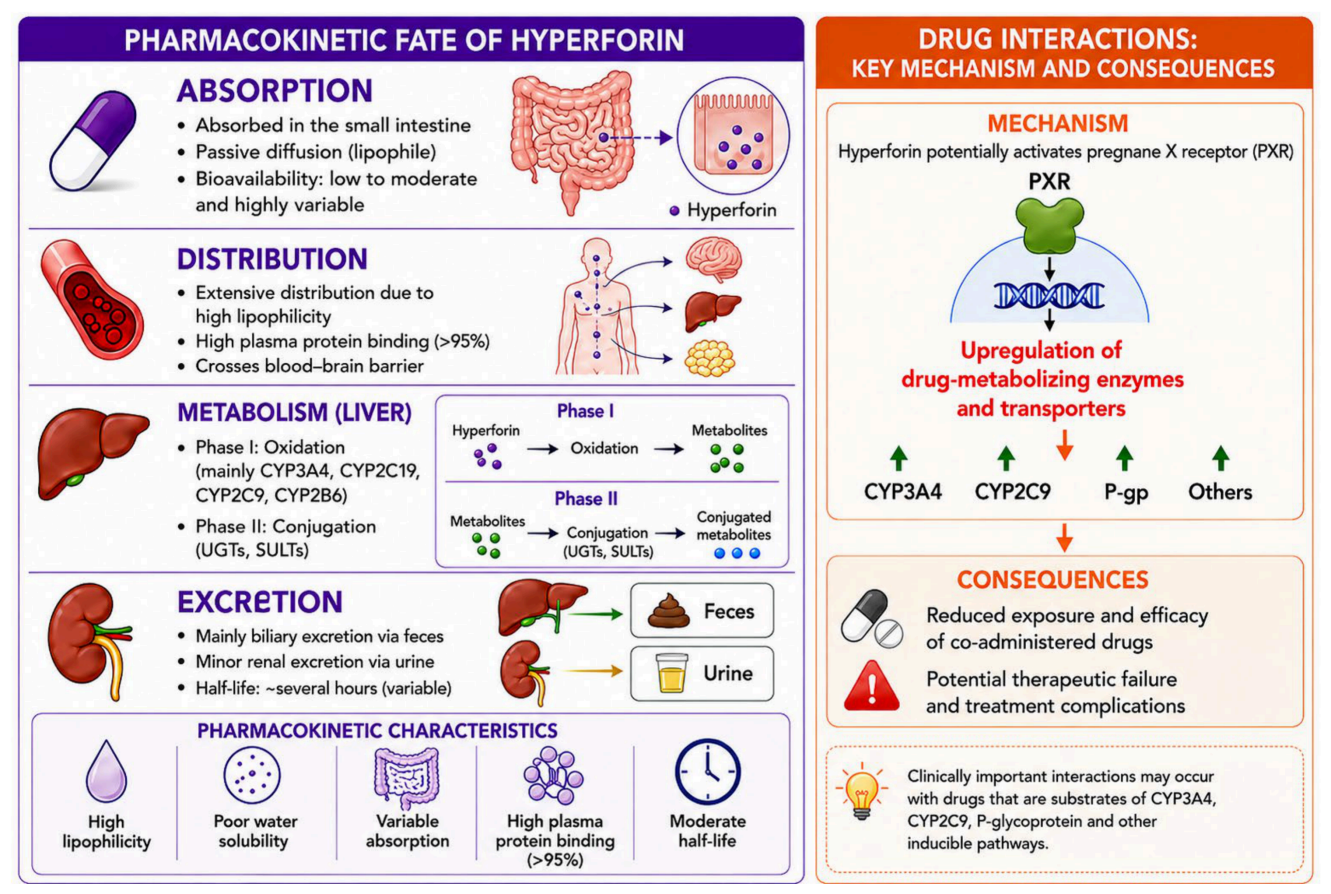
Schematic representation of the reported pharmacokinetic disposition of hyperforin and its principal drug-interaction mechanisms. Hyperforin activates PXR and induces drug-metabolizing enzymes and transporters, particularly CYP3A4 and P-glycoprotein. Several aspects of its human pharmacokinetic profile remain incompletely characterized and may vary according to formulation and exposure.

Overall, poor solubility, variable absorption, extensive tissue distribution, metabolic complexity, chemical instability, and interaction liability limit the establishment of reproducible dose–exposure–response relationships. Further human pharmacokinetic studies should therefore characterize formulation-dependent exposure, interindividual variability, and clinically relevant dosing of purified hyperforin. The principal pharmacokinetic and drug-interaction mechanisms are summarized in Figure 2.

### 3.5. Advanced Delivery Systems

Given the poor aqueous solubility, pronounced lipophilicity, and chemical instability of hyperforin, advanced drug-delivery systems have been investigated to improve its formulation stability, dispersion, cellular delivery, and pharmacological performance. However, evidence remains predominantly experimental, and whether these approaches provide reproducible improvements in human systemic exposure or clinical efficacy has not yet been established.

Nanotechnology-based formulations, including polymeric nanoparticles, solid lipid nanoparticles, and related nanostructured lipid carriers, may improve the dispersion of hyperforin, protect it from chemical degradation, enhance cellular uptake, and enable controlled or sustained release [46]. Experimental studies suggest that nanoparticle encapsulation can enhance its biological activity under selected conditions, although evidence for consistent improvements in systemic pharmacokinetics, tissue exposure, or in vivo therapeutic efficacy remains limited [46].

Liposomal and other phospholipid-based delivery systems have also been proposed for hyperforin because of their capacity to incorporate highly lipophilic compounds and potentially improve stability, membrane interaction, cellular uptake, and gastrointestinal dispersion [43,47,48]. Phytosome technology may similarly facilitate the pharmaceutical delivery of poorly water-soluble phytochemicals through complexation with phospholipids [43,48]. However, direct evidence demonstrating substantial and reproducible improvements in the systemic bioavailability of hyperforin remains insufficient.

Nanoemulsion-based formulations represent another experimental approach that may improve aqueous dispersion, apparent solubilization, and dissolution and can potentially support oral, topical, transdermal, or parenteral administration [49,50]. Selected experimental studies have reported improved pharmaceutical or biological performance of hyperforin-containing nanoemulsions compared with conventional preparations [49,50].

Overall, advanced delivery technologies provide promising strategies for addressing some of the physicochemical and formulation limitations of hyperforin, but they should not yet be regarded as established solutions to its bioavailability constraints. Their translational value requires confirmation through direct comparison of chemically characterized formulations, standardized stability and release assessment, and hyperforin-specific in vivo and human pharmacokinetic studies capable of establishing reproducible dose–exposure relationships. Importantly, enhanced systemic exposure may also modify the safety and drug-interaction profile of hyperforin because of its capacity to activate PXR and induce drug-metabolizing enzymes and transporters. Formulation optimization should therefore be accompanied by appropriate pharmacokinetic, safety, and drug-interaction evaluation before these technologies can support the clinical development of purified hyperforin.

## 4. Mechanisms of Action of Hyperforin: Potential Biological Targets and Regulatory Systems

### 4.1. Basic Principles

Experimental investigation of hyperforin mechanisms has relied predominantly on in vitro cellular systems and in vivo animal models. Most mechanistic in vitro studies have examined purified hyperforin, although hyperforin-containing *H. perforatum* preparations or formulations have also been investigated, and both intervention types have been evaluated in animal studies. Accordingly, mechanistic findings are interpreted throughout this section according to the intervention studied, and effects of multicomponent *H. perforatum* preparations are not attributed exclusively to hyperforin.

Available evidence indicates that hyperforin acts as a pleiotropic signaling modulator rather than a conventional single-target pharmacological agent, influencing interconnected systems involved in neurotransmission, Ca^2+^ homeostasis, neuroplasticity, inflammatory and redox regulation, mitochondrial function, programmed cell death, autophagy, epigenetic regulation, and cellular metabolism. Transient receptor potential cation channel subfamily C member 6 (TRPC6)-mediated Ca^2+^ signaling represents an important component of hyperforin pharmacology, although its contribution varies according to cell type, tissue, experimental conditions, and biological outcome. TRPC6 should therefore be considered within a broader network of interacting pathways rather than as a universal mechanism underlying all reported effects of hyperforin.

The mechanistic framework adopted here distinguishes, where supported by the evidence, primary molecular interactions and proximal signaling events from downstream cellular responses and broader biological outcomes. Accordingly, modulation of pathways such as nuclear factor kappa B (NF-κB), nuclear factor erythroid 2-related factor 2 (Nrf2), phosphoinositide 3-kinase/protein kinase B (PI3K/Akt), mitogen-activated protein kinase/extracellular signal-regulated kinase (MAPK/ERK), mitochondrial signaling, autophagy, and apoptosis should not necessarily be interpreted as evidence of direct molecular targeting by hyperforin. The following subsections examine these mechanisms according to the nature and strength of the supporting experimental evidence.

An additional consideration in interpreting the mechanistic literature is the relationship between experimentally active concentrations and pharmacologically achievable human exposure. Cellular and biochemical studies are valuable for establishing biological plausibility and defining concentration-dependent molecular responses; however, effects observed at experimental concentrations should not automatically be interpreted as evidence of target engagement under clinically achievable exposure conditions. Where human pharmacokinetic or relevant in vivo exposure data are available, greater translational weight is therefore given to mechanisms supported at exposure levels compatible with those data. Conversely, mechanisms demonstrated predominantly at concentrations substantially exceeding, or not directly comparable with, documented human exposure are interpreted primarily as evidence of biological capability rather than established clinically achievable pharmacology. This distinction is particularly important because the tissue concentrations of purified hyperforin required for engagement of many proposed targets remain incompletely characterized.

**Figure 3:**
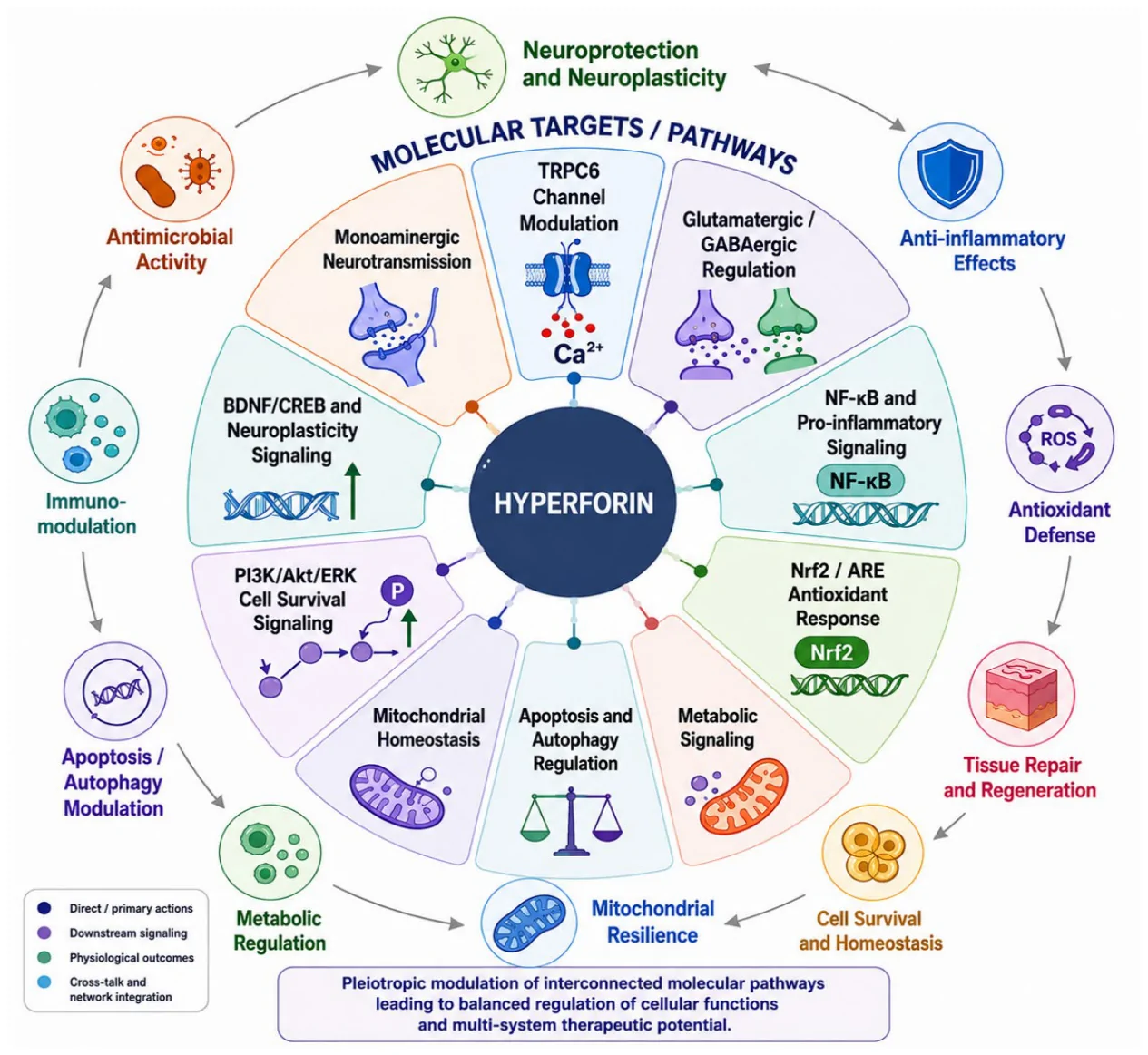
Systems-level molecular pharmacology of hyperforin and convergence of its principal signaling pathways on cellular homeostasis. Hyperforin modulates multiple interconnected molecular and cellular systems, including TRPC6-mediated Ca^2+^ signaling, monoaminergic and glutamatergic/GABAergic neurotransmission, BDNF/CREB-associated neuroplasticity, PI3K/Akt/ERK signaling, NF-κB-mediated inflammatory responses, Nrf2-associated antioxidant defenses, mitochondrial homeostasis, apoptosis/autophagy, and metabolic signaling. These pathways converge on broader biological processes relevant to neuroplasticity and neuroprotection, inflammatory and redox homeostasis, immunomodulation, mitochondrial resilience, cellular survival, metabolic regulation, antimicrobial activity, and tissue repair. The diagram represents an integrative conceptual synthesis; the strength of experimental evidence differs among individual pathways and biological outcomes.

Figure 3 integrates the principal molecular pathways discussed in this section and their convergence on broader biological processes. It should be interpreted as a conceptual synthesis of hyperforin pharmacology rather than as evidence that all depicted pathways are direct molecular targets or have equivalent levels of experimental or clinical validation.

### 4.2. Modulation of Neurotransmitter Systems

Modulation of neurotransmitter homeostasis is among the most extensively characterized pharmacological actions of hyperforin. Unlike conventional antidepressants that primarily target individual monoamine transporters, hyperforin exhibits a broader neuromodulatory profile involving serotonergic, dopaminergic, noradrenergic, gamma-aminobutyric acid-ergic (GABAergic), and glutamatergic neurotransmission [14,51,52].

Mechanistically, hyperforin appears to inhibit neurotransmitter uptake predominantly through alterations in transmembrane sodium and calcium gradients rather than through direct competitive inhibition of transporter proteins [14,52,53,54]. Disruption of the ion gradients required for transporter function increases extracellular concentrations of serotonin, dopamine, and norepinephrine, thereby enhancing monoaminergic neurotransmission [14,52,53,54]. This indirect mechanism distinguishes hyperforin from selective serotonin reuptake inhibitors (SSRIs) and contributes to its distinct pharmacodynamic profile [14,52,53,54].

Hyperforin also modulates inhibitory and excitatory neurotransmission. Effects on gamma-aminobutyric acid (GABA) signaling may contribute to the regulation of neuronal excitability, whereas modulation of glutamatergic neurotransmission may influence synaptic plasticity and neuronal survival [54,55]. In particular, regulation of glutamate homeostasis may be relevant to protection against excitotoxic neuronal injury [54,55]. Collectively, these findings support a multimodal effect of hyperforin on neurotransmitter systems, while the functional and therapeutic consequences of these mechanisms are considered in the disease-specific sections below.

### 4.3. TRPC6 Channel Activation

Activation of TRPC6 channels represents one of the best-characterized molecular mechanisms associated with hyperforin pharmacology [55,56,57]. TRPC6 is a non-selective, calcium-permeable cation channel expressed in the central nervous system and other tissues, where it participates in processes including neuronal differentiation, synaptic function, dendritic development, and cell survival [55,56,57].

Hyperforin acts as a potent activator of TRPC6 channels, promoting Ca^2+^ influx and subsequent activation of calcium-dependent signaling pathways [57,58,59]. Under experimental conditions, TRPC6-mediated Ca^2+^ entry has been associated with activation of calcium/calmodulin-dependent protein kinase II (CaMKII), cyclic AMP response element-binding protein (CREB), extracellular signal-regulated kinases (ERK1/2), PI3K/Akt, and brain-derived neurotrophic factor (BDNF)-related signaling [57,58,59]. These downstream responses are linked to neuronal growth, dendritic and synaptic remodeling, and mechanisms supporting neuronal adaptation and survival [57,58,59].

Experimental studies further associate hyperforin-related TRPC6 signaling with enhanced dendritic spine formation, synaptic density, and neuronal connectivity [14,57,60], as well as increased resistance to oxidative, mitochondrial, excitotoxic, and apoptotic stress [14,57,60]. Collectively, these findings support TRPC6-mediated Ca^2+^ signaling as an important mechanistic component linking hyperforin exposure to neuroplastic and neuroprotective responses. However, TRPC6 activation should be interpreted within the broader signaling network described in Section 4.1 rather than as a universal mechanism accounting for all biological or therapeutic effects attributed to hyperforin.

Notably, although TRPC6 represents one of the most consistently supported proximal targets of hyperforin, the concentration–exposure relationship required for sustained TRPC6 engagement in human tissues has not been adequately defined; therefore, robust experimental evidence for TRPC6 activation should not yet be interpreted as confirmation of clinically achievable target engagement.

### 4.4. Anti-Inflammatory Mechanisms

Hyperforin exerts anti-inflammatory effects through coordinated modulation of multiple signaling pathways rather than selective inhibition of a single inflammatory mediator. Among the best-characterized mechanisms is suppression of NF-κB signaling. Hyperforin inhibits NF-κB activation and nuclear translocation, thereby attenuating transcriptional programs involved in the production of inflammatory mediators [5,60].

Hyperforin also modulates MAPK pathways, including ERK, c-Jun N-terminal kinase (JNK), and p38 MAPK, which participate in inflammatory gene expression and immune-cell responses [5,60,61]. Consistent with these signaling effects, experimental studies report reduced production of major pro-inflammatory cytokines, including tumor necrosis factor-alpha (TNF-α), interleukin-1 beta (IL-1β), and IL-6 [14,51,60].

Additional anti-inflammatory activity involves suppression of cyclooxygenase-2 (COX-2) expression and activity, with consequent modulation of pro-inflammatory prostaglandin synthesis [5,62,63]. These mechanisms interact with the antioxidant and metabolic effects of hyperforin, supporting an interconnected rather than pathway-specific model of its biological activity [5,62,63]. Collectively, regulation of NF-κB and MAPK signaling, cytokine production, and eicosanoid pathways provides a mechanistic basis for the anti-inflammatory effects observed with hyperforin-related interventions, although their disease-specific and clinical relevance depends on the experimental context and level of supporting evidence.

### 4.5. Antioxidant Mechanisms

Hyperforin exhibits antioxidant activity through complementary mechanisms involving attenuation of reactive oxygen species (ROS) accumulation, enhancement of endogenous antioxidant defenses, and preservation of mitochondrial homeostasis. Experimental studies using purified hyperforin and hyperforin-containing preparations have reported reduced intracellular ROS accumulation and oxidative damage to cellular macromolecules [64,65]. In parallel, these interventions may enhance endogenous antioxidant capacity through upregulation of key antioxidant enzymes, including superoxide dismutase, catalase, and glutathione peroxidase, together with modulation of the glutathione redox system [61,65].

Mitochondrial regulation represents an additional component of the antioxidant response associated with hyperforin. Experimental evidence indicates preservation of mitochondrial membrane integrity, attenuation of mitochondrial ROS production, stabilization of electron transport chain function, and reduced mitochondrial permeability transition [66,67]. These effects may limit the amplification of oxidative injury and contribute to maintenance of cellular redox and mitochondrial homeostasis.

Collectively, the available mechanistic evidence supports modulation of oxidative stress and mitochondrial function as interconnected components of hyperforin-related cytoprotective activity. However, these findings are derived predominantly from experimental models, and their contribution to disease-specific therapeutic effects should be interpreted according to the intervention studied, biological context, and strength of the supporting evidence.

### 4.6. Regulation of Apoptosis

Hyperforin exhibits context-dependent effects on apoptotic signaling, with experimental studies indicating cytoprotective effects under selected pathological stress conditions and pro-apoptotic activity in transformed or malignant cells [66,67,68,69,70]. This differential response appears to involve modulation of both intrinsic and extrinsic apoptotic pathways.

Within the intrinsic mitochondrial pathway, hyperforin modulates the balance between anti-apoptotic and pro-apoptotic Bcl-2 family proteins, including Bcl-2 and Bax, thereby influencing cellular susceptibility to apoptosis [66,67,68,69,71,72]. In cancer-cell models, hyperforin has been associated with mitochondrial membrane depolarization, cytochrome c release, apoptosome formation, and activation of downstream caspases, particularly caspase-9 and caspase-3 [67,69,70,73,74].

Hyperforin may also modulate extrinsic apoptotic signaling involving death receptors and ligand-mediated apoptotic cascades [69,75,76]. Collectively, these findings indicate that regulation of programmed cell death represents an important component of hyperforin pharmacology. However, the direction and magnitude of these effects appear to depend on cell type and biological context, and evidence for selective induction of apoptosis in malignant cells versus protection from pathological apoptosis in non-malignant tissues remains predominantly experimental [66,67,68,69,70,71,72,73,74,75,76].

### 4.7. Modulation of Autophagy

Emerging experimental evidence suggests that hyperforin modulates autophagic pathways through regulatory networks involving PI3K/Akt/mTOR and AMP-activated protein kinase (AMPK) signaling [67,77]. Through modulation of autophagic flux, hyperforin may facilitate the clearance of damaged proteins and organelles, thereby supporting intracellular quality control, proteostasis, and cellular adaptation to stress [67,77].

These effects may be particularly relevant in neuronal cells, where impaired autophagy contributes to the accumulation of toxic proteins and dysfunctional cellular components associated with neurodegenerative disorders, including Alzheimer’s, Parkinson’s, and Huntington’s diseases [78,79]. Experimental findings suggest that enhancement of cellular clearance mechanisms and attenuation of proteotoxic stress may contribute to the neuroprotective effects associated with hyperforin [55,67,77].

Overall, autophagy modulation represents a potentially important component of hyperforin-related cytoprotective activity. However, the available evidence remains predominantly experimental, and the extent to which hyperforin directly regulates autophagic machinery, rather than influencing autophagy secondarily through upstream signaling networks, requires further clarification.

### 4.8. Epigenetic and Gene Regulatory Effects

Hyperforin influences gene-regulatory networks associated with inflammation, oxidative stress, apoptosis, neuroplasticity, and metabolic adaptation. Experimental evidence indicates modulation of several transcription factors, including NF-κB, CREB, activator protein-1 (AP-1), nuclear factor erythroid 2-related factor 2 (Nrf2), and signal transducer and activator of transcription (STAT) proteins [55,59,67]. These transcriptional effects may contribute to broader changes in cellular phenotype and to the pleiotropic biological responses associated with hyperforin [55,59,67].

Evidence for direct epigenetic regulation is less established. Preliminary experimental findings suggest that hyperforin may influence histone modifications, chromatin remodeling, and DNA accessibility, potentially producing more sustained alterations in gene expression [65,67,77]. Hyperforin has also been associated with changes in microRNA expression profiles, providing a potential mechanism for post-transcriptional regulation of genes involved in inflammatory, neurodegenerative, metabolic, and carcinogenic processes [80,81].

Collectively, these findings support gene-regulatory modulation as a component of hyperforin pharmacology, whereas its direct epigenetic effects remain comparatively underexplored. Further studies are required to distinguish primary epigenetic actions of hyperforin from downstream changes in gene expression arising from modulation of intracellular signaling pathways.

### 4.9. Effects on Cellular Energy Metabolism

Hyperforin modulates cellular bioenergetics primarily through effects on mitochondrial function and energy-sensing pathways. Experimental studies indicate that hyperforin preserves mitochondrial membrane potential, supports oxidative phosphorylation and electron transport chain function, and attenuates mitochondrial oxidative stress [67,82,83]. Collectively, these effects may support ATP production and cellular energy availability under conditions of metabolic or cellular stress.

Hyperforin also influences metabolic signaling pathways involved in energy sensing and nutrient utilization, particularly AMPK, PI3K/Akt signaling, and pathways associated with mitochondrial biogenesis [67,84]. Modulation of these interconnected networks may contribute to metabolic flexibility, adaptation to energetic stress, and maintenance of cellular energy homeostasis [67,84].

Overall, the available evidence supports mitochondrial and metabolic regulation as components of the broader pharmacological profile of hyperforin. However, these bioenergetic effects are derived predominantly from experimental models, and their relative contribution to the disease-specific actions of hyperforin remains to be established. Accordingly, modulation of mitochondrial function and energy-sensing pathways should be regarded as part of an interconnected signaling network rather than as evidence of a single, direct metabolic target.

### 4.10. Critical Appraisal of Mechanistic Evidence

The principal mechanisms of hyperforin and their associated biological targets and regulatory systems are summarized in Table 1. Although the experimental literature supports a broad and pleiotropic pharmacological profile, the strength and specificity of evidence vary considerably among proposed mechanisms.

Most mechanistic studies have been conducted in immortalized cell lines or experimental models that incompletely reproduce human pathophysiology, frequently using hyperforin concentrations that may not reflect physiologically achievable exposure. Accordingly, the breadth of mechanistic activity reported for hyperforin should not be equated with evidence that all of these pathways are engaged at clinically achievable systemic or tissue exposure. Mechanisms supported by concentration–response data overlapping with pharmacologically realistic exposure should be considered more translationally informative than effects demonstrated only at substantially higher or poorly characterized experimental concentrations. Moreover, relatively few studies have directly compared purified hyperforin with standardized hyperforin-containing *H. perforatum* preparations, limiting attribution of effects observed with multicomponent extracts specifically to hyperforin. Interpretation is further complicated by the frequent involvement of signaling pathways such as NF-κB, MAPK, PI3K/Akt, and AMPK, which often represent downstream regulatory networks rather than established direct molecular targets.

Among the proposed mechanisms, TRPC6-mediated Ca^2+^ signaling is one of the most consistently characterized proximal mechanisms of hyperforin action, whereas evidence for several other pathways is more context-dependent or may reflect secondary signaling responses. Accordingly, the current mechanistic evidence supports hyperforin as a pleiotropic signaling modulator rather than a single-target agent. Further studies using physiologically relevant concentration ranges, concentration–response designs, target-specific experimental approaches, human-relevant models, and well-characterized purified hyperforin are required to distinguish direct molecular interactions from downstream effects and to establish which mechanisms remain operative at clinically achievable exposure.

## 5. Neuroprotective and Mental Health Benefits

### 5.1. Depression

#### 5.1.1. Biological and Mechanistic Rationale

Major depressive disorder (MDD) involves disturbances in monoaminergic neurotransmission, neuroplasticity, inflammatory and redox homeostasis, mitochondrial function, HPA-axis regulation, and neurotrophic support [85,86,87,88]. The antidepressant rationale for hyperforin derives from the mechanisms detailed in Section 4, particularly indirect modulation of serotonin, dopamine, and norepinephrine uptake [53,55,83,89,90], together with TRPC6-mediated CREB/BDNF, ERK1/2, and PI3K/Akt signaling involved in neuroplasticity and stress adaptation [50,54,55,57,91,92]. Anti-inflammatory, antioxidant, and mitochondrial effects may provide additional support [93,94,95,96,97,98]. Collectively, these mechanisms provide a plausible experimental basis for antidepressant activity, although their relative contribution remains model-dependent.

#### 5.1.2. Preclinical Evidence of Purified Hyperforin and Hyperforin-Containing Preparations

Preclinical studies provide consistent evidence of antidepressant-like activity across several experimental paradigms. In rodent models, hyperforin-related interventions reduced depressive-like behavior, including immobility in forced-swimming and tail-suspension tests, and improved reward-related and motivational behaviors [54,97,98]. These behavioral effects have been associated with normalization of stress-related monoaminergic alterations, increased hippocampal BDNF expression, enhanced neurogenesis, and improved synaptic plasticity [54,91,97,98,99], together with attenuation of neuroinflammation and oxidative damage and preservation of mitochondrial integrity [91,95,96,98,100].

Overall, the preclinical evidence indicates that antidepressant-like effects are not limited to monoaminergic modulation but also involve neuroplastic, inflammatory, redox, and mitochondrial pathways. However, interpretation requires careful consideration of intervention identity because studies have employed both purified hyperforin and hyperforin-containing preparations; findings obtained with multicomponent *H. perforatum* extracts cannot be attributed exclusively to hyperforin.

#### 5.1.3. Clinical Evidence of Hyperforin-Containing Preparations

Human evidence supporting antidepressant efficacy derives predominantly from standardized hyperforin-containing *H. perforatum* preparations rather than purified hyperforin. Multiple RCTs, systematic reviews, and meta-analyses indicate efficacy in mild-to-moderate depression, with outcomes broadly comparable to several conventional antidepressants [101,102,103,104,105,106,107]. Several studies have also reported associations between hyperforin content and antidepressant efficacy [101,102,103,104,108,109]. However, because these preparations contain multiple pharmacologically active constituents, the independent contribution of hyperforin cannot be established. Adequately powered clinical trials evaluating purified hyperforin remain lacking, and detailed human evidence is reviewed in Section 12.2.

The clinical relevance of standardized *H. perforatum* preparations is also reflected in the European regulatory framework. The European Medicines Agency Committee on Herbal Medicinal Products (EMA/HMPC) has adopted a European Union herbal monograph for *Hypericum perforatum* L., herba, which recognizes well-established medicinal use for specified standardized herbal preparations in the treatment of mild to moderate depressive episodes and, for certain preparations, the short-term treatment of symptoms in mild depressive disorders [110]. This regulatory recognition supports the established clinical use of defined *H. perforatum* preparations in depressive disorders [110]. However, it should not be interpreted as regulatory validation of purified hyperforin as an antidepressant, because the monograph applies to characterized herbal preparations containing multiple constituents rather than isolated hyperforin.

### 5.2. Anxiety Disorders

#### 5.2.1. Preclinical Evidence of Purified Hyperforin and Hyperforin-Containing Preparations

Anxiety disorders involve dysregulation of limbic circuitry and neurotransmitter and stress-response systems, together with neuroinflammatory, oxidative, and HPA-axis alterations. These interconnected processes provide a biological rationale for the potential anxiolytic effects of hyperforin through the multimodal mechanisms described in Section 4.

Experimental animal studies indicate that purified hyperforin and hyperforin-containing preparations may reduce anxiety-like behavior through modulation of serotonergic, GABAergic, and glutamatergic neurotransmission [54,99,111,112]. These effects have been associated with improved stress resilience and normalization of chronic stress-induced neurochemical alterations [54,97,98,113], together with attenuation of inflammatory signaling and oxidative stress [93,95,98,114].

Overall, the preclinical evidence supports potential anxiolytic activity of hyperforin-related interventions, although the relative contributions of individual neurotransmitter, inflammatory, redox, and stress-response pathways remain incompletely defined. Moreover, findings obtained with multicomponent hyperforin-containing preparations cannot necessarily be attributed specifically to hyperforin.

#### 5.2.2. Potential Clinical Relevance of Hyperforin-Containing Preparations

Clinical evidence for anxiety-related outcomes is considerably less developed than that for depression. Available studies suggest that standardized hyperforin-containing *H. perforatum* preparations may improve anxiety-related symptoms, particularly in mixed anxiety–depressive presentations, with reported benefits in psychological distress, emotional well-being, and quality of life [101,115,116,117,118,119].

However, these studies predominantly evaluated multicomponent botanical preparations rather than purified hyperforin, precluding compound-specific attribution of the observed effects. Consequently, the clinical anxiolytic efficacy of purified hyperforin remains unestablished. Detailed human evidence concerning anxiety-related outcomes is presented in Section 12.3.1.

### 5.3. Cognitive Function, Synaptic Plasticity, and Neurogenesis

Experimental evidence suggests that purified hyperforin and hyperforin-containing preparations may improve learning, memory, and cognitive performance through the neuroplastic and neuroprotective mechanisms described in Section 4 [54,120,121,122,123,124]. Hyperforin-related interventions have been associated with enhanced TRPC6/CREB/BDNF signaling, dendritic spine formation, synaptogenesis, neuronal connectivity, and processes related to hippocampal neurogenesis [14,54,57,91,96,121]. Modulation of neurotransmission and protection against inflammatory, oxidative, and mitochondrial injury may further support neuronal survival and cognitive function [52,120,122]. However, the evidence remains predominantly experimental, and clinically meaningful cognitive or neurogenic effects in humans have not been established.

### 5.4. Alzheimer’s Disease

#### 5.4.1. Amyloid Pathology

Preclinical studies indicate that purified hyperforin and hyperforin-containing preparations may influence amyloid-β (Aβ) pathology by inhibiting Aβ aggregation, enhancing amyloid clearance, and attenuating Aβ-induced neurotoxicity [125,126,127,128]. Additional protection against mitochondrial and oxidative injury may limit secondary neuronal damage associated with amyloid accumulation [96,120,125,128]. Collectively, these findings suggest that hyperforin-related interventions may affect both amyloid burden and downstream Aβ toxicity; however, the evidence remains preclinical and does not yet establish disease-modifying activity in Alzheimer’s disease (AD).

#### 5.4.2. Tau Pathology

Emerging preclinical evidence suggests that purified hyperforin and hyperforin-containing preparations may modulate tau phosphorylation and cytoskeletal stability [127,129,130]. Experimental studies indicate attenuation of abnormal tau hyperphosphorylation through regulation of kinases and phosphatases involved in tau metabolism, with potential preservation of microtubule organization and neuronal structural integrity [129,130]. However, evidence for tau-related effects is substantially less developed than that for amyloid pathology and requires confirmation in additional disease-relevant models.

#### 5.4.3. Neuroinflammation and Neuronal Protection

Hyperforin-related interventions may additionally attenuate AD-associated neuroinflammatory responses through the anti-inflammatory and redox-regulatory mechanisms described in Section 4. Preclinical studies have reported suppression of NF-κB-associated signaling and reductions in TNF-α, IL-1β, IL-6, iNOS, and COX-2 [94,120,122]. These effects may complement the amyloid- and tau-related actions described above, although their specific contribution to AD remains uncertain because neuroinflammatory regulation represents a shared neuroprotective mechanism across several neurodegenerative disorders.

### 5.5. Parkinson’s Disease

#### 5.5.1. Dopaminergic Neuron Protection

Preclinical studies suggest that purified hyperforin and hyperforin-containing preparations may exert neuroprotective effects in experimental models relevant to Parkinson’s disease (PD). These interventions have been associated with preservation of dopaminergic neuronal function through modulation of neurotrophic signaling, inflammatory responses, and mitochondrial homeostasis [55,96,131,132]. TRPC6-associated Ca^2+^ signaling and downstream pro-survival pathways may further contribute to neuronal resilience under neurotoxic conditions [55,133,134,135].

Collectively, these findings provide a mechanistic and preclinical rationale for potential protection of dopaminergic neurons by hyperforin-related interventions. However, the available evidence remains predominantly experimental and does not establish disease-modifying efficacy in PD.

#### 5.5.2. Oxidative Stress and Mitochondrial Protection

Oxidative stress and mitochondrial dysfunction are closely associated with dopaminergic neuronal injury in PD. Experimental evidence indicates that purified hyperforin and hyperforin-containing preparations may reduce reactive oxygen species generation, enhance endogenous antioxidant defenses, and preserve mitochondrial membrane integrity and bioenergetic function [14,63,96,120,136]. These effects may limit oxidative and mitochondrial injury and thereby contribute to neuronal survival under conditions relevant to PD.

However, antioxidant and mitochondrial effects represent broadly shared cytoprotective mechanisms and are not specific to PD. Moreover, direct evidence that hyperforin modifies α-synuclein pathology or produces sustained protection of the nigrostriatal system remains limited. Thus, current findings support biological plausibility and preclinical neuroprotective potential rather than established disease-modifying activity.

### 5.6. Other Neurodegenerative Disorders

#### 5.6.1. Huntington’s Disease

Huntington’s disease (HD) is characterized by progressive neurodegeneration associated with mutant huntingtin accumulation, mitochondrial dysfunction, oxidative stress, impaired proteostasis, and neuroinflammatory processes [137]. Although direct evidence for hyperforin in HD is limited, its experimentally demonstrated effects on antioxidant defenses, mitochondrial function, inflammatory signaling, and autophagy provide a mechanistic rationale for potential neuroprotective activity [5,14].

In particular, modulation of autophagic and proteostatic pathways could theoretically facilitate the clearance of damaged proteins and cellular components. However, evidence that hyperforin directly promotes mutant huntingtin clearance, preserves vulnerable neuronal populations, or modifies HD progression is lacking. Its potential relevance to HD therefore remains largely mechanistic and hypothesis-generating.

#### 5.6.2. Multiple Sclerosis

Multiple sclerosis (MS) is a chronic inflammatory and demyelinating disorder involving immune dysregulation, oxidative injury, and progressive neuroaxonal damage [138]. Experimental studies suggest that hyperforin-related interventions may modulate inflammatory and immune responses by reducing pro-inflammatory cytokine production and immune-cell activation while attenuating oxidative stress [139,140,141]. These effects may contribute to the preservation of neuronal integrity under inflammatory conditions.

Nevertheless, the evidence remains predominantly preclinical, and it is unclear whether these mechanisms translate into meaningful effects on demyelination, remyelination, axonal preservation, or disease activity. Accordingly, the potential role of hyperforin in MS remains investigational [138,139,140,141].

#### 5.6.3. Amyotrophic Lateral Sclerosis

Amyotrophic lateral sclerosis (ALS) is characterized by progressive motor-neuron degeneration involving oxidative stress, mitochondrial dysfunction, impaired protein homeostasis, excitotoxicity, and neuroinflammation [142]. Direct evidence evaluating hyperforin in ALS-specific models is very limited. Its antioxidant, mitochondrial, autophagic, and anti-inflammatory actions provide a theoretical basis for neuroprotection [5,14], but these mechanisms are not specific to ALS.

At present, there is insufficient evidence that hyperforin preserves motor neurons, modifies ALS-associated pathological processes, or alters disease progression. Its relevance to ALS should therefore be considered hypothesis-generating rather than evidence of therapeutic efficacy.

### 5.7. Critical Appraisal of Current Evidence

Overall, the evidence supports a biologically plausible neuropsychiatric and neuroprotective profile for hyperforin, but its maturity varies considerably across indications. Depression represents the most developed area, with consistent preclinical findings and complementary clinical evidence from hyperforin-containing *H. perforatum* preparations. Anxiety and cognitive dysfunction have encouraging but less developed evidence, whereas AD and PD remain predominantly preclinical. Evidence for multiple sclerosis is more limited, while HD and ALS remain largely supported by mechanistic extrapolation.

A major limitation is the heterogeneity of the interventions evaluated. Preclinical studies have used both purified hyperforin and hyperforin-containing preparations, whereas human studies have predominantly investigated multicomponent *H. perforatum* products. Consequently, clinical effects cannot be attributed unequivocally to hyperforin. Differences in experimental models, doses, treatment duration, and outcome measures further complicate comparison and translation of preclinical findings. A further limitation is that the exposure conditions producing neuroplastic, neuroprotective, anti-inflammatory, or mitochondrial effects in experimental systems have not consistently been related to concentrations achievable in human plasma or target tissues. Hence, demonstration of TRPC6/BDNF signaling, antioxidant responses, or other neuroprotective mechanisms under experimental conditions should be interpreted as evidence of biological plausibility unless comparable target engagement can be demonstrated at pharmacologically realistic exposure.

Thus, the current literature supports an evidence hierarchy rather than a uniform neurotherapeutic effect of hyperforin. Future research should prioritize chemically characterized purified hyperforin, clinically relevant disease models, dose–exposure characterization, biomarkers of target engagement, and ultimately compound-specific clinical trials to determine whether the promising experimental findings translate into clinically meaningful effects.

The most relevant preclinical findings discussed in Section 5 are summarized in Table 2, highlighting the experimental models, intervention type, principal mechanisms, and major biological outcomes across neuropsychiatric and neurodegenerative conditions.

## 6. Immunomodulatory and Autoimmune Disease Applications

### 6.1. Biological Rationale and Mechanistic Overview

Chronic inflammation and immune dysregulation contribute to the pathogenesis of numerous autoimmune and chronic inflammatory disorders [143,144,145]. Within this context, preclinical evidence suggests that purified hyperforin and hyperforin-containing preparations may exert immunomodulatory effects through interconnected mechanisms involving NF-κB and MAPK signaling, redox homeostasis, intracellular Ca^2+^ regulation, inflammatory cytokine production, and immune-cell function [5,14,63,120,133].

Rather than acting through a single inflammatory target, hyperforin appears to influence both innate and adaptive immune responses through coordinated modulation of these signaling networks. Accordingly, the following subsections focus on the available evidence concerning specific immune-cell populations and disease-relevant immunological outcomes, rather than re-describing the general molecular mechanisms presented in Section 4.

### 6.2. Regulation of Innate Immunity

#### 6.2.1. Macrophages

Experimental studies indicate that purified hyperforin and hyperforin-containing preparations suppress macrophage inflammatory activity, including TNF-α, IL-1β, IL-6, NO, and PGE_2_ production, in association with modulation of NF-κB and MAPK signaling [61,94,120]. Hyperforin-related interventions have also been associated with attenuation of pro-inflammatory M1-like responses and promotion of M2-like reparative phenotypes [120,146]. These findings support macrophage modulation as a potential contributor to the anti-inflammatory effects of hyperforin, although reproducible phenotypic reprogramming across experimental models and its role as a primary mechanism remain insufficiently established.

#### 6.2.2. Neutrophils

Preclinical studies suggest that hyperforin-related interventions may attenuate neutrophil-mediated inflammation by reducing activation and oxidative burst, release of elastase and myeloperoxidase, and neutrophil recruitment, chemotaxis, adhesion, and transendothelial migration [63,66,120,147,148]. These findings support a potential role in limiting excessive neutrophil-driven inflammatory responses; however, the evidence remains predominantly experimental, and effects on neutrophil extracellular trap formation and disease-specific outcomes remain insufficiently characterized.

#### 6.2.3. Dendritic Cells

Experimental evidence suggests that hyperforin may influence dendritic-cell activation and antigen-presenting function. Hyperforin-related interventions have been associated with reduced expression of co-stimulatory molecules and decreased production of pro-inflammatory cytokines, potentially limiting excessive activation of adaptive immune responses [94,149].

However, evidence concerning direct effects of hyperforin on dendritic-cell differentiation, maturation, antigen presentation, and induction of stable tolerogenic phenotypes remains limited. Thus, dendritic-cell modulation should currently be regarded as a potential component of hyperforin-mediated immunoregulation rather than an established primary mechanism.

### 6.3. Regulation of Adaptive Immunity

#### 6.3.1. T-Cell Responses

Preclinical evidence indicates that purified hyperforin and hyperforin-containing preparations may modulate T-cell activation, proliferation, differentiation, and cytokine production through signaling networks involving NF-κB, STAT, MAPK, and Ca^2+^-dependent pathways [94,139,140]. In experimental models, hyperforin-related interventions have been associated with attenuation of pro-inflammatory Th1- and Th17-related responses and reduced production of cytokines including interferon-γ (IFN-γ), TNF-α, and IL-17 [94,139,140].

These findings support T-cell modulation as a potentially relevant component of the immunoregulatory effects of hyperforin. However, evidence concerning regulatory T-cell (Treg) responses and the induction of durable immune tolerance remains limited, and the extent to which these effects are directly attributable to hyperforin rather than broader changes in inflammatory signaling requires further clarification.

#### 6.3.2. B-Cell Responses

Evidence concerning the effects of hyperforin on B-cell function is substantially more limited than that for T cells. Available experimental findings suggest that hyperforin-related interventions may influence B-cell responses indirectly through modulation of inflammatory signaling and T-cell–B-cell interactions [139,140,149]. However, direct effects on B-cell activation and differentiation, plasma-cell function, autoantibody production, and antibody-mediated immune responses remain insufficiently characterized [139,140,149].

Accordingly, B-cell regulation should currently be considered a preliminary and incompletely defined component of hyperforin-mediated immunomodulation, rather than an established mechanism of action.

### 6.4. Autoimmune and Immune-Mediated Disease Applications

#### 6.4.1. Rheumatoid Arthritis

The immunomodulatory actions of hyperforin provide a rationale for its investigation in rheumatoid arthritis (RA). Experimental evidence indicates suppression of inflammatory cytokine production, NF-κB-associated signaling, oxidative stress, and immune-cell activation [61,63,140,146,149,150]. However, it remains unclear whether these effects translate into sustained protection against synovitis or structural joint damage in robust RA-specific models. Accordingly, the therapeutic relevance of hyperforin in RA remains preliminary and requires further disease-specific preclinical validation.

#### 6.4.2. Inflammatory Bowel Disease

In inflammatory bowel disease (IBD), experimental evidence suggests that hyperforin may attenuate inflammatory signaling and cytokine production while supporting epithelial barrier integrity [14,63,150,151]. These findings provide a mechanistic rationale for potential relevance to intestinal inflammation; however, evidence from experimental colitis and mucosal-barrier models is discussed in detail in Section 10. Clinical efficacy in patients with IBD has not been established.

#### 6.4.3. Additional Autoimmune and Immune-Mediated Disorders

The immunomodulatory properties of hyperforin have also prompted investigation in other autoimmune and inflammatory conditions involving dysregulated immune signaling and oxidative stress [152]. However, for most of these disorders, therapeutic relevance is inferred predominantly from shared mechanisms rather than disease-specific evidence.

Among the comparatively better-characterized applications, experimental studies indicate that hyperforin may attenuate psoriasis-like inflammation and modulate keratinocyte proliferation and differentiation [153,154,155]. Hyperforin and *H. perforatum* preparations have also shown protective effects against cytokine-associated inflammatory and oxidative injury in pancreatic β-cell models, providing preliminary relevance to type 1 diabetes mellitus [156,157]. Nevertheless, these findings remain preclinical and do not establish clinical efficacy in either condition.

For several additional autoimmune and immune-mediated disorders, including systemic lupus erythematosus, autoimmune thyroid disease, Sjögren syndrome, systemic sclerosis, autoimmune uveitis, autoimmune hepatitis, vitiligo, and alopecia areata, direct disease-specific evidence remains sparse. Their proposed relevance to hyperforin is based predominantly on extrapolation from the anti-inflammatory, redox-regulatory, immunomodulatory, antifibrotic, or tissue-protective mechanisms described in Section 4 rather than on dedicated disease-specific studies [158,159,160,161]. These indications should therefore be considered exploratory and are not discussed mechanistically in further detail.

### 6.5. Critical Appraisal of Current Evidence

Overall, the available evidence supports a biologically plausible immunomodulatory profile for hyperforin but remains predominantly mechanistic and preclinical. The strongest experimental support concerns modulation of macrophage and T-cell responses, including attenuation of pro-inflammatory signaling and Th1/Th17-associated activity. Evidence for neutrophil and dendritic-cell regulation is more limited, whereas direct effects on B-cell differentiation, plasma-cell function, autoantibody production, and durable immune tolerance remain insufficiently characterized. Thus, the evidence supports modulation of selected immune pathways rather than broad or established immunoregulatory efficacy.

The maturity of evidence also differs substantially among disease applications. Psoriasis and selected inflammatory models have comparatively more disease-specific experimental support, whereas rheumatoid arthritis remains insufficiently validated in robust disease-specific models despite a plausible inflammatory rationale. Evidence for several other autoimmune conditions—including systemic lupus erythematosus, autoimmune thyroid disease, Sjögren’s syndrome, and systemic sclerosis—is largely extrapolated from shared anti-inflammatory, antioxidant, or immunomodulatory mechanisms and should therefore be considered exploratory.

Interpretation is further complicated by heterogeneity in experimental models and interventions, including the use of both purified hyperforin and multicomponent *H. perforatum* preparations. Effects observed with botanical preparations cannot be attributed unequivocally to hyperforin, and clinical trials of purified hyperforin in autoimmune or systemic immune-mediated diseases are lacking. Accordingly, current evidence demonstrates preclinical immunomodulatory potential rather than established therapeutic efficacy. Moreover, the concentrations required to modulate NF-κB-, cytokine-, and immune-cell signaling in many experimental systems have not been systematically related to achievable human exposure; these findings should therefore be regarded as mechanistic evidence until exposure-compatible target engagement is demonstrated. Future research should prioritize chemically defined interventions, disease-specific models, dose–response characterization, and translational studies capable of determining whether these immunological effects can produce clinically meaningful disease modification.

The most relevant preclinical findings discussed in Section 6 are summarized in Table 3, highlighting the immune-cell targets, experimental context, intervention type, principal mechanisms, and major immunomodulatory outcomes of hyperforin-related interventions.

## 7. Cardiovascular and Metabolic Health Effects

### 7.1. Biological Rationale

Cardiovascular and metabolic disorders involve interconnected inflammatory, redox, endothelial, metabolic, and mitochondrial disturbances [162]. Given the ability of hyperforin to modulate several of these processes, as described in Section 4, increasing attention has focused on its potential cardiometabolic effects.

Experimental evidence suggests that purified hyperforin and hyperforin-containing preparations may influence vascular function, lipid and adipose-tissue metabolism, glucose homeostasis, and insulin sensitivity [1,163]. However, the evidence remains predominantly mechanistic and preclinical, with comparatively limited direct animal and human data on clinically relevant cardiovascular and metabolic outcomes. Accordingly, the following subsections focus on disease- and tissue-specific evidence rather than reiterating the molecular mechanisms described in Section 4.

### 7.2. Endothelial and Vascular Function

Experimental evidence suggests that purified hyperforin and hyperforin-containing preparations may protect endothelial and vascular cells by attenuating oxidative and inflammatory signaling, preserving eNOS/NO-related function, reducing NF-κB-associated endothelial activation and adhesion-molecule expression, and supporting mitochondrial integrity [63,120,150,160,164,165,166]. However, these effects primarily represent shared cytoprotective mechanisms, and direct evidence for improved vasodilation, vascular remodeling, or clinically relevant cardiovascular outcomes remains limited.

### 7.3. Potential Anti-Atherosclerotic Effects

#### 7.3.1. Inflammation and Foam-Cell Formation

Atherosclerosis is driven by lipid accumulation, vascular inflammation, oxidative stress, and macrophage-derived foam-cell formation [167]. Hyperforin-related interventions may attenuate pro-atherogenic responses through suppression of NF-κB-associated inflammation and modulation of macrophage activation, lipid handling, and foam-cell formation [61,63,120,164,166,168,169]. However, disease-specific in vivo evidence remains limited, and these findings do not yet establish anti-atherosclerotic efficacy.

#### 7.3.2. Oxidative Stress

Oxidative stress contributes to atherogenesis by promoting endothelial dysfunction, lipid oxidation, inflammatory activation, and vascular injury [164,167]. Experimental studies indicate that hyperforin-related interventions may reduce reactive oxygen species accumulation and enhance endogenous antioxidant defenses, including enzymatic and glutathione-dependent systems [65,66,156,170]. These effects may limit oxidative modification of vascular lipids and other cellular components implicated in atherosclerotic progression.

Nevertheless, redox modulation represents a broad cytoprotective property of hyperforin rather than an atherosclerosis-specific mechanism. Evidence directly linking these antioxidant effects to reduced LDL oxidation, plaque development, or progression of established atherosclerosis remains insufficient. Accordingly, current findings support mechanistic anti-atherogenic plausibility rather than established anti-atherosclerotic efficacy.

### 7.4. Lipid Metabolism

#### 7.4.1. Cholesterol Homeostasis

Experimental evidence suggests that purified hyperforin and hyperforin-containing preparations may influence cholesterol homeostasis through modulation of inflammatory and metabolic signaling pathways [63,160,164,170,171] and nuclear receptors and transcriptional regulators involved in lipid synthesis, transport, and cellular handling [172,173,174]. Hyperforin-related interventions may also attenuate oxidative modification of LDL particles, potentially reducing their pro-inflammatory and atherogenic properties [175,176].

However, direct evidence that hyperforin produces sustained or clinically meaningful changes in circulating LDL-C, HDL-C, or total cholesterol remains insufficient. Its effects on cholesterol metabolism should therefore be considered predominantly mechanistic and preclinical.

#### 7.4.2. Triglyceride and Fatty-Acid Metabolism

In vitro and in vivo studies suggest that purified hyperforin and hyperforin-containing preparations may influence triglyceride and fatty-acid metabolism through modulation of mitochondrial function, energy-sensing pathways, fatty-acid utilization, and lipid storage [96,160,170]. These effects may favor fatty-acid oxidation and limit intracellular lipid accumulation under selected experimental conditions.

Nevertheless, evidence demonstrating sustained reductions in circulating triglycerides or clinically meaningful improvements in lipid metabolism remains limited. Further compound-specific studies are required to establish the translational relevance of these findings.

### 7.5. Obesity and Adipose Tissue Biology

#### 7.5.1. Adipogenesis

Adipose tissue expansion in obesity involves dysregulated adipogenesis accompanied by inflammatory, oxidative, and metabolic disturbances [177,178]. Experimental studies indicate that hyperforin-containing preparations may modulate adipocyte differentiation through regulation of key adipogenic transcription factors, including peroxisome proliferator-activated receptor γ (PPARγ) and CCAAT/enhancer-binding proteins (C/EBPα and C/EBPβ) [179,180,181,182,183]. Changes in downstream adipogenic markers, including adipocyte protein 2/fatty acid-binding protein 4 (aP2/FABP4), adiponectin, and lipoprotein lipase, further support effects on adipocyte differentiation and lipid-storage programs [180,183].

These findings suggest that hyperforin-related interventions may influence adipose-tissue expansion by modulating adipogenic signaling. However, much of the available evidence derives from *H. perforatum* preparations rather than purified hyperforin, limiting compound-specific attribution and requiring confirmation in physiologically relevant obesity models.

#### 7.5.2. Energy Metabolism

Experimental evidence suggests that hyperforin-related interventions may influence cellular energy metabolism through modulation of AMPK, mitochondrial function, oxidative phosphorylation, and pathways associated with mitochondrial biogenesis [66,67,84,96,170,184,185]. These effects may promote glucose utilization and fatty-acid oxidation while limiting excessive lipid storage under selected experimental conditions.

However, evidence that these cellular and molecular effects translate into sustained increases in whole-body energy expenditure, reduced adiposity, or clinically meaningful improvements in energy balance remains limited. Thus, the metabolic effects of hyperforin should currently be interpreted primarily as preclinical evidence of altered cellular bioenergetics rather than established anti-obesity activity.

#### 7.5.3. Adipokines

Adipose tissue functions as an endocrine organ that secretes adipokines involved in the regulation of inflammation, insulin sensitivity, appetite, and energy balance. Dysregulated adipokine secretion contributes to obesity-associated metabolic and cardiovascular complications [186]. Purified hyperforin and hyperforin-containing preparations may modulate adipokine profiles, including increased adiponectin and reduced pro-inflammatory mediators, through anti-inflammatory and metabolic mechanisms [170,179,182,183]. These changes may support insulin sensitivity, although evidence for sustained improvements in adipose-tissue or systemic metabolic function remains insufficient.

### 7.6. Type 2 Diabetes, Glucose Homeostasis, and Insulin Signaling

Type 2 diabetes mellitus (T2DM) is characterized by chronic hyperglycemia arising from insulin resistance, impaired β-cell function, and dysregulated glucose metabolism, with inflammation, oxidative stress, mitochondrial dysfunction, and altered cellular energy metabolism contributing to its pathogenesis [187].

In vitro and animal studies suggest that purified hyperforin and hyperforin-containing preparations may influence glucose homeostasis and insulin-related responses through interconnected inflammatory, redox, mitochondrial, and AMPK-associated pathways [84,96,170,181,182,188]. Suppression of TNF-α and IL-6 and protection of pancreatic β-cells and insulin-responsive tissues against oxidative injury may further contribute to metabolic effects [65,84,156,164,165,170,188]. However, direct evidence for sustained improvement in insulin sensitivity or glycemic control remains limited, particularly for purified hyperforin.

### 7.7. Critical Appraisal of the Current Evidence

Overall, the available evidence supports a biologically plausible cardiometabolic profile for hyperforin, but remains predominantly mechanistic and preclinical. The comparatively stronger evidence concerns obesity-associated metabolic dysfunction, adipose-tissue inflammation, insulin resistance, and glucose homeostasis. In contrast, evidence for endothelial dysfunction, atherosclerosis, hypertension, and clinically relevant cardiovascular outcomes is substantially less developed and is based largely on mechanistic observations or limited disease-specific models. Direct evidence demonstrating sustained improvements in vascular function, atherosclerotic burden, or glycemic control remains insufficient.

Interpretation is further limited by substantial heterogeneity in experimental models, doses, exposure periods, formulations, and measured outcomes. Studies have also employed both purified hyperforin and hyperforin-containing *H. perforatum* preparations, preventing unequivocal attribution of findings from botanical preparations to hyperforin itself. This distinction is particularly important because several vascular and metabolic effects are inferred from general anti-inflammatory, antioxidant, mitochondrial, or metabolic actions rather than demonstrated through robust compound-specific disease models. Consequently, metabolic and vascular pathway modulation demonstrated experimentally should not be assumed to represent clinically achievable target engagement unless supported by exposure–response data compatible with human pharmacokinetics.

Accordingly, the current literature supports preclinical cardiometabolic potential rather than established therapeutic efficacy. Future research should prioritize chemically characterized purified hyperforin, pharmacologically realistic exposure levels, robust disease-specific animal models, and direct assessment of clinically relevant outcomes such as insulin sensitivity, glycemic control, vascular function, and atherosclerotic progression. Subsequent human pharmacokinetic and controlled clinical studies will be required to determine whether these experimental effects can translate into meaningful cardiometabolic benefit.

The most relevant preclinical findings discussed in Section 7 are summarized in Table 4, with emphasis on the experimental context, intervention type, principal cardiometabolic mechanisms, and major biological outcomes reported for hyperforin-related interventions.

## 8. Anticancer Properties

### 8.1. Biological Rationale

Cancer is characterized by the acquisition of biological capabilities that promote uncontrolled proliferation, resistance to cell death, angiogenesis, metabolic reprogramming, immune evasion, invasion, metastasis, and therapeutic resistance [189]. Despite major advances in precision oncology, targeted therapies, and immunotherapy, tumor heterogeneity, treatment resistance, toxicity, and recurrence remain important clinical challenges [190].

Experimental studies suggest that purified hyperforin and hyperforin-containing preparations may influence several cancer-associated processes, including cell-cycle progression, apoptosis, autophagy, inflammation, angiogenesis, invasion, metastasis, and tumor–microenvironment interactions [190]. However, the available evidence is derived predominantly from in vitro studies, with comparatively limited in vivo validation. Accordingly, the following subsections focus on the principal experimentally supported anticancer mechanisms and tumor-specific findings rather than repeatedly describing the broader molecular pathways already discussed in Section 4.

### 8.2. Overview of Anticancer Mechanisms

#### 8.2.1. Cell Cycle Arrest

Dysregulated cell-cycle control is a central feature of malignant transformation [191]. In vitro studies indicate that purified hyperforin and hyperforin-containing preparations can inhibit cancer-cell proliferation through modulation of cell-cycle regulators [70,73,192]. Reported effects include reduced expression or activity of cyclins D1, E, and A and cyclin-dependent kinase 2 (CDK2), CDK4, and CDK6, together with increased expression of the cyclin-dependent kinase inhibitors p21^Cip1/Waf1^ and p27^Kip1^ [70,73,192].

These changes are accompanied in several cancer models by inhibition of proliferative and survival pathways, including PI3K/Akt, ERK/MAPK, STAT3, and NF-κB. Collectively, these findings support cell-cycle disruption as an important component of the antiproliferative activity of hyperforin, although the specific response appears to vary according to tumor type and experimental context.

#### 8.2.2. Apoptosis Induction

Apoptosis induction represents one of the most consistently reported anticancer actions of hyperforin. In vitro studies indicate that purified hyperforin and hyperforin-containing preparations can activate both intrinsic and extrinsic apoptotic pathways [69,73,75]. Reported effects include mitochondrial membrane depolarization, cytochrome-c release, activation of caspase-9 and caspase-3, and modulation of Bcl-2 family proteins [69,73,75,76,193,194].

Hyperforin has also been reported to reduce the expression of anti-apoptotic proteins such as Bcl-2 and Bcl-xL while increasing pro-apoptotic mediators including Bax and Bak [69,73,74,75]. These findings provide a coherent mechanistic basis for the cytotoxic effects observed in several malignant cell types. However, much of this evidence remains restricted to in vitro studies, and several investigations have employed hyperforin-containing preparations rather than purified hyperforin. Therefore, confirmation under clinically relevant exposure conditions and in robust in vivo models remains necessary.

#### 8.2.3. Autophagy Regulation

Autophagy has context-dependent functions in cancer, acting either as a tumor-suppressive mechanism or as an adaptive survival response [195]. Preliminary in vitro evidence suggests that purified hyperforin and hyperforin-containing preparations may modulate autophagic pathways through PI3K/Akt/mammalian target of rapamycin (PI3K/Akt/mTOR)- and AMPK-related signaling [74,75,76].

Depending on tumor type and cellular context, these effects may interact with apoptotic signaling and contribute to growth inhibition or altered sensitivity to anticancer treatments [74,75,76]. However, the role of autophagy in hyperforin-mediated anticancer activity remains incompletely defined and requires additional studies, particularly in vivo and using purified hyperforin.

### 8.3. Breast Cancer

Breast cancer is among the better-investigated solid malignancies for hyperforin. Purified hyperforin and hyperforin-containing preparations have demonstrated antiproliferative and pro-apoptotic effects involving NF-κB, PI3K/Akt, estrogen-responsive, ERK/MAPK, and STAT3 signaling [73,74,76,192,196], together with effects on migration, invasion, EMT, and stemness-associated pathways [74,75,192,197]. In MCF-7, MDA-MB-468, and other breast cancer models, these interventions have shown antiproliferative, pro-apoptotic, and anti-invasive activity [70,73,192,198], while limited in vivo studies suggest reduced tumor growth, angiogenesis, and metastatic dissemination [73,192,199]. Although some studies indicate preferential cytotoxicity toward malignant over non-transformed cells [70,73,200,201], evidence for EMT/cancer-stem-cell modulation and tumor selectivity remains limited. Thus, despite relatively well-developed preclinical evidence, the clinical relevance and therapeutic index of hyperforin in breast cancer remain unestablished.

### 8.4. Colorectal Cancer

Experimental studies indicate that purified hyperforin and hyperforin-containing preparations inhibit colorectal cancer cell proliferation, viability, clonogenic survival, migration, and invasion while promoting apoptosis [69,73,75,76,202,203]. These effects have been associated with suppression of JAK1/STAT3, PI3K/Akt, ERK/MAPK, and NF-κB signaling [67,69,73,75,76,202,203] and activation of extrinsic and intrinsic apoptotic pathways involving Fas/FasL, mitochondrial dysfunction, caspase activation, PARP cleavage, cell-cycle perturbation, and downregulation of pro-survival proteins including c-FLIP, XIAP, and MCL-1 [69,73,75,76,202,203].

Modulation of Wnt/β-catenin signaling and associated cancer-stem-cell features, including self-renewal and expression of LGR5 and CD133, has also been reported in experimental cancer systems [67,73,76]. However, direct evidence for robust Wnt/β-catenin inhibition specifically in colorectal cancer remains limited. Overall, the available evidence supports antiproliferative and pro-apoptotic activity of hyperforin in colorectal cancer models, but several proposed mechanisms require further disease-specific validation.

### 8.5. Prostate Cancer

In vitro studies in androgen-dependent LNCaP and androgen-independent PC-3 and DU145 prostate cancer cells demonstrate that purified hyperforin suppresses proliferation and DNA synthesis and induces caspase-dependent apoptosis [70]. These effects involve inhibition of PI3K/Akt signaling, downregulation of anti-apoptotic Bcl-2, MCL-1, and XIAP, mitochondrial cytochrome-c release, caspase-9/-3 activation [69,70,192], and increased expression of pro-apoptotic BAK, BAD, and NOXA [69,73,75,192]. NF-κB, STAT3, and MAPK/ERK signaling may also contribute, although prostate-cancer-specific evidence remains limited [69,70,192]. Overall, hyperforin shows antiproliferative and pro-apoptotic activity in prostate cancer cells, but the predominantly in vitro evidence requires robust in vivo validation before clinical translation.

### 8.6. Leukemia and Hematological Malignancies

Purified hyperforin has demonstrated antiproliferative and pro-apoptotic activity in acute myeloid leukemia, chronic lymphocytic leukemia, and other leukemic models through mitochondrial/caspase-dependent mechanisms and suppression of Akt-, NF-κB-, and STAT-associated survival pathways [71,192,204,205]. Some studies suggest greater susceptibility of malignant than normal hematopoietic cells [71,192,204,206]; however, evidence remains predominantly in vitro and does not yet define an in vivo therapeutic window.

### 8.7. Effects on the Tumor Microenvironment

#### 8.7.1. Inflammatory Signaling

Chronic inflammatory signaling within the tumor microenvironment (TME) contributes to tumor-cell survival, angiogenesis, invasion, metastatic progression, and immune evasion [207,208,209]. Experimental evidence indicates that mainly purified hyperforin, and to a lesser extent hyperforin-containing preparations, can suppress NF-κB-associated signaling and reduce inflammatory mediators including TNF-α, IL-1β, IL-6, PGE_2_, and COX-2 [62,66,75,76,203,206,207].

These effects may alter tumor–stromal and tumor–immune interactions; however, direct evidence demonstrating functional reprogramming of the TME in vivo remains limited. Therefore, the TME-related consequences of hyperforin’s anti-inflammatory activity remain plausible but incompletely established.

#### 8.7.2. Angiogenesis

Experimental studies suggest that purified hyperforin can inhibit several endothelial responses involved in tumor angiogenesis, including endothelial-cell proliferation, migration, capillary-tube formation, tumor vascularization, and lymphangiogenesis [199,203,210,211,212]. These effects have been associated partly with inhibition of NF-κB-dependent responses to vascular endothelial growth factor (VEGF) and other pro-angiogenic stimuli.

A mechanistic study further demonstrated inhibition of endothelial responses to multiple angiogenic growth factors and cytokines, including VEGF-related pathways [211]. Nevertheless, the effects of hyperforin on VEGF signaling appear context-dependent. In non-cancer settings, such as post-stroke recovery, hyperforin has been reported to increase VEGF expression and promote reparative angiogenesis [213,214].

Accordingly, hyperforin should not simply be characterized as an antiangiogenic compound. Its effects on vascular signaling appear to depend on tissue context and disease state, and further mechanistic and in vivo studies are required.

#### 8.7.3. Immune Modulation

In vitro evidence suggests that purified hyperforin and hyperforin-containing preparations may modulate immune-related signaling relevant to the TME through effects on inflammatory cytokines and adaptive immune responses [66,153,205]. Reported findings include reductions in TNF-α, IL-1β, IL-6, IL-17A, and interferon (IFN)-γ, inhibition of NF-κB- and STAT3-dependent signaling, and modulation of Th1- and Th17-associated responses [66,153,205].

However, direct tumor-specific evidence concerning macrophage polarization, dendritic-cell activity, regulatory immune phenotypes, and antitumor immune surveillance remains limited. Therefore, the proposal that hyperforin “reprograms” the TME toward a less immunosuppressive phenotype is currently stronger than the available evidence supports. Its immunomodulatory role in cancer should instead be considered hypothesis-generating and requires direct validation in immunocompetent tumor models.

### 8.8. Combination with Conventional Anticancer Therapies

#### 8.8.1. Chemotherapy

In vitro studies suggest that purified hyperforin and hyperforin-containing preparations may enhance the activity of selected anticancer agents through sensitization of malignant cells to apoptosis and suppression of survival pathways such as PI3K/Akt, NF-κB, and STAT3 [67,70,192,206].

These findings provide a rationale for potential pharmacodynamic synergy; however, in vivo and clinical combination data remain limited, and the potential for pharmacokinetic drug–herb interactions requires particular caution [67,70,192,206].

Consequently, the possibility that hyperforin could reduce required chemotherapy doses or treatment toxicity remains speculative and should not be inferred from in vitro sensitization studies alone.

#### 8.8.2. Targeted Therapy

Hyperforin’s ability to influence PI3K/Akt, NF-κB, STAT3, MAPK/ERK, Wnt/β-catenin, and related survival networks provides a theoretical basis for combination with targeted therapies [67,70,206]. However, direct evidence involving clinically used epidermal growth factor receptor (EGFR), anaplastic lymphoma kinase (ALK), PARP, CDK4/6, or related targeted agents remains scarce.

Thus, although the multitarget actions of hyperforin may theoretically influence resistance pathways, this concept remains primarily mechanistic and should not be presented as demonstrated therapeutic synergy.

#### 8.8.3. Immunotherapy

The immunomodulatory effects of hyperforin provide a theoretical rationale for investigation alongside cancer immunotherapies [149,153,215]. However, direct studies evaluating combinations with immune-checkpoint inhibitors or other contemporary immunotherapies remain very limited.

Although modulation of inflammatory signaling, cytokine production, and immune-cell activity may potentially influence tumor–immune interactions [149,153,215], there is currently insufficient evidence to conclude that hyperforin enhances immunotherapeutic efficacy. Dedicated immunocompetent animal studies should therefore precede any consideration of clinical combination strategies.

### 8.9. Critical Appraisal of Current Evidence

Overall, the available evidence supports substantial experimental anticancer activity of hyperforin, particularly through antiproliferative, pro-apoptotic, anti-invasive, and antiangiogenic mechanisms. The evidence is comparatively more developed in breast, colorectal, prostate, and hematological cancer models. However, most studies remain in vitro, while robust in vivo validation using orthotopic, patient-derived, genetically engineered, or other clinically representative tumor models is limited. Therefore, the current literature supports biological plausibility and preclinical anticancer activity rather than established therapeutic efficacy.

Interpretation is further constrained by substantial heterogeneity in cancer type, experimental model, formulation, dose, exposure conditions, and outcome measures. Importantly, some in vitro studies have used purified hyperforin at concentrations that may exceed or cannot yet be confidently related to clinically achievable systemic or tumor-tissue exposure; effects demonstrated under such conditions should therefore be interpreted as evidence of anticancer biological activity rather than clinically achievable target engagement. In addition, both purified hyperforin and hyperforin-containing *H. perforatum* preparations have been investigated, so findings obtained with botanical preparations cannot be attributed unequivocally to hyperforin itself.

A further translational concern involves combination therapy. Although experimental studies suggest potential sensitization to chemotherapy and modulation of pathways relevant to targeted therapy or immunotherapy, these findings remain preliminary. Hyperforin also affects drug-metabolizing enzymes and transporters, raising the possibility that pharmacokinetic interactions could counteract or complicate any pharmacodynamic synergy. Accordingly, future studies should prioritize chemically characterized purified hyperforin, pharmacologically realistic exposure levels, robust in vivo tumor models, tumor-versus-normal tissue selectivity, and formal PK/PD and interaction studies before compound-specific clinical anticancer trials are pursued.

The most relevant preclinical anticancer findings discussed in Section 8 are summarized in Table 5, with emphasis on tumor model, intervention type, principal molecular mechanisms, and major experimentally observed outcomes.

## 9. Antimicrobial and Antiviral Activities

### 9.1. Biological Rationale

The continuing burden of infectious diseases, antimicrobial resistance (AMR), and emerging or re-emerging viral pathogens has intensified the search for anti-infective agents with alternative or multitargeted mechanisms [216,217]. Natural products remain an important source of antimicrobial compounds, including phytochemicals with direct antimicrobial and host-modulatory properties [13,218].

Among the bioactive constituents of *H. perforatum*, hyperforin has demonstrated antibacterial, antifungal, and antiviral activity against several clinically relevant pathogens [219]. Proposed mechanisms include disruption of microbial membrane integrity and ion homeostasis, interference with energy metabolism, inhibition of biofilm formation and virulence-associated processes, and modulation of host immune responses [219]. The following subsections therefore focus on pathogen-specific evidence and the relative strength of support across antibacterial, antifungal, antiviral, and antimicrobial-resistance-related applications.

### 9.2. Antibacterial Effects

#### 9.2.1. Gram-Positive Bacteria

The antibacterial activity of purified hyperforin and hyperforin-containing *H. perforatum* preparations is most consistently demonstrated against Gram-positive bacteria. In vitro studies have reported activity against several clinically relevant species, including Staphylococcus aureus, methicillin-resistant *Staphylococcus aureus* (MRSA), *Streptococcus pneumoniae*, *Streptococcus pyogenes*, *Enterococcus faecalis*, and *Bacillus subtilis*, with low minimum inhibitory concentrations reported for several organisms and particularly pronounced activity against resistant staphylococcal strains [220,221,222,223,224,225,226,227,228,229]. However, these findings remain predominantly in vitro and have not been extensively validated against standard antibiotics in relevant animal infection models.

Mechanistically, hyperforin appears to disrupt bacterial membrane integrity and transmembrane electrochemical gradients, thereby impairing permeability, ion homeostasis, proton motive force, ATP generation, and cellular energy metabolism [66,220,229,230]. Hyperforin and related derivatives have also inhibited biofilm-associated bacterial growth and disrupted established biofilms, potentially increasing microbial susceptibility to antimicrobial agents [228,229]. Nevertheless, the effects on specific stages of biofilm development and their therapeutic relevance require further characterization.

#### 9.2.2. Gram-Negative Bacteria

Gram-negative bacteria generally show lower susceptibility to hyperforin than Gram-positive organisms, partly because the outer membrane restricts penetration of highly lipophilic compounds [220,229,231]. Nevertheless, antibacterial activity has been reported against selected Gram-negative pathogens, including *Escherichia coli*, *Pseudomonas aeruginosa*, *Klebsiella pneumoniae*, *Salmonella enterica*, and *Helicobacter pylori*, although these effects are generally weaker and may be enhanced in combination with conventional antibiotics [223,226,232,233,234].

Notably, purified hyperforin has been shown to increase membrane permeability and potentiate polymyxin B activity against multidrug-resistant Gram-negative bacteria, supporting its potential use as an antimicrobial adjuvant [229]. Hyperforin-related anti-inflammatory and cytoprotective effects may additionally attenuate infection-associated tissue injury [66]; however, these host-directed actions should be distinguished from direct antibacterial activity. Overall, current evidence supports stronger direct antibacterial activity against Gram-positive organisms, whereas the potential relevance to Gram-negative infections may lie primarily in combination or adjuvant strategies.

### 9.3. Antifungal Activity

Fungal infections remain an important clinical challenge, particularly in immunocompromised populations, with increasing antifungal resistance reinforcing the need for compounds with alternative mechanisms of action [235,236]. In vitro studies have demonstrated antifungal activity of hyperforin-containing preparations and, to a lesser extent, purified hyperforin or related compounds against *Candida*, *Cryptococcus*, *Trichophyton*, and *Microsporum* spp., with more limited evidence for *Aspergillus* spp. [237,238].

Proposed mechanisms include disruption of fungal membrane integrity and transmembrane ion gradients, with consequent impairment of cellular homeostasis, bioenergetics, and growth-associated signaling [84,239,240,241,242]. Hyperforin-containing preparations may also inhibit fungal biofilm formation and influence virulence-associated processes, although direct evidence for specific virulence effects remains limited [239,240].

Overall, the antifungal evidence is considerably less developed than the antibacterial evidence and remains predominantly in vitro. Moreover, most studies have evaluated hyperforin-containing preparations rather than purified hyperforin, preventing unequivocal attribution of the observed effects to hyperforin. Further studies using purified hyperforin and clinically relevant in vivo infection models are therefore required to establish compound-specific antifungal activity and translational relevance.

### 9.4. Antiviral Activity

Viruses remain major contributors to global morbidity and mortality, and the continuing emergence of novel viral pathogens reinforces the need for new antiviral strategies [243]. Experimental evidence suggests that purified hyperforin and hyperforin-containing preparations may exert antiviral activity, although the strength of evidence and the contribution of hyperforin vary considerably among viral species.

#### 9.4.1. Enveloped Viruses

The strongest preclinical antiviral evidence concerns enveloped viruses. In vitro studies have reported activity of hyperforin-containing preparations and, to a lesser extent, purified hyperforin against herpes simplex virus (HSV), human immunodeficiency virus (HIV), and influenza viruses [244,245,246,247,248,249]. Evidence for hepatitis C virus is more limited and derives predominantly from *H. perforatum* extracts or hypericin, while activity reported against SARS-CoV-2 and other coronaviruses appears to be associated primarily with hypericin and pseudohypericin rather than hyperforin [244,245,246,247,248,249,250].

Hyperforin-related antiviral activity may involve inhibition of intracellular viral replication and propagation, including post-entry processes and viral RNA synthesis [248,249,250,251]. Additional mechanisms proposed for *H. perforatum* preparations include interference with viral infectivity, host–cell pathways required for replication, and possibly envelope-associated processes; however, direct effects on viral attachment, entry, or assembly remain insufficiently established and may vary among viruses and phytochemical constituents [248,251].

The anti-inflammatory and cytoprotective actions of hyperforin may additionally contribute to host protection by modulating cytokine responses, oxidative stress, and immune signaling [66,120,162,170,248,252]. However, direct evidence linking these host-directed effects to improved outcomes during viral infection remains limited, and the proposed combined antiviral–host-modulatory role requires validation.

#### 9.4.2. Non-Enveloped Viruses

Evidence for hyperforin against non-enveloped viruses is substantially more limited, potentially reflecting their lack of a lipid envelope and consequently lower susceptibility to membrane-active compounds [253]. Available studies suggest that hyperforin-containing preparations and, to a lesser extent, purified hyperforin may influence intracellular viral propagation through modulation of host–cell pathways, including NF-κB, MAPK, Ca^2+^-dependent signaling, and endoplasmic-reticulum stress responses [8,66,219,242,248,249,254,255].

However, these mechanistic interactions do not establish direct antiviral activity, and proposed effects involving inflammatory, redox, or metabolic pathways remain largely indirect. Accordingly, the antiviral activity of hyperforin against non-enveloped viruses should currently be considered preliminary and requires direct virus-specific experimental validation.

### 9.5. Potential Role Against Antimicrobial Resistance

Antimicrobial resistance (AMR) represents a major global health threat that increasingly compromises the effectiveness of existing therapies [256,257]. Several properties of hyperforin provide a rationale for investigating its potential role in this context.

Hyperforin acts through multiple antimicrobial mechanisms rather than a single molecular target [83,229,258]. Although such multitarget activity has been proposed to reduce the likelihood of resistance development [83,229,258], this hypothesis requires confirmation in prospective resistance-selection studies. In vitro activity has also been demonstrated against resistant Gram-positive pathogens, MRSA, as well as *Streptococcus epidermidis*, *Streptococcus* spp., and *Enterococcus* spp. [83,229,233,258]. Available evidence further suggests that hyperforin resistance is not necessarily associated with cross-resistance to conventional antibacterial agents [83,229,233,258].

A potentially important application is the use of hyperforin as an antimicrobial adjuvant. Purified hyperforin potentiated polymyxin B activity against multidrug-resistant Pseudomonas aeruginosa, *Klebsiella pneumoniae*, and *Acinetobacter baumannii*, enhancing bacterial killing through membrane perturbation, oxidative stress, and biofilm disruption [229]. Hyperforin and related compounds have also inhibited biofilm formation and disrupted established biofilms, including those formed by MRSA and *Enterococcus faecalis*, while combinations with conventional antibiotics enhanced eradication of multidrug-resistant biofilms [229,259,260].

Hyperforin may additionally attenuate infection-associated inflammatory and oxidative responses through modulation of cytokine, NF-κB-, STAT-, and Ca^2+^-dependent signaling [66,207,219,261]. However, these effects should be regarded as host-directed actions rather than evidence of direct antimicrobial efficacy.

Overall, the most plausible translational role of hyperforin in AMR may lie in adjunctive antibacterial and antibiofilm strategies rather than replacement of established antimicrobial agents. Further studies using purified hyperforin are required to establish compound-specific effects and determine whether the promising in vitro findings translate into clinically relevant antimicrobial activity.

### 9.6. Critical Appraisal for Current Evidence

Overall, the available evidence supports preclinical antimicrobial activity of hyperforin, but the strength of evidence differs substantially across pathogen classes. The most consistent findings concern antibacterial activity against Gram-positive organisms, including resistant strains, with additional promising evidence for antibiofilm activity and potentiation of selected conventional antibiotics. In contrast, activity against Gram-negative bacteria is generally less pronounced, while antifungal and antiviral evidence remains comparatively limited and heterogeneous. Most findings are derived from in vitro experiments, with relatively little validation in clinically relevant infection models.

Interpretation is further complicated by substantial heterogeneity in microbial strains, experimental protocols, formulations, susceptibility methods, and reported antimicrobial potency. Moreover, both purified hyperforin and hyperforin-containing *H. perforatum* preparations have been investigated. This distinction is particularly important for antifungal and antiviral studies, where botanical preparations predominate and other constituents may contribute substantially to the observed activity. In antiviral studies specifically, some effects attributed to *H. perforatum* appear to be mediated primarily by hypericin or pseudohypericin rather than hyperforin.

Accordingly, the current literature supports experimental antimicrobial potential rather than established anti-infective efficacy. The most plausible translational opportunity may lie in adjunctive antibacterial and antibiofilm applications rather than replacement of established antimicrobial therapies. Future studies should prioritize purified and chemically characterized hyperforin, standardized antimicrobial susceptibility methods, clinically relevant infection and biofilm models, and pharmacokinetic–pharmacodynamic assessment to determine whether experimentally active concentrations can be achieved safely in vivo before clinical investigation.

The most relevant preclinical antimicrobial and antiviral findings discussed in Section 9 are summarized in Table 6, with emphasis on pathogen class, experimental context, intervention type, principal mechanisms, and major biological outcomes of hyperforin-related interventions.

## 10. Gastrointestinal and Gut Health Effects

### 10.1. Biological Rationale

The gastrointestinal tract plays a central role in nutrient absorption, immune regulation, metabolic signaling, and host–microbiota interactions, while disruption of epithelial barrier integrity, inflammatory and oxidative homeostasis, and microbial balance contributes to gastrointestinal and systemic disorders [262,263].

Although the gastrointestinal effects of hyperforin are less extensively characterized than its neuropharmacological actions, experimental evidence suggests that it may influence intestinal homeostasis through modulation of inflammatory and oxidative signaling, epithelial repair, and mucosal immune responses [264,265]. Reported mechanisms include suppression of pro-inflammatory cytokine production, inhibition of COX- and lipoxygenase-related pathways, attenuation of oxidative stress, and TRPC6-associated signaling relevant to epithelial survival and repair [264,265]. Its antimicrobial and antibiofilm properties may also influence the intestinal environment, although direct evidence for microbiota modulation remains limited.

### 10.2. Intestinal Barrier Integrity

#### 10.2.1. Tight Junction Regulation

The intestinal epithelial barrier limits the translocation of microbial products, dietary antigens, toxins, and other potentially harmful luminal constituents [265,266]. Its integrity depends substantially on intercellular junctional complexes, including tight-junction proteins such as occludin, claudins, and zonula occludens proteins [265,266]. Inflammatory cytokines, oxidative stress, and dysbiosis can disrupt these structures and increase intestinal permeability [267,268].

Indirect experimental evidence suggests that purified hyperforin and hyperforin-containing preparations may help preserve intestinal barrier function through suppression of inflammatory signaling and attenuation of oxidative injury [160,219,269]. By reducing TNF-α, IL-1β, and IL-6 and limiting oxidative damage to junctional proteins, hyperforin may help maintain epithelial cohesion and reduce pathological intestinal permeability [160,219,269]. However, direct experimental evidence demonstrating restoration of tight-junction architecture or intestinal permeability by purified hyperforin remains limited, and dedicated preclinical and clinical studies are required.

#### 10.2.2. Mucosal Protection

The intestinal mucosa is continuously exposed to dietary constituents, microbial metabolites, digestive enzymes, xenobiotics, and pathogens, and therefore requires coordinated epithelial renewal, immune regulation, antioxidant defense, and tissue-repair mechanisms [270].

Hyperforin has several biological activities potentially relevant to mucosal protection [207,219,261,271]. Its anti-inflammatory and antioxidant actions may limit cytokine-mediated epithelial injury and oxidative damage [207,219,261,272]. Hyperforin has also been reported to activate TRPC6-dependent signaling associated with ERK1/2-mediated cytoprotection, mitochondrial resilience, mitochondrial dynamics, and Lon protease-1 expression [207,219,261,272].

Its antimicrobial activity may further contribute to mucosal protection by limiting pathogenic microorganisms capable of disrupting epithelial integrity [176,224,229,233,259]. Nevertheless, although antimicrobial and antibiofilm properties are experimentally supported, direct evidence linking these effects specifically to improved intestinal mucosal health remains limited.

### 10.3. Anti-Inflammatory Effects in the Gut

Chronic intestinal inflammation contributes to epithelial injury, barrier dysfunction, immune dysregulation, and progressive tissue damage [273]. Consistent with the general anti-inflammatory and redox mechanisms described in Section 4, hyperforin-related interventions suppress NF-κB/STAT1-associated signaling, pro-inflammatory cytokines and enzymes, ROS generation, and immune-cell activation [8,61,66,170,176,219,270,274].

### 10.4. Interactions with the Gut Microbiota

#### 10.4.1. Microbial Composition

The gut microbiota influences nutrient metabolism, immune development, epithelial-barrier integrity, and colonization resistance, while dysbiosis has been associated with both gastrointestinal and systemic diseases [275].

Direct investigations of hyperforin–microbiota interactions remain limited. Nevertheless, purified hyperforin and hyperforin-containing preparations possess antimicrobial activity against several gastrointestinally relevant microorganisms, including Gram-positive bacteria, *Helicobacter pylori*, and multidrug-resistant strains, indicating the potential to exert selective pressure on microbial communities [219,229,276].

More importantly, animal studies provide preliminary evidence of microbiota modulation. In a chronic restraint-stress mouse model, hyperforin partially reversed stress-associated dysbiosis and improved anhedonia-like behavior [276]. A more recent study reported alterations in microbiota-associated fecal metabolites, increases in metabolites linked to beneficial taxa such as *Akkermansia muciniphila* and *Muribaculum intestinale*, improved colonic mucus-barrier integrity, and amelioration of depressive-like behaviors [277].

These findings suggest that gut microbiota may contribute to some physiological and neurobehavioral effects of hyperforin. However, the evidence remains limited, and future metagenomic, transcriptomic, metabolomic, and microbiome-sequencing studies are needed to characterize microbial community and functional responses more precisely.

#### 10.4.2. Intestinal Permeability and Microbial Biotransformation

Recent studies have begun to clarify the intestinal disposition of hyperforin and the potential contribution of the gut microbiota to the metabolism of *H. perforatum* constituents. Chauveau et al. (2023) investigated intestinal permeability using a Caco-2 model together with interactions with human intestinal microbiota and found that hyperforin exhibited high intestinal permeability [278]. Under the experimental conditions employed, hyperforin was not detectably metabolized by the microbiota and did not substantially affect microbial viability [278]. These findings support efficient intestinal membrane permeability of hyperforin while indicating that direct microbial biotransformation may depend strongly on the experimental system and exposure conditions.

More recently, Grafakou et al. (2025) investigated bidirectional interactions between a standardized *H. perforatum* extract and the human gut microbiome using gastrointestinal predigestion followed by incubation with human intestinal microbiota [279]. Their findings demonstrated microbial transformation of constituents of the multicomponent extract and suggested that *H. perforatum* may itself influence microbiome composition and function, potentially contributing to microbiome–gut–brain signaling [279]. However, because this study investigated a chemically complex *H. perforatum* preparation rather than purified hyperforin alone, the observed microbial transformations and microbiome effects cannot be attributed exclusively to hyperforin [279]. Collectively, these studies highlight the gut microbiome as a potentially important but still insufficiently characterized determinant of the intestinal fate and biological effects of hyperforin-containing preparations.

#### 10.4.3. Functional Consequences

Microbiota-derived metabolites, including short-chain fatty acids, secondary bile acids, indole derivatives, and neurotransmitter precursors, can influence intestinal barrier integrity and immune, metabolic, endocrine, and neurological functions [275]. Accordingly, microbiota-associated changes reported following hyperforin or *H. perforatum* exposure [276,277,278,279] may contribute to some systemic and neurobehavioral effects, potentially through modulation of inflammatory signaling and gut–brain communication [275,276,277].

However, a causal role of the microbiota in hyperforin pharmacology has not been established. The differing findings concerning microbial biotransformation [278,279] suggest that these effects may depend on the experimental system and on whether purified hyperforin or a multicomponent *H. perforatum* preparation is investigated. Consequently, effects observed with botanical extracts cannot be attributed specifically to hyperforin, and it remains unclear whether microbiota alterations mediate biological responses or represent secondary consequences of altered intestinal physiology.

Future studies integrating metagenomic and metabolomic profiling with intestinal-barrier assessment and mechanistic approaches, such as microbiota depletion, fecal microbiota transfer, or gnotobiotic models, are needed to establish causality, distinguish hyperforin-specific effects from those of the broader *H. perforatum* phytochemical matrix, and clarify the translational relevance of the microbiome–gut–brain axis.

### 10.5. Potential Applications in Gastrointestinal Disorders

#### 10.5.1. Irritable Bowel Syndrome

Irritable bowel syndrome (IBS) is a disorder of gut–brain interaction characterized by abdominal pain, altered bowel habits, visceral hypersensitivity, and altered neuroenteric signaling [280]. Low-grade inflammation, intestinal permeability, oxidative stress, microbiota disturbances, and altered gut–brain communication may contribute to its pathophysiology [280].

Direct studies evaluating hyperforin in established IBS models are currently lacking. Nevertheless, its anti-inflammatory, antioxidant, and barrier-protective actions may be relevant to mechanisms implicated in IBS. Hyperforin has been reported to suppress leukocyte activation, reduce ROS production, and inhibit inflammatory signaling pathways associated with mucosal immune activation [176,281]. Recent animal studies have also demonstrated improvements in colonic mucus-barrier integrity and microbiota-associated metabolic profiles following hyperforin administration [277,278].

Nevertheless, the potential role of hyperforin in IBS remains mechanistically plausible but unproven and requires direct investigation in disease-specific models before clinical relevance can be inferred.

#### 10.5.2. Experimental Colitis

Experimental colitis represents the best-developed gastrointestinal model. Hyperforin and hyperforin-containing preparations have reduced colonic inflammation, cytokine responses, immune-cell infiltration, oxidative stress, and histopathological injury while preserving mucosal architecture and epithelial-barrier function [170,282,283]. However, because several studies used multicomponent *H. perforatum* preparations, these findings provide preclinical proof-of-concept rather than compound-specific evidence for purified hyperforin.

### 10.6. Additional Emerging Gastrointestinal Applications

Hyperforin-containing interventions have also shown preliminary protective effects in experimental gastric, pancreatic, and hepatobiliary injury through inflammatory, redox, and tissue-protective mechanisms [62,63,66,156,160,188,284,285,286], while limited evidence suggests antimicrobial activity against H. pylori and other gastrointestinal pathogens [233]. Because most studies used multicomponent preparations and remain preclinical, the independent contribution and therapeutic relevance of hyperforin remain uncertain. Thus, further compound-specific studies are required to determine whether hyperforin has meaningful therapeutic activity in these gastrointestinal conditions or is better considered an adjunctive mucosal-protective compound.

### 10.7. Critical Appraisal for Current Evidence

Overall, the available evidence supports a preclinical gastrointestinal and mucosal-protective profile for hyperforin, involving anti-inflammatory, antioxidant, epithelial barrier-protective, antimicrobial, and microbiota-associated mechanisms. However, the strength of evidence varies considerably across gastrointestinal applications. Experimental colitis currently represents the most developed disease-specific area, whereas evidence for IBS, gastric disorders, microbiota-mediated effects, pancreatic injury, hepatobiliary conditions, and other proposed indications remains preliminary or largely mechanistic.

Interpretation is constrained by the predominance of chemically induced colitis models and the limited use of complementary chronic or clinically representative gastrointestinal disease models. In addition, both purified hyperforin and hyperforin-containing *H. perforatum* preparations have been investigated, limiting compound-specific attribution where botanical preparations were used. Direct evidence linking reported effects on intestinal barrier integrity, antimicrobial activity, or microbiota composition to sustained and clinically meaningful gastrointestinal outcomes also remains insufficient.

Accordingly, the current literature supports biological plausibility and preliminary preclinical gastrointestinal activity rather than established therapeutic efficacy. Future research should prioritize chemically characterized purified hyperforin, complementary disease-specific models, direct assessment of intestinal permeability and mucosal repair, integrated microbiome and metabolomic analyses, and pharmacologically realistic exposure conditions. Such studies are needed before the potential gastrointestinal applications of hyperforin can be meaningfully advanced toward clinical evaluation.

In addition, the relationship between experimentally active intestinal or systemic concentrations and exposures achievable following oral administration remains insufficiently characterized. Consequently, anti-inflammatory, barrier-protective, antimicrobial, and microbiota-associated effects demonstrated experimentally should not be assumed to reflect clinically achievable target engagement without supporting local or systemic pharmacokinetic data.

The most relevant preclinical gastrointestinal findings discussed in Section 10 are summarized in Table 7, with emphasis on the experimental model, intervention type, principal mechanisms, and major biological outcomes of hyperforin-related interventions.

## 11. Other Emerging Therapeutic Applications

### 11.1. Biological Rationale

Beyond its established neuropharmacological, anti-inflammatory, antimicrobial, and metabolic actions, hyperforin has been investigated in several emerging therapeutic contexts. Its effects on inflammatory and oxidative signaling, cellular differentiation, mitochondrial function, autophagy, and tissue repair provide a mechanistic rationale for potential applications involving impaired regeneration, chronic inflammation, degenerative processes, and age-related functional decline.

However, the maturity of evidence differs substantially among these applications. Wound healing and dermatological disorders have comparatively stronger experimental support, whereas evidence for pain management, musculoskeletal health, and healthy aging remains more limited and is predominantly preclinical or mechanistically inferred. Accordingly, the following subsections focus on application-specific evidence and its relative strength rather than reiterating the general mechanisms described earlier in this review.

### 11.2. Wound Healing

Wound healing requires coordinated inflammatory, proliferative, angiogenic, extracellular matrix, and remodeling responses, disruption of which can result in delayed or chronic healing [287]. Among the emerging therapeutic applications of hyperforin, wound repair is comparatively well investigated. Purified hyperforin and hyperforin-containing preparations promote keratinocyte differentiation and repair through TRPC6-dependent Ca^2+^ signaling and have been associated with improved wound closure, re-epithelialization, epidermal-barrier restoration, angiogenesis, extracellular-matrix remodeling, and collagen organization [154,271,288,289,290,291,292,293]. Anti-inflammatory and antioxidant effects [63,153,176,219,271] and antibacterial/antibiofilm activity against wound-relevant pathogens [161,220,223,289] may provide complementary benefits. However, many in vivo studies employed hyperforin-rich multicomponent preparations, limiting compound-specific attribution.

### 11.3. Dermatological Disorders

The skin provides a critical barrier against environmental, microbial, and chemical insults, and disruption of epidermal differentiation, inflammatory homeostasis, or barrier integrity contributes to several dermatological disorders [294,295]. Experimental evidence suggests that hyperforin may exert dermatoprotective effects through complementary anti-inflammatory, antioxidant, antimicrobial, and epidermal-regulatory mechanisms [153,161,271,296].

A particularly well-characterized mechanism involves TRPC6-dependent Ca^2+^ signaling in keratinocytes. Purified hyperforin promotes keratinocyte differentiation and increases the expression of epidermal differentiation markers, supporting epidermal maturation and barrier restoration [154,271,297]. These effects, together with suppression of inflammatory signaling, provide a mechanistic basis for its potential application in inflammatory skin disorders.

Among these conditions, psoriasis has comparatively stronger experimental support. Hyperforin-related interventions have been associated with reduced inflammatory signaling, modulation of keratinocyte proliferation and differentiation, and attenuation of psoriasis-associated immune responses [153,154,271,274]. Evidence for atopic dermatitis is more limited, although anti-inflammatory and barrier-supporting effects suggest potential therapeutic relevance [298].

Hyperforin may additionally contribute to dermatological protection through antibacterial and antibiofilm activity against skin- and wound-associated pathogens and through promotion of epithelial repair [70,154,233,271,299,300]. However, much of this evidence derives from topical *H. perforatum* preparations or multicomponent formulations, limiting attribution of the observed effects specifically to hyperforin. Overall, the dermatological evidence is promising, particularly for inflammatory skin conditions and topical applications, but further studies using chemically defined hyperforin preparations and controlled clinical designs are required.

### 11.4. Pain Management

Chronic pain involves interacting peripheral and central mechanisms, including inflammatory signaling, altered neurotransmission, oxidative stress, and neuronal sensitization [301]. Hyperforin and hyperforin-containing *H. perforatum* preparations have therefore been investigated for potential analgesic and antinociceptive effects.

Preclinical studies have demonstrated reduced hyperalgesia and improved nociceptive thresholds in models of neuropathic pain, including chronic constriction injury, diabetic neuropathy, and oxaliplatin-induced neuropathy [63,302,303,304]. Proposed mechanisms include modulation of monoaminergic and opioid-dependent signaling, attenuation of prostaglandin E_2_ and inflammatory responses, and regulation of TRPC6-associated Ca^2+^ signaling, neuroinflammation, and oxidative stress [63,302,303,304].

These findings support potential analgesic activity, particularly in neuropathic and inflammation-associated pain. However, much of the available evidence derives from *H. perforatum* preparations rather than purified hyperforin, making its independent contribution difficult to establish. Accordingly, the analgesic potential of hyperforin remains predominantly preclinical and requires confirmation using chemically defined hyperforin preparations and clinically relevant pain models.

### 11.5. Bone and Musculoskeletal Health

Musculoskeletal disorders, including osteoporosis, osteoarthritis, sarcopenia, and inflammatory arthropathies, involve varying degrees of inflammation, oxidative stress, impaired tissue remodeling, and structural degeneration [305]. Although evidence for hyperforin in this field remains limited, several preclinical findings suggest potential relevance to bone and joint homeostasis.

In experimental osteoarthritis models, tetrahydrohyperforin, a chemical analogue of hyperforin, attenuated cartilage degeneration and inflammatory changes while enhancing protective autophagy [306]. Hyperforin-related interventions have also been associated with suppression of PGE_2_ and pro-inflammatory cytokine production and attenuation of oxidative stress, processes relevant to bone and cartilage homeostasis [62,63].

Additional experimental evidence suggests potential promotion of osteoblast differentiation and mineralization together with suppression of osteoclast-mediated bone degradation [63,307]. These effects may favor bone preservation and, through attenuation of inflammatory signaling, potentially limit cartilage and synovial injury [63,307]. However, direct evidence demonstrating these effects specifically with purified hyperforin remains limited.

Overall, the musculoskeletal evidence should be considered preliminary. Findings obtained with tetrahydrohyperforin or hyperforin-containing preparations cannot be assumed to represent equivalent effects of purified hyperforin. Dedicated compound-specific studies are therefore required before potential applications in osteoporosis, osteoarthritis, rheumatoid arthritis, sarcopenia, or age-related musculoskeletal decline can be established.

### 11.6. Healthy Aging and Longevity

Aging is characterized by progressive molecular and cellular dysfunction involving oxidative stress, chronic low-grade inflammation, mitochondrial impairment, loss of proteostasis, and reduced regenerative capacity [308]. Several biological actions of hyperforin intersect with these processes, providing a mechanistic rationale for its potential relevance to healthy aging.

Experimental evidence suggests that hyperforin may support cellular resilience through antioxidant and anti-inflammatory effects, potentially limiting oxidative macromolecular damage and chronic inflammation associated with aging (“inflammaging”) [6,8]. Its effects on mitochondrial homeostasis, cellular bioenergetics, and autophagy may further support cellular quality control and the clearance of damaged proteins and organelles [5,8,309]. Neuroprotective actions involving TRPC6 and BDNF-associated signaling and attenuation of neuroinflammatory pathways may additionally contribute to preservation of neuronal homeostasis [5,8,309].

However, these findings derive predominantly from mechanistic and disease-oriented experimental studies rather than dedicated aging or longevity models. Direct evidence that hyperforin delays age-related functional decline or extends healthspan or lifespan remains insufficient. Accordingly, its potential geroprotective role should currently be regarded as hypothesis-generating rather than an established therapeutic application.

### 11.7. Critical Appraisal of Current Evidence

Overall, the emerging applications discussed in this section show markedly different levels of preclinical maturity. Wound healing and dermatological applications have the comparatively strongest experimental support, including tissue-specific and topical models demonstrating effects on epithelial repair, keratinocyte biology, inflammation, and antimicrobial protection. Pain management is supported by relevant animal studies but relies predominantly on hyperforin-containing *H. perforatum* preparations rather than purified hyperforin. In contrast, musculoskeletal and healthy-aging applications remain substantially less developed and are based mainly on limited preclinical evidence or mechanistic extrapolation.

Interpretation is constrained by the predominance of in vitro and animal studies, heterogeneous formulations and experimental protocols, and limited compound-specific evidence. The frequent use of hyperforin-containing *H. perforatum* preparations prevents unequivocal attribution of observed effects to hyperforin, while evidence involving hyperforin analogues should likewise not be considered equivalent to purified-hyperforin efficacy. Human evidence is scarce or absent for most indications and, where available—particularly in wound and dermatological applications—is limited by small samples, variable formulations and hyperforin content, and inconsistent outcome assessment.

Accordingly, these findings support promising but indication-dependent preclinical potential rather than established therapeutic efficacy. Wound healing and dermatological applications appear to warrant the highest priority for further translational investigation, whereas pain, musculoskeletal disorders, and healthy aging require substantially greater compound-specific and disease-specific validation. Future studies should prioritize chemically characterized hyperforin, clinically relevant formulations and exposure levels, robust disease-specific models, and appropriately designed human studies before therapeutic applications can be established. For topical applications, local tissue exposure may be more relevant than systemic plasma concentrations, whereas systemic indications require demonstration that experimentally active concentrations can be reproduced in the relevant target tissue following clinically feasible dosing.

The most relevant preclinical findings across the emerging applications discussed in Section 11 are summarized in Table 8, with emphasis on the experimental model, intervention type, principal biological mechanisms, and relative maturity of the available evidence.

## 12. Human Clinical Evidence of Hyperforin-Containing *H. perforatum* Preparations

### 12.1. Overview of Clinical Research

An important consideration when interpreting the available clinical evidence is that almost all RCTs have evaluated hyperforin-containing preparations (standardized or non-standardized *H. perforatum* extract preparations or formulations) rather than purified hyperforin. Because these preparations contain numerous biologically active constituents, including hypericin, flavonoids, phenolic acids, and other phytochemicals, the observed therapeutic effects cannot be attributed exclusively to hyperforin. Although mechanistic, cellular, and animal studies strongly support hyperforin as one of the principal bioactive constituents responsible for many pharmacological actions of *H. perforatum*, synergistic or additive interactions among multiple constituents may also contribute to the clinical efficacy of standardized extracts. To avoid duplication with the preceding disease-specific sections, the present section provides the detailed synthesis of human clinical evidence, whereas Section 5, Section 6, Section 7, Section 8, Section 9, Section 10 and Section 11 emphasize mechanistic and preclinical findings.

Moreover, adequately powered RCTs evaluating purified hyperforin as a single therapeutic agent are currently lacking. Therefore, the independent clinical efficacy of hyperforin remains to be established, and future studies should directly compare purified hyperforin with standardized hyperforin-containing preparations or formulations to clarify their relative therapeutic contributions.

Throughout this section, the nature of the intervention underlying the clinical evidence is explicitly distinguished. Unless otherwise stated, clinical efficacy findings refer to standardized or otherwise characterized *H. perforatum* preparations containing hyperforin together with other phytochemical constituents rather than to purified hyperforin administered as a single therapeutic agent. Accordingly, efficacy demonstrated with such multicomponent preparations is interpreted as evidence for the preparation studied and not as direct proof of the independent clinical efficacy of hyperforin. Compound-specific therapeutic conclusions are therefore restricted to evidence obtained with purified or chemically defined hyperforin, which remains very limited in human studies.

The clinical evidence base comprises RCTs, comparative effectiveness studies, observational investigations, systematic reviews, and meta-analyses. The majority of these studies have focused on depressive disorders, particularly mild-to-moderate major depression, whereas a smaller number have examined anxiety symptoms, stress-related disorders, cognitive outcomes, dermatological applications, wound healing, and inflammatory conditions. More recently, interest has expanded toward the potential role of hyperforin-containing preparations in neurodegenerative, metabolic, cardiovascular, gastrointestinal, and immune-mediated disorders; however, clinical evidence in these areas remains preliminary and insufficient to support definitive therapeutic recommendations.

It should be noted that the interpretation of the clinical literature is highly complicated by substantial methodological heterogeneity. Variations in extract standardization, hyperforin content, treatment duration, dosing regimens, patient populations, outcome measures, and study design contribute to inconsistencies among findings. Furthermore, standardized *H. perforatum* extracts, preparations and formulations frequently contain differing concentrations of hyperforin, hypericin, flavonoids, and other phytochemicals, thereby limiting the ability to establish clear dose–response relationships and accurately attribute therapeutic outcomes to hyperforin alone.

### 12.2. Depression and Mood Disorders

#### 12.2.1. Clinical Trials

Among all investigated indications, depression represents the therapeutic area for which the strongest clinical evidence currently exists. Several RCTs have evaluated the efficacy of standardized hyperforin-containing preparations in patients with mild-to-moderate major depressive disorder, dysthymia, adjustment disorders, and depressive symptoms associated with chronic stress and psychosocial burden.

Across multiple studies, treatment with hyperforin-containing preparations has been associated with significant reductions in depressive symptom severity and improvements in psychological well-being, emotional functioning, and quality of life, particularly in patients with mild-to-moderate depression [108,109,118,310,311,312,313,314,315,316]. These benefits have been documented using validated psychometric instruments, including the Hamilton Depression Rating Scale (HAM-D), Montgomery–Åsberg Depression Rating Scale (MADRS), Beck Depression Inventory (BDI), and Clinical Global Impression (CGI) scale. Clinical improvements generally become evident after several weeks of treatment and have been observed across diverse patient populations, including adults, elderly individuals, and patients with recurrent depressive episodes [108,109,118,310,311,312,313,314,315,316].

Several investigations have reported associations between the hyperforin content of *H. perforatum* preparations and antidepressant efficacy, supporting a possible or important contribution of hyperforin to the activity of these extracts [108,109,118,310,311,312,313,314,315,316]. However, such associations do not establish that hyperforin independently accounts for the observed clinical effect, because the preparations contain multiple pharmacologically active constituents. It should be noted that most of the high-quality clinical trials specifically evaluating hyperforin-containing preparations were conducted between 1998 and 2010. Since then, relatively few large RCTs have been performed, and recent publications have focused more on systematic reviews, meta-analyses, comparative effectiveness studies, and observational research rather than new pivotal RCTs.

#### 12.2.2. Comparative Efficacy

Evidence from systematic reviews and meta-analyses generally supports the efficacy of standardized *H. perforatum* preparations over placebo in mild-to-moderate depression, although effect estimates and conclusions vary according to the preparations, populations, and trials included [104,106,316,317,318,319,320,321]. Comparative trials have reported therapeutic effects almost similar to those achieved with selective serotonin reuptake inhibitors (SSRIs), tricyclic antidepressants (TCAs), and other antidepressant classes [104,106,316,317,318,319,320,321]. These comparative findings apply to the specific multicomponent *H. perforatum* preparations evaluated and should not be interpreted as demonstrating therapeutic equivalence between purified hyperforin and conventional antidepressant drugs.

Several studies have also shown comparable reductions in depressive symptom scores between hyperforin-containing preparations and commonly prescribed antidepressants, including fluoxetine, sertraline, paroxetine, citalopram, and imipramine [104,106,316,317,318,319,320,321]. Moreover, patients receiving hyperforin-containing preparations frequently demonstrate higher treatment adherence and lower discontinuation rates, findings largely attributed to improved tolerability and fewer adverse effects [104,106,316,317,318,319,320,321].

Nevertheless, evidence supporting efficacy in severe major depressive disorder, treatment-resistant depression, bipolar depression, and psychotic depression remains considerably weaker. Consequently, most evidence-based guidelines recommend St. John’s Wort primarily for mild-to-moderate depressive disorders rather than severe psychiatric conditions. Consistent with this clinical evidence, specified *H. perforatum* herbal preparations are recognized by the EMA/HMPC under a well-established-use indication for mild to moderate depressive episodes [110].

Despite the generally promising evidence for standardized *H. perforatum* preparations in mild-to-moderate depression, the magnitude and consistency of antidepressant effects vary among individual trials. Several factors may contribute to this heterogeneity. First, preparations differ substantially in extraction procedure, total extract dose, hyperforin concentration, hypericin and flavonoid content, and pharmaceutical formulation; therefore, products nominally classified as *H. perforatum* extracts cannot necessarily be regarded as pharmacologically equivalent. Second, trials differ in baseline depression severity, diagnostic criteria, duration of treatment, comparator, outcome instrument, and definitions of response or remission. Differences in adherence, concomitant medication use, study setting, and population characteristics may further contribute to between-study variability. Importantly, a relationship between hyperforin content and antidepressant efficacy has been suggested in some studies, but this does not establish hyperforin as the sole active constituent because other components of the phytocomplex may exert additive, synergistic, or pharmacokinetic effects. Thus, the relatively consistent evidence supporting standardized *H. perforatum* extracts in mild-to-moderate depression should not be interpreted as equivalent evidence for purified hyperforin, and variation among products remains an important limitation when comparing clinical studies.

### 12.3. Other Clinical Applications

#### 12.3.1. Anxiety Disorders

Although anxiety has received less clinical investigation than depression, hyperforin-containing preparations have shown preliminary benefit in generalized anxiety symptoms, mixed anxiety–depressive states, adjustment disorders, and stress-related psychopathology [311,322,323,324,325]. However, dedicated RCTs in primary anxiety disorders remain limited, precluding definitive therapeutic recommendations.

#### 12.3.2. Cognitive Health

Clinical investigations evaluating cognitive outcomes remain relatively scarce. Nevertheless, preliminary evidence suggests that hyperforin-containing preparations may improve attention, memory performance, executive function, and overall cognitive efficiency, particularly among individuals experiencing depressive symptoms, chronic stress, or age-related cognitive decline [326,327,328,329]. Preliminary clinical evidence suggests that hyperforin-containing preparations may improve attention, memory, executive function, and cognitive efficiency, particularly in individuals with depressive symptoms, chronic stress, or age-related cognitive decline [326,327,328,329]. These findings are biologically consistent with the preclinical mechanisms discussed in Section 4 and Section 5; however, large RCTs with cognition as a primary endpoint are lacking [14,64,330,331].

#### 12.3.3. Inflammatory Disorders

Clinical evidence outside neuropsychiatric indications remains limited. Topical hyperforin-rich preparations have shown benefit in atopic dermatitis and other dermatological applications, including improved tissue repair and barrier function with reduced inflammatory and oxidative responses [271,299,332,333,334,335]. However, high-quality RCTs investigating purified hyperforin in autoimmune diseases, inflammatory bowel disease, rheumatoid arthritis, or other chronic inflammatory disorders remain largely absent. Consequently, the majority of evidence supporting anti-inflammatory applications continues to derive from mechanistic and preclinical research.

### 12.4. Strength of Evidence

Currently, there is no clinical study assessing the potential beneficial effects of the purified hyperforin in any disease area. The strongest evidence currently supports the use of hyperforin-containing preparations in mild-to-moderate major depressive disorder, depressive symptoms associated with stress-related conditions, and the improvement of mood and emotional well-being. These indications are supported by several RCTs, systematic reviews, and meta-analyses demonstrating efficacy superior to placebo and broadly comparable to conventional antidepressant therapies. However, these potential therapeutic indications should be interpreted with appropriate caution, as the available RCTs demonstrate considerable variability in study populations, intervention protocols, outcome assessments, and methodological quality, thereby limiting the strength and generalizability of the current clinical evidence. The use of hyperforin-containing preparations instead of purified hyperforin remains an additional challenge.

Moderate evidence exists for the management of anxiety symptoms associated with depression, stress-related psychological disturbances, quality-of-life enhancement, and certain dermatological and wound-healing applications. Although encouraging clinical findings have been reported, the number of well-designed studies remains considerably smaller than that available for depression. Thus, more well-designed RCTs should be performed in order for reliable conclusions to be drawn, using purified hyperforin and not hyperforin-containing preparations as the principal treatment approach.

In contrast, evidence remains preliminary for neurodegenerative disorders, cognitive decline, AD, PD, cardiovascular disease, metabolic syndrome, type 2 diabetes mellitus, inflammatory bowel disease, autoimmune disorders, cancer prevention, adjunctive oncological applications, and healthy aging. In these areas, current support derives primarily from mechanistic investigations, cellular experiments, and, to a lesser extent, animal studies, with only limited human clinical validation. Moreover, in several of these disease areas, clinical evidence is completely lacking.

A small open-label clinical study evaluated hyperforin-containing preparations in patients with dementia who exhibited neuropsychiatric symptoms such as agitation, apathy, depression, and behavioral disturbances. Improvements were reported in several behavioral and psychological symptoms associated with dementia, although the study was small and uncontrolled [329,336].

In Table 4, the available clinical evidence on the hyperforin-containing preparations and formulations across disease domains is summarized. It should be noted that the indicated treatment durations represent approximate ranges across the clinical studies summarized within each disease domain. Exact treatment periods varied according to study design, population, formulation, dosage regimen, and outcome assessment. Almost all clinical investigations evaluated standardized hyperforin-containing preparations rather than purified hyperforin; consequently, the reported clinical effects cannot be attributed exclusively to hyperforin. Hence, the information summarized in Table 9 should be interpreted within the context of the overall quality and type of evidence available for each disease area.

### 12.5. Comparison with Conventional Therapies

#### 12.5.1. Efficacy

For mild-to-moderate depressive disorders, hyperforin-containing preparations demonstrate therapeutic efficacy that is broadly comparable to many conventional antidepressants. Several comparative studies have reported similar improvements in depressive symptom severity and psychological functioning [101,103,106,116,118,317,337].

However, unlike conventional antidepressants, which generally target a limited number of neurotransmitter systems, hyperforin exerts a multimodal pharmacological profile encompassing neurotransmission, neuroplasticity, neurotrophic signaling, inflammation, oxidative stress, mitochondrial function, and cellular energy metabolism [8,14,54]. This broader spectrum of biological activity may offer advantages in complex disorders characterized by multifactorial pathophysiology.

Nonetheless, conventional pharmacotherapies generally benefit from a substantially larger clinical evidence base, more clearly established dosing protocols, and stronger regulatory approval frameworks, particularly for severe psychiatric illnesses and chronic systemic diseases. Conversely, the clinical studies evaluating hyperforin-containing preparations are characterized by considerable methodological heterogeneity and several important limitations, which complicate interpretation of the findings and may overestimate the independent contribution of hyperforin to the reported antidepressant effects, particularly because most investigations have evaluated standardized hyperforin-containing preparations rather than purified hyperforin.

#### 12.5.2. Comparative Safety with Conventional Therapies

One of the potential clinical advantages of hyperforin-containing *H. perforatum* preparations is their generally favorable tolerability profile relative to several conventional antidepressant therapies. Clinical studies have reported lower overall rates of treatment-emergent adverse events, with reported reactions generally being mild and transient, most commonly including gastrointestinal discomfort, headache, dizziness, fatigue, and dry mouth [2,317,321,322,338,339]. These findings are particularly relevant in the treatment of mild-to-moderate depressive disorders, where tolerability may substantially influence adherence and treatment persistence.

Compared with many conventional antidepressants, hyperforin-containing preparations have also been associated with lower incidences of sexual dysfunction, body weight gain, sedation, and anticholinergic adverse effects [103,107,317,339]. Because these adverse effects can negatively affect quality of life and contribute to treatment discontinuation during conventional antidepressant therapy, the comparatively favorable tolerability profile of *H. perforatum* preparations may represent a clinically meaningful advantage in appropriately selected patients [2,103,107,317,321,322,338,339]. Nevertheless, this advantage should be interpreted cautiously, as it primarily concerns the burden of common treatment-related adverse effects and does not imply an absence of clinically important safety risks.

Most importantly, this tolerability advantage must be balanced against the substantial drug-interaction liability associated with hyperforin-containing preparations. Hyperforin-mediated activation of the PXR, with subsequent induction of CYP3A4, P-glycoprotein, and other drug-metabolizing and transport systems, can produce clinically significant pharmacokinetic interactions with concomitant medications [43,174,340,341]. These interactions are particularly relevant in patients receiving multiple pharmacotherapies and may limit the suitability of hyperforin-containing preparations despite their otherwise favorable tolerability profile. The molecular mechanisms, affected drug classes, and clinical implications of these interactions are discussed comprehensively in Section 13.6.

Accordingly, the safety profile of hyperforin-containing *H. perforatum* preparations should be regarded as different rather than unequivocally superior to that of conventional antidepressants. Their lower burden of several common adverse effects must be weighed against a potentially greater risk of clinically significant pharmacokinetic drug interactions [43,174,340,341]. Careful medication review and individualized risk–benefit assessment are therefore essential when considering their use, particularly in patients receiving concomitant medications.

#### 12.5.3. Cost-Effectiveness

Direct pharmacoeconomic evidence remains limited, with only a small number of formal economic evaluations having been conducted. Nevertheless, available analyses suggest that standardized hyperforin-containing preparations may represent a cost-effective treatment option for mild-to-moderate depressive disorders [107,321,342,343]. Comparable clinical efficacy, lower rates of adverse events, improved treatment adherence, reduced treatment discontinuation, and lower healthcare utilization associated with adverse-effect management appear to contribute to favorable economic outcomes relative to several conventional antidepressants [107,321,342,343].

However, it should be noted that economic outcomes associated with healthcare interventions, including phytotherapeutic products, vary substantially across jurisdictions owing to differences in healthcare-system organization, reimbursement policies, regulatory requirements, health technology assessment procedures, product standardization, and local prescribing practices [344,345]. Consequently, cost-effectiveness findings generated in one setting may not be directly generalized to other healthcare environments, highlighting the importance of context-specific economic evaluations. Moreover, concerns regarding product quality, variability in hyperforin concentration, and lack of international standardization remain important barriers to broader clinical implementation, highlighting cost-effectiveness issues [344,345].

### 12.6. Critical Appraisal and Translational Integration of Human Clinical Evidence

Collectively, the available human evidence reveals a marked imbalance between the breadth of the experimental pharmacology of hyperforin and the maturity of its clinical validation. Standardized hyperforin-containing *H. perforatum* preparations are supported by the strongest clinical evidence in mild-to-moderate depressive disorders, where RCTs and evidence syntheses indicate clinically relevant efficacy and generally favorable tolerability. More limited human evidence suggests potential benefits in anxiety-related and stress-associated symptoms and selected dermatological applications. In contrast, proposed applications in neurodegenerative, cardiometabolic, gastrointestinal, immune-mediated, oncological, infectious, musculoskeletal, and healthy-aging conditions remain predominantly supported by mechanistic and preclinical findings rather than definitive clinical trials.

An important limitation across the clinical literature is the persistent disconnect between the compound investigated mechanistically and the interventions evaluated in humans. Most molecular and cellular studies have examined purified hyperforin, whereas clinical investigations have predominantly employed chemically complex *H. perforatum* extracts containing hyperforin together with several other biologically active constituents. Consequently, the clinical efficacy observed with standardized botanical preparations cannot be attributed exclusively to hyperforin, and the therapeutic dose, dose–exposure relationship, pharmacodynamic profile, long-term safety, and independent clinical efficacy of purified hyperforin remain insufficiently defined.

Translation is further constrained by pharmaceutical and methodological factors. Poor aqueous solubility, chemical instability, variable oral exposure, formulation-dependent pharmacokinetics, heterogeneity in extract composition and hyperforin content, and clinically relevant drug-interaction liability complicate the transition from promising experimental activity to reproducible clinical efficacy. At the clinical-study level, variation in formulations, doses, treatment duration, study populations, comparators, and outcome measures further limits direct comparison among trials and contributes to uncertainty regarding the generalizability of the available findings.

Furthermore, the current evidence suggests that the principal translational challenge is no longer simply to demonstrate additional biological activity, but to establish a coherent pathway linking mechanistically validated targets to reproducible pharmacological exposure, clinically relevant target engagement, demonstrable therapeutic efficacy, acceptable safety, and eventual real-world implementation. This progression requires chemically characterized and preferably purified hyperforin formulations, optimized delivery systems, rigorous human pharmacokinetic and dose-finding studies, biomarker-informed early-phase investigations, adequately powered RCTs, and subsequent assessment of long-term effectiveness, safety, interaction liability, and implementation feasibility.

Accordingly, three levels of clinical inference should be distinguished: evidence for the efficacy of a defined *H. perforatum* preparation; evidence suggesting that hyperforin content may influence the activity of that preparation; and direct evidence for the independent efficacy of purified hyperforin. The current clinical literature provides substantial support for the first level in mild-to-moderate depression, some indirect support for the second, but remains insufficient for the third. These levels should not be treated as interchangeable.

## 13. Overview of Safety, Toxicology, and Drug–Drug Interactions Issues

### 13.1. Introductory Interpretations

Assessment of the therapeutic potential of hyperforin requires consideration of its safety, toxicological profile, pharmacokinetic behavior, and interaction liability. Although hyperforin-containing preparations generally show an acceptable safety profile in experimental and clinical settings, uncertainties remain regarding long-term exposure, product standardization, interindividual variability, and clinically significant drug–drug interactions [43,345,346,347,348,349,350,351].

Direct mechanistic evidence identifies hyperforin as an important contributor to the pharmacokinetic interaction potential of *H. perforatum* through activation of xenobiotic-sensing pathways and induction of drug-metabolizing enzymes and transport proteins [43,340,346,347,348,352]. However, clinically observed interaction profiles are generally derived from multicomponent *H. perforatum* preparations and therefore should not be attributed exclusively to hyperforin. Accordingly, evaluation of both intrinsic toxicity and interaction-mediated risk is essential for defining its therapeutic window and guiding safe clinical and pharmaceutical development.

### 13.2. General Safety Profile

Current evidence indicates that standardized hyperforin-containing *H. perforatum* preparations are generally well tolerated during short- to medium-term use, with low rates of treatment discontinuation and serious adverse events across randomized controlled trials, comparative studies, systematic evaluations, and post-marketing surveillance [1,101,103,107,317,319,322,339,346,353,354,355,356,357,358,359,360,361,362]. However, these findings primarily concern multicomponent botanical preparations and should not be directly extrapolated to purified hyperforin, particularly during prolonged exposure or when using formulations designed to increase systemic bioavailability [43,346,352,360,361,363].

Comparative studies generally indicate that standardized *H. perforatum* preparations are as well or better tolerated than several conventional antidepressants, with a lower burden of some treatment-associated adverse effects [101,103,317,319,353,357,358,360,361]. Nevertheless, favorable tolerability does not imply an absence of clinically relevant safety concerns.

The safety profile of hyperforin is influenced by exposure-, formulation-, and patient-related factors, including dose and treatment duration, hyperforin content and bioavailability, age and clinical status, genetic variability, comorbidities, and concomitant pharmacotherapy [1,43,346,349,360,362,363]. Importantly, these factors affect not only intrinsic toxicity but also the likelihood and magnitude of pharmacokinetic interactions. Thus, hyperforin-containing preparations may exhibit relatively favorable short- to medium-term clinical tolerability, whereas the intrinsic tolerability and therapeutic margin of purified hyperforin in humans remain insufficiently characterized, an issue addressed in detail in Section 13.5 [43,346,349,352].

### 13.3. Toxicological Evidence

#### 13.3.1. Acute Toxicity

Available toxicological evidence indicates that hyperforin has relatively low acute toxicity. Animal studies of purified hyperforin and hyperforin-containing preparations have reported high LD_50_ values and limited evidence of acute organ toxicity, with adverse effects occurring predominantly at supratherapeutic exposures [1,2,136,346,355,364,365,366,367,368]. These findings are broadly consistent with clinical experience showing that standardized *H. perforatum* preparations are generally well tolerated at recommended doses, with serious acute toxic effects being uncommon [1,101,338,346,355,358,360,361,363].

Nevertheless, experimental LD_50_ data and the tolerability of multicomponent botanical preparations cannot establish the acute safety profile or therapeutic margin of purified hyperforin in humans. Thus, current evidence supports relatively low acute toxicity under experimental and conventional-use conditions, but compound-specific human safety data remain limited.

#### 13.3.2. Chronic Toxicity

Long-term toxicological data specific to purified hyperforin remain limited. Repeated-dose animal studies and longer-term investigations of standardized hyperforin-containing *H. perforatum* preparations have generally reported little evidence of cumulative organ toxicity under the conditions examined [317,339,360,361,363,364,366,369,370,371,372,373,374,375,376,377]. However, differences in preparation, dose, exposure duration, and systemic bioavailability limit direct extrapolation to prolonged treatment with purified hyperforin.

An additional concern during chronic exposure is sustained activation of PXR-dependent pathways, with consequent induction of CYP3A4 and drug transporters [43,174,352,369,370,371,372,373,374,375,376,377]. This effect is better regarded as chronic pharmacokinetic interaction liability rather than conventional cumulative organ toxicity, but it may substantially influence clinical safety during prolonged treatment, particularly in patients receiving concomitant medications.

Overall, the available evidence does not indicate a strong cumulative toxicological signal, but the limited compound-specific and long-term data preclude definitive conclusions regarding the chronic safety of purified hyperforin.

### 13.4. Adverse Effects

#### 13.4.1. Common Adverse Events

The adverse effects most frequently associated with hyperforin-containing preparations are generally mild and reversible. Commonly reported events include gastrointestinal discomfort, nausea, abdominal pain, headache, dizziness, fatigue, xerostomia, mild agitation, restlessness, and sleep disturbances [1,101,108,355,360,361]. These symptoms typically occur infrequently and rarely necessitate treatment discontinuation.

Photosensitivity reactions have occasionally been reported, particularly following exposure to high-dose hyperforin-containing preparations. However, these effects are generally attributed primarily to hypericin rather than hyperforin, and clinically significant photosensitivity remains uncommon at standard therapeutic doses [378,379,380,381].

Importantly, hyperforin-containing preparations consistently demonstrate lower incidences of sexual dysfunction, sedation, weight gain, cognitive impairment, and anticholinergic effects than many conventional antidepressant agents, thereby contributing to improved treatment adherence and patient acceptability [101,103,104,317,339].

#### 13.4.2. Serious Adverse Events

Serious adverse events attributable to hyperforin are relatively uncommon; nevertheless, several clinically relevant safety concerns have been identified. The most notable involves the potential development of serotonin syndrome when hyperforin-containing preparations are combined with serotonergic agents, including SSRIs, SNRIs, monoamine oxidase inhibitors, certain opioid analgesics, and selected antimigraine medications [346,382,383]. Excessive serotonergic stimulation may result in autonomic instability, neuromuscular abnormalities, altered mental status, and, in severe cases, life-threatening complications.

Isolated cases of mania and hypomania have also been reported, particularly among individuals with bipolar spectrum disorders or pre-existing mood instability [384,385,386,387,388]. Consequently, caution is warranted when hyperforin-containing preparations are administered to susceptible populations.

Although hepatotoxicity has occasionally been reported, available evidence does not support a strong causal association between hyperforin or hyperforin-containing preparations exposure and clinically significant liver injury [1,43,361,389,390]. Systematic reviews, RCTs, and pharmacovigilance analyses have generally failed to identify a consistent hepatotoxic signal, and St. John’s wort is considered an unlikely cause of clinically apparent liver injury [1,43,361,389,390].

In contrast, an important safety concern is the pharmacokinetic interaction liability of hyperforin-containing preparations. Direct mechanistic evidence demonstrates that hyperforin activates PXR-dependent pathways and induces CYP450 enzymes and drug transporters, providing a strong mechanistic basis for many of the clinically relevant interactions observed with *H. perforatum* products [1,43,361,389,390].

### 13.5. Drug–Herb Interactions

Drug–herb interactions constitute a major limitation to the clinical use of hyperforin-containing *H. perforatum* preparations and represent an important safety concern associated with these products. A distinction should, however, be made between compound-specific mechanistic evidence and clinical interaction evidence obtained with multicomponent botanical preparations. Mechanistic studies provide strong evidence that hyperforin activates PXR, resulting in induction of CYP3A4, P-glycoprotein, and other xenobiotic-metabolizing systems, thereby establishing hyperforin as a major mechanistic contributor to the pharmacokinetic interaction potential of *H. perforatum* [43,173,174,391]. In contrast, most clinically documented herb–drug interactions have been demonstrated using *H. perforatum* extracts or products containing hyperforin together with multiple other bioactive constituents. These clinical observations therefore establish the interaction liability of the preparations studied but do not, by themselves, demonstrate that the observed effects are attributable exclusively to hyperforin. Nevertheless, the association between hyperforin exposure or content and the magnitude of enzyme induction provides complementary evidence supporting an important contribution of hyperforin to the interaction potential of these preparations [346].

#### 13.5.1. CYP3A4 Induction

Hyperforin functions as a high-affinity ligand of PXR, a xenobiotic-sensing nuclear receptor that serves as a master regulator of genes involved in drug metabolism and transport. Upon activation, PXR induces the transcription of multiple drug-metabolizing enzymes and transporters, most notably CYP3A4 and P-glycoprotein [43,173,174,391]. Because CYP3A4 is responsible for the metabolism of more than half of clinically prescribed medications, hyperforin-mediated PXR activation provides a well-established molecular mechanism that can account for a substantial component of the pharmacokinetic interaction potential of hyperforin-containing *H. perforatum* preparations [43,173,174,391]. This mechanistic attribution should be distinguished from clinical interaction studies conducted with multicomponent *H. perforatum* preparations, in which the contribution of hyperforin cannot always be isolated from that of the complete phytochemical matrix.

Consequently, hyperforin-induced CYP3A4 expression can accelerate drug clearance, reduce systemic drug exposure, diminish therapeutic efficacy, and increase the risk of treatment failure. The magnitude of this effect depends on hyperforin dose, duration of administration, formulation characteristics, and interindividual variability.

#### 13.5.2. P-Glycoprotein Induction

In addition to CYP3A4 induction, hyperforin markedly increases expression of P-glycoprotein (ABCB1/MDR1), an ATP-dependent efflux transporter expressed in the intestinal epithelium, liver, kidneys, blood–brain barrier, and other tissues [173,174,382,392,393]. Activation of PXR by hyperforin upregulates ABCB1 transcription, leading to increased transporter expression and activity. Enhanced P-glycoprotein-mediated efflux reduces intestinal absorption, decreases oral bioavailability, and lowers systemic exposure to numerous therapeutic agents, thereby contributing substantially to clinically significant herb–drug interactions associated with hyperforin-containing preparations [173,174,382,392,393].

Collectively, the combined induction of CYP3A4 and P-glycoprotein creates a highly effective pharmacokinetic interaction mechanism capable of significantly altering the disposition of numerous therapeutic agents.

#### 13.5.3. Interactions with Therapeutic Agents

The PXR-mediated induction of CYP3A4 and P-glycoprotein by hyperforin provides a strong mechanistic basis for reduced exposure to susceptible concomitant medications. Clinically, however, the majority of documented interactions have been reported following administration of multicomponent *H. perforatum* preparations rather than purified hyperforin. Accordingly, the clinical findings summarized below should primarily be interpreted as evidence of herb–drug interactions associated with the preparations studied, with hyperforin representing a mechanistically well-supported contributor rather than an exclusively established causal constituent.

Concomitant use of hyperforin-containing *H. perforatum* preparations with serotonergic antidepressants may increase the risk of serotonergic toxicity and serotonin syndrome, particularly with selective serotonin reuptake inhibitors, serotonin–norepinephrine reuptake inhibitors, tricyclic antidepressants, monoamine oxidase inhibitors, and other serotonergic agents [349,382,383,394]. Such combinations should therefore generally be avoided or undertaken only with appropriate clinical supervision.

Interactions with oral contraceptives are also clinically important. Hyperforin-containing preparations may reduce circulating concentrations of ethinyl estradiol and progestins, leading to breakthrough bleeding, enhanced follicular development or ovulation, and potentially reduced contraceptive efficacy [395,396,397,398,399]. These findings support avoidance of high-hyperforin preparations during hormonal contraceptive use or the use of appropriate alternative contraceptive measures.

Particularly serious interactions occur with immunosuppressive agents, including cyclosporine, tacrolimus, and sirolimus. Hyperforin-mediated induction of drug metabolism and transport can markedly reduce systemic exposure to these agents, resulting in subtherapeutic concentrations and, in transplant recipients, an increased risk of graft rejection [348,400,401,402,403].

Clinically relevant interactions have also been documented with anticoagulants. Hyperforin-containing preparations may reduce warfarin anticoagulant activity and international normalized ratio (INR), while pharmacokinetic studies indicate reduced exposure and pharmacodynamic activity of direct oral anticoagulants such as rivaroxaban [382,404,405,406]. These interactions may compromise anticoagulant efficacy and warrant avoidance or careful clinical monitoring.

Interactions with anticancer agents are similarly concerning because reduced systemic drug exposure may compromise therapeutic efficacy. Decreased exposure has been reported for agents including irinotecan and its active metabolite SN-38, imatinib, and docetaxel following concomitant use of *H. perforatum* preparations [348,407,408,409,410]. Given the narrow therapeutic window and potential consequences of treatment failure, concomitant use with anticancer therapy should generally be avoided unless specifically evaluated and closely supervised.

Overall, these findings demonstrate that the clinical safety of hyperforin-containing preparations depends not only on their intrinsic tolerability but also on concomitant pharmacotherapy. The interaction risk is particularly important for medications with narrow therapeutic indices or for which modest reductions in systemic exposure may compromise treatment efficacy.

### 13.6. Regulatory Considerations

Regulatory authorities have recognized both the therapeutic use and safety concerns associated with *H. perforatum* products, particularly the substantial variability in hyperforin content and its implications for pharmacokinetic interactions. Accordingly, regulatory considerations focus primarily on product standardization, interaction risk, appropriate labeling, and patient counseling.

European Food Safety Authority (EFSA) assessments have highlighted marked variability in hyperforin content among commercially available *H. perforatum* preparations, with differences exceeding 60-fold in some analyses [346,411,412,413,414]. Importantly, hyperforin content correlates with the magnitude of CYP3A4 induction, indicating that product composition is a major determinant of interaction potential [346,411,412,413,414]. These findings emphasize the importance of standardized preparations and quantitative characterization of hyperforin exposure when evaluating product safety.

In the United States, *H. perforatum* products are generally marketed within the dietary-supplement framework. The U.S. Food and Drug Administration (FDA) has issued communications concerning clinically significant interactions with prescription medications, particularly those resulting from induction of CYP450 enzymes and drug transporters, and emphasizes appropriate disclosure and consultation with healthcare professionals [346,415,416,417].

Similarly, European Medicines Agency (EMA) monographs recognize *H. perforatum* as a herbal medicinal product while emphasizing quality control, standardized manufacturing, contraindications, and clinically relevant drug interactions [43,418,419,420,421,422]. Particular attention is directed toward hyperforin because of its central role in PXR activation and subsequent induction of CYP450 enzymes and P-glycoprotein [43,418,419,420,421,422]. EMA guidance therefore emphasizes interaction warnings and professional counseling, especially in patients receiving concomitant prescription medications.

Overall, these regulatory perspectives converge on a common principle: the safety of *H. perforatum* products depends not only on intrinsic tolerability but also on standardized hyperforin content and appropriate management of drug-interaction risk. Greater consistency in product characterization and labeling is therefore essential for predictable pharmacological activity and safer clinical use.

### 13.7. Critical Appraisal of the Current Safety Evidence

Overall, the available evidence indicates a generally adequate short- to medium-term tolerability profile for standardized hyperforin-containing *H. perforatum* preparations, with most reported adverse events being mild and reversible and serious direct toxic effects uncommon. However, this evidence should not be interpreted as establishing the safety of purified hyperforin. Most clinical safety data derive from multicomponent botanical preparations, while compound-specific information on purified hyperforin—particularly during prolonged exposure or with formulations designed to increase systemic bioavailability—remains limited.

Importantly, the principal clinical safety concern appears to arise less from intrinsic toxicity than from pharmacokinetic interaction liability. Hyperforin-mediated activation of PXR and subsequent induction of CYP3A4 and P-glycoprotein provides a strong mechanistic basis for the clinically important pharmacokinetic interactions observed with hyperforin-containing *H. perforatum* preparations. However, because most clinical interaction data derive from multicomponent preparations rather than purified hyperforin, the magnitude of clinical interaction attributable specifically to hyperforin remains incompletely defined. This risk is especially relevant in patients receiving drugs with narrow therapeutic indices, individuals with multimorbidity or polypharmacy, and clinical settings requiring prolonged treatment. Thus, conventional adverse-event frequency alone is insufficient to characterize the overall safety profile of hyperforin. Accordingly, drug–herb interactions currently represent among the major practical safety limitations to the clinical use of hyperforin-containing preparations.

Safety interpretation is further complicated by variability in hyperforin content, formulation, administered dose, duration of exposure, patient characteristics, and concomitant pharmacotherapy. Accordingly, the current evidence supports favorable intrinsic tolerability under selected conditions but not an unequivocally low-risk safety profile. Future studies should prioritize long-term evaluation of chemically characterized purified hyperforin, exposure–toxicity relationships, standardized and highly bioavailable formulations, vulnerable populations, and systematic assessment of clinically relevant drug interactions. Such evidence will be essential for defining a reliable therapeutic window and determining whether purified hyperforin can be developed safely as a pharmaceutical intervention.

Table 10 integrates these findings according to evidence source, preparation evaluated, principal safety finding, and clinical significance, thereby highlighting both the relative strengths of the available safety evidence and the areas in which uncertainty remains greatest.

## 14. Current Limitations, Knowledge Gaps, and Barriers to Clinical Translation

### 14.1. Overview

Despite extensive investigation of its pharmacological properties, hyperforin has not yet emerged as a clinically validated therapeutic agent. This translational gap reflects several interconnected limitations, including the predominance of mechanistic and preclinical evidence, limited compound-specific human data, heterogeneity among hyperforin-containing *H. perforatum* preparations, physicochemical and pharmacokinetic constraints, clinically relevant interaction liability, and challenges in pharmaceutical development and regulatory standardization. Importantly, the maturity of evidence varies substantially across therapeutic areas: depression has the strongest human support, although predominantly from standardized hyperforin-containing *H. perforatum* preparations, whereas most other proposed applications remain largely preclinical.

These barriers are closely interrelated, as variability in compound and formulation characteristics influences systemic exposure, which in turn affects target engagement, efficacy, safety, and interpretation of clinical findings. The following subsections examine the principal factors that currently limit the development of hyperforin as a chemically defined and clinically validated therapeutic intervention.

### 14.2. Preclinical–Clinical Translation Gap

A major limitation in the development of hyperforin is the marked imbalance between extensive mechanistic and preclinical evidence and comparatively limited compound-specific clinical validation. Most proposed therapeutic effects have been demonstrated in cellular or animal models, which are valuable for identifying biological mechanisms but do not fully reproduce human disease complexity, pharmacokinetics, comorbidities, or treatment responses. Moreover, experimental concentrations and exposure conditions may not always be clinically attainable, and modulation of molecular or biochemical endpoints does not necessarily translate into meaningful patient benefit.

An additional challenge is the compound–preparation attribution gap. Whereas mechanistic studies frequently investigate purified hyperforin, most human trials have evaluated standardized *H. perforatum* extracts containing hyperforin together with hypericin, flavonoids, and other bioactive constituents. Consequently, clinical outcomes observed with these preparations cannot be attributed specifically to hyperforin, and possible additive or synergistic effects among constituents cannot be excluded.

This distinction is particularly important because the relatively mature clinical evidence for depression does not constitute equivalent evidence for purified hyperforin, while most other proposed applications remain supported predominantly by preclinical or mechanistic findings. Thus, establishing compound-specific efficacy, clinically achievable exposure, and target engagement represents a critical prerequisite for translating the extensive experimental literature on hyperforin into clinically validated therapeutic applications.

### 14.3. Standardization and Formulation Variability

A major barrier to the clinical translation of hyperforin is the substantial variability in its content and stability across *H. perforatum* preparations. Differences in botanical source, extraction and manufacturing procedures, storage conditions, and formulation technologies can markedly influence hyperforin concentration, purity, stability, and biological activity, thereby contributing to variability in pharmacokinetic exposure, efficacy, and safety [31,32,423,424,425,426,427,428,429,430,431].

This problem is compounded by the physicochemical instability of hyperforin, which is susceptible to degradation during extraction, purification, storage, and formulation, particularly following exposure to light, oxygen, heat, and moisture [31,35,45,432,433,434]. Consequently, nominally similar preparations may deliver substantially different amounts of intact and bioavailable hyperforin, complicating comparisons across experimental and clinical studies.

Insufficient analytical and manufacturing standardization therefore represents a major obstacle to reproducibility and to the establishment of reliable dose–exposure–response relationships [32,435]. Future development should prioritize chemically characterized preparations with defined hyperforin content, validated stability, and reproducible formulation characteristics to enable meaningful comparison across studies and support subsequent pharmacokinetic and clinical evaluation.

### 14.4. Pharmacokinetic and Bioavailability Limitations

The clinical translation of hyperforin is constrained by unfavorable physicochemical and pharmacokinetic properties. Its high lipophilicity, poor aqueous solubility, chemical instability, and formulation-dependent absorption contribute to variable systemic exposure and complicate the establishment of reproducible dose–exposure relationships [31,39,40,433,434,436,437].

Following absorption, hyperforin undergoes extensive metabolism involving CYP3A4 and CYP2C enzymes, while systemic exposure may vary substantially according to formulation characteristics, dose, metabolic phenotype, and other patient-related factors [39,362,436,437]. These sources of variability complicate extrapolation from administered dose to circulating and tissue concentrations and may partly account for inconsistencies among experimental and clinical findings.

A major translational challenge is therefore the exposure–translation gap between experimentally active concentrations and those reproducibly achievable in humans. Robust pharmacokinetic–pharmacodynamic characterization is needed to establish the sequence from administered dose to circulating concentration, tissue exposure, target engagement, and biological or clinical response [39,40,362,436,437]. This issue is further complicated by hyperforin-mediated induction of drug-metabolizing enzymes and transporters, which can alter its own pharmacokinetic context as well as that of concomitant medications [362,436,437].

Overall, the combination of physicochemical instability, variable absorption and metabolism, and interaction liability limits predictable systemic exposure and represents a major obstacle to defining clinically relevant dosing regimens. Addressing these limitations will require standardized formulations and rigorous pharmacokinetic studies capable of linking hyperforin exposure to target engagement and therapeutic response [31,39,433,434,436,437].

### 14.5. Sources of Inconsistency Across the Evidence Base

The apparent inconsistencies across the hyperforin literature should not necessarily be interpreted as contradictory biological effects, because the studies frequently differ at several levels simultaneously. At the intervention level, purified hyperforin, hyperforin-rich fractions, standardized *H. perforatum* extracts, and heterogeneous commercial preparations are often considered within the same broad evidence base despite substantial differences in chemical composition and delivered hyperforin dose. At the experimental level, studies differ in model system, concentration, treatment duration, route of administration, and endpoints. At the clinical level, variability in disease severity, diagnostic criteria, extract composition, dose, treatment duration, comparator, concomitant therapies, and outcome measures further limits direct comparison. Pharmacokinetic variability provides an additional source of heterogeneity because differences in formulation, chemical stability, absorption, metabolism, and drug–transporter interactions may produce substantially different systemic and tissue exposures even when nominal administered doses appear similar. Consequently, discordant findings should be interpreted within the context of intervention identity, achievable exposure, methodological quality, and level of evidence rather than considered interchangeable evidence for or against hyperforin efficacy.

### 14.6. Limited Long-Term Clinical Evidence and Remaining Mechanistic Uncertainties

Long-term clinical evidence for hyperforin remains limited. Even in depression, where human evidence is comparatively mature, most trials have been of relatively short duration and have evaluated standardized *H. perforatum* preparations rather than purified hyperforin. For other proposed chronic indications, long-term disease-specific evidence is sparse, limiting conclusions regarding sustained efficacy, cumulative safety, and the consequences of prolonged exposure.

Important mechanistic uncertainties also remain despite the extensive experimental literature. Hyperforin influences multiple interconnected pathways, including TRPC6-dependent signaling, neurotransmission and neurotrophic responses, inflammatory and redox regulation, mitochondrial function, apoptosis, autophagy, and metabolic homeostasis. However, the relative contribution and hierarchical organization of these mechanisms remain incompletely defined. In particular, it is unclear whether TRPC6 activation represents a dominant upstream mechanism across biological contexts or whether hyperforin acts through context-dependent multitarget effects involving several parallel pathways.

The biological response to hyperforin may also vary according to tissue, dose, disease state, and individual characteristics, while pharmacogenomic, epigenetic, microRNA-mediated, microbiota-related, metabolomic, and broader network-level influences remain insufficiently characterized. These factors may contribute to heterogeneity among experimental and clinical findings and complicate identification of primary molecular actions versus secondary downstream responses.

Overall, the broad pharmacological activity of hyperforin provides substantial therapeutic potential but also complicates mechanistic interpretation and clinical translation. Future studies should therefore prioritize clinically relevant exposure conditions and establish causal links among hyperforin exposure, primary target engagement, downstream biological responses, and clinically meaningful outcomes.

### 14.7. Integrated Appraisal of Evidence Maturity and Translational Readiness

The therapeutic evidence for hyperforin is characterized by a marked imbalance between extensive mechanistic and preclinical investigation and comparatively limited compound-specific clinical validation. Translational maturity also differs substantially across therapeutic domains. Depression and mood disorders represent the most clinically developed indication, supported by randomized trials and evidence syntheses of standardized hyperforin-containing *H. perforatum* preparations; however, these findings do not establish the independent clinical efficacy of purified hyperforin. Anxiety and selected dermatological applications have emerging but substantially less developed human evidence, whereas neurodegenerative, cardiometabolic, inflammatory, oncological, infectious, gastrointestinal, musculoskeletal, and healthy-aging applications remain predominantly preclinical.

**Figure 4:**
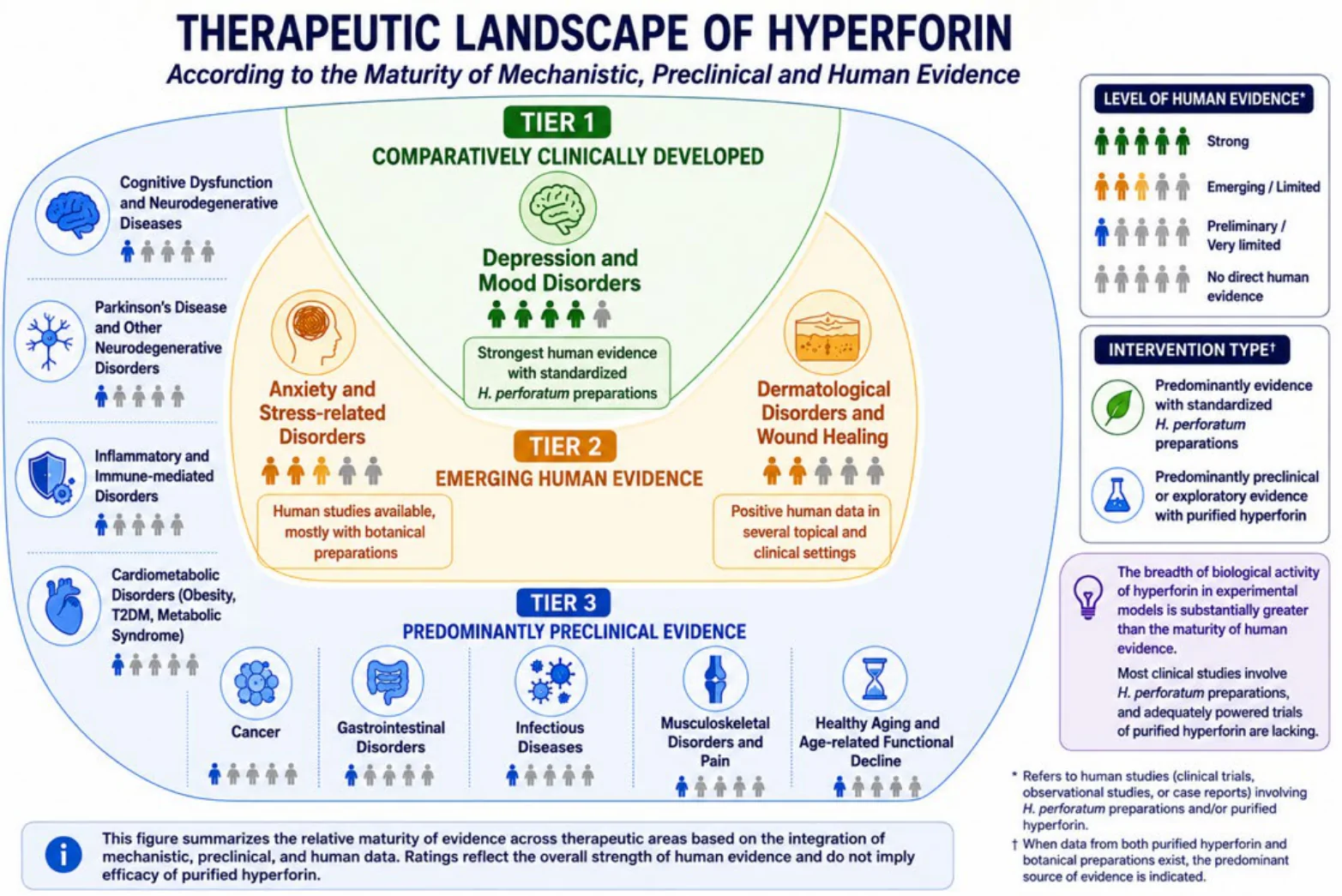
Therapeutic landscape of hyperforin according to the maturity of mechanistic, preclinical, and human evidence. Therapeutic areas are grouped according to their relative translational maturity, ranging from predominantly preclinical evidence to emerging and comparatively developed human evidence. Depression and mood disorders represent the most clinically investigated domain, although the available human evidence derives predominantly from standardized hyperforin-containing *H. perforatum* preparations rather than purified hyperforin. The classification is intended as a qualitative synthesis of the evidence reviewed and should not be interpreted as a formal certainty-of-evidence grading or as confirmation of clinical efficacy of purified hyperforin.

This heterogeneity in translational maturity is summarized in Figure 4. Importantly, the classification reflects the relative maturity of the overall evidence base rather than established clinical efficacy of purified hyperforin. Mechanistic plausibility or reproducible activity in cellular and animal models should not be considered equivalent to therapeutic efficacy, particularly when the concentrations, formulations, or routes of administration used experimentally may not correspond to clinically achievable human exposure. Accordingly, the translational significance of each therapeutic domain depends on the convergence of disease-specific mechanistic evidence, reproducible in vivo efficacy, pharmacokinetic feasibility, intervention specificity, and supportive human evidence rather than on the breadth of reported biological activities alone.

Overall, the evidence therefore identifies a substantial translational gap: hyperforin is a pharmacologically pleiotropic and extensively investigated natural compound, but its development as a chemically defined therapeutic agent remains immature. In particular, clinical evidence obtained with multicomponent *H. perforatum* preparations must be distinguished from direct evidence for purified hyperforin, for which adequately powered clinical validation remains lacking. The interacting factors responsible for this discrepancy are integrated in Section 14.7.

A more detailed qualitative comparison of mechanistic, animal, and human evidence across the principal therapeutic domains is provided in Table 11. The ratings represent a structured qualitative appraisal within the framework of this narrative review and should not be interpreted as formal certainty-of-evidence grades or as substitutes for validated systems such as GRADE. Ratings were based on predefined dimensions including study quantity; consistency and reproducibility; methodological rigor and disease relevance; disease and intervention specificity; availability of disease-specific in vivo evidence; availability and methodological quality of human studies; concordance across mechanistic, animal, and human evidence; and translational relevance, including pharmacokinetic feasibility and reproducibility of exposure. Higher ratings therefore indicate a broader, more consistent, and more translationally informative evidence base rather than simply a greater number of publications.

The qualitative evidence ratings were assigned collectively by all authors following a comprehensive evaluation of the available mechanistic, preclinical, translational, and clinical literature. Final ratings for each therapeutic domain were determined after detailed discussion of the predefined evidence dimensions and were reached by consensus among all authors.

### 14.8. Why Has Hyperforin Not Yet Achieved Clinical Translation?

Despite more than two decades of pharmacological investigation and a substantial body of mechanistic and preclinical evidence, hyperforin has not progressed to a clinically validated therapeutic agent. This discrepancy is best understood not as a failure of biological activity, but as the consequence of a multistep translational bottleneck in which compound attribution, pharmaceutical development, human exposure, safety, clinical validation, and regulatory feasibility remain insufficiently connected.

**Figure 5:**
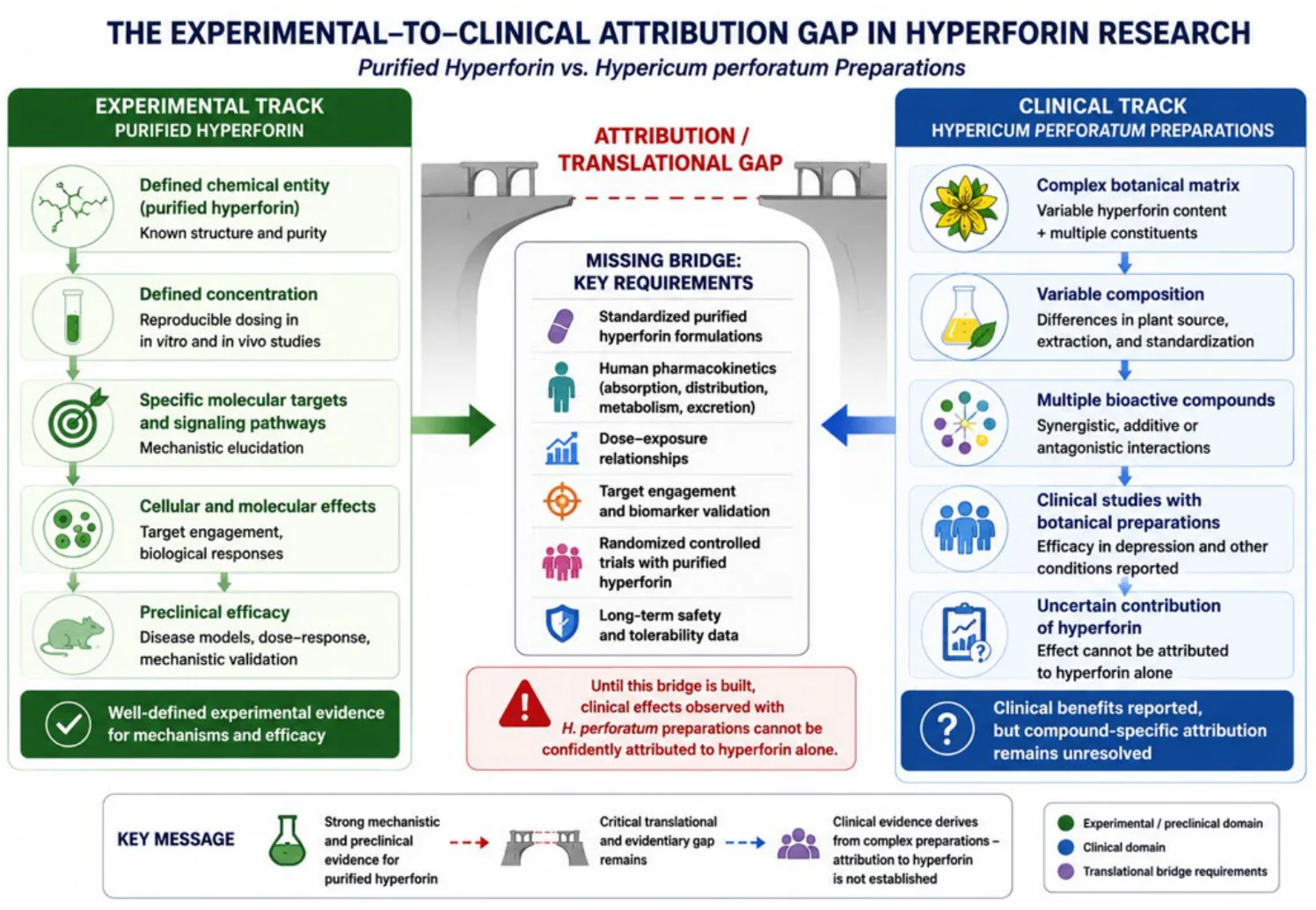
Experimental-to-clinical attribution gap in hyperforin research. Experimental and preclinical investigations frequently evaluate purified hyperforin under chemically defined conditions, enabling compound-specific mechanistic assessment. In contrast, most available clinical evidence derives from multicomponent *H. perforatum* preparations, in which variability in composition and the presence of additional bioactive constituents complicate attribution of clinical effects specifically to hyperforin. Bridging this gap requires standardized purified-hyperforin formulations, characterization of human pharmacokinetics and dose–exposure relationships, demonstration of target engagement, adequately powered compound-specific randomized controlled trials, and comprehensive long-term safety assessment.

The first and most fundamental problem is the disconnect between the compound evaluated experimentally and the interventions assessed clinically. Mechanistic studies frequently investigate purified hyperforin under chemically defined conditions, whereas most human studies have evaluated multicomponent *H. perforatum* preparations. Consequently, clinical effects observed with these botanical products cannot be attributed unequivocally to hyperforin, while the independent efficacy, therapeutic dose, exposure–response relationship, and therapeutic window of purified hyperforin remain largely undefined. This experimental-to-clinical attribution gap is illustrated in Figure 5 and represents the principal obstacle to establishing hyperforin as a distinct therapeutic entity.

A second interconnected barrier concerns the transition from biological activity to reproducible human exposure. Hyperforin combines poor aqueous solubility, chemical instability, variable oral bioavailability, and formulation-dependent pharmacokinetics. Thus, even where experimentally relevant mechanisms are well characterized, it remains uncertain whether comparable and sustained target exposure can be achieved safely in humans. Importantly, pharmaceutical strategies designed to increase systemic exposure must also confront a countervailing safety problem: hyperforin activates PXR and induces CYP3A4 and P-glycoprotein, creating clinically relevant interaction potential. Increasing bioavailability may therefore improve pharmacological exposure while simultaneously increasing interaction liability, particularly in patients with chronic diseases who commonly receive multiple medications.

These limitations create a further clinical-development problem. Without a standardized purified formulation and predictable human pharmacokinetics, it is difficult to define rational dose ranges, demonstrate pharmacodynamic target engagement, select clinically relevant biomarkers, or design adequately powered compound-specific randomized trials. The translational pathway therefore remains incomplete at several sequential stages: chemical standardization → reproducible exposure → target engagement → efficacy → long-term safety and interaction assessment. Failure to establish one stage weakens the interpretability of the next, helping explain why extensive mechanistic evidence has not yet generated corresponding compound-specific clinical validation.

Hyperforin also occupies an unusual regulatory and commercial position between a botanical constituent and a conventional drug candidate. Botanical preparations are intrinsically more difficult to standardize because of variability in phytochemical composition, whereas development of purified hyperforin according to conventional pharmaceutical standards would require extensive formulation optimization, toxicological characterization, pharmacokinetic and dose-ranging studies, and phase II–III clinical evaluation. Such development demands considerable resources, while the commercial incentives may be comparatively limited for a naturally occurring compound.

Taken together, these interconnected barriers explain why hyperforin remains pharmacologically compelling but clinically underdeveloped. The central challenge is therefore no longer to identify additional biological activities, but to establish whether a chemically defined and pharmaceutically optimized hyperforin intervention can achieve reproducible human exposure, demonstrable target engagement, acceptable safety and interaction liability, and clinically meaningful efficacy. Addressing these sequential requirements provides the basis for the research priorities and translational development strategy outlined in Section 15.

## 15. Future Directions and Research Priorities

### 15.1. Translational Priorities

Future hyperforin development should follow a sequential translational strategy prioritizing: chemically defined intervention identity and formulation; reproducible human exposure; pharmacodynamic target engagement; safety and drug-interaction characterization; and subsequently compound-specific efficacy trials. Studies should explicitly distinguish purified hyperforin from *H. perforatum* preparations and report purity/content, dose, formulation, stability, and relevant pharmaceutical characteristics. Therapeutic development should be prioritized according to disease-specific evidence maturity, in vivo reproducibility, pharmacokinetic feasibility, preliminary human support, and anticipated benefit–risk rather than pursued simultaneously across all proposed indications. The qualitative hierarchy presented in Table 11 can therefore guide prioritization for compound-specific clinical development.

**Figure 6:**
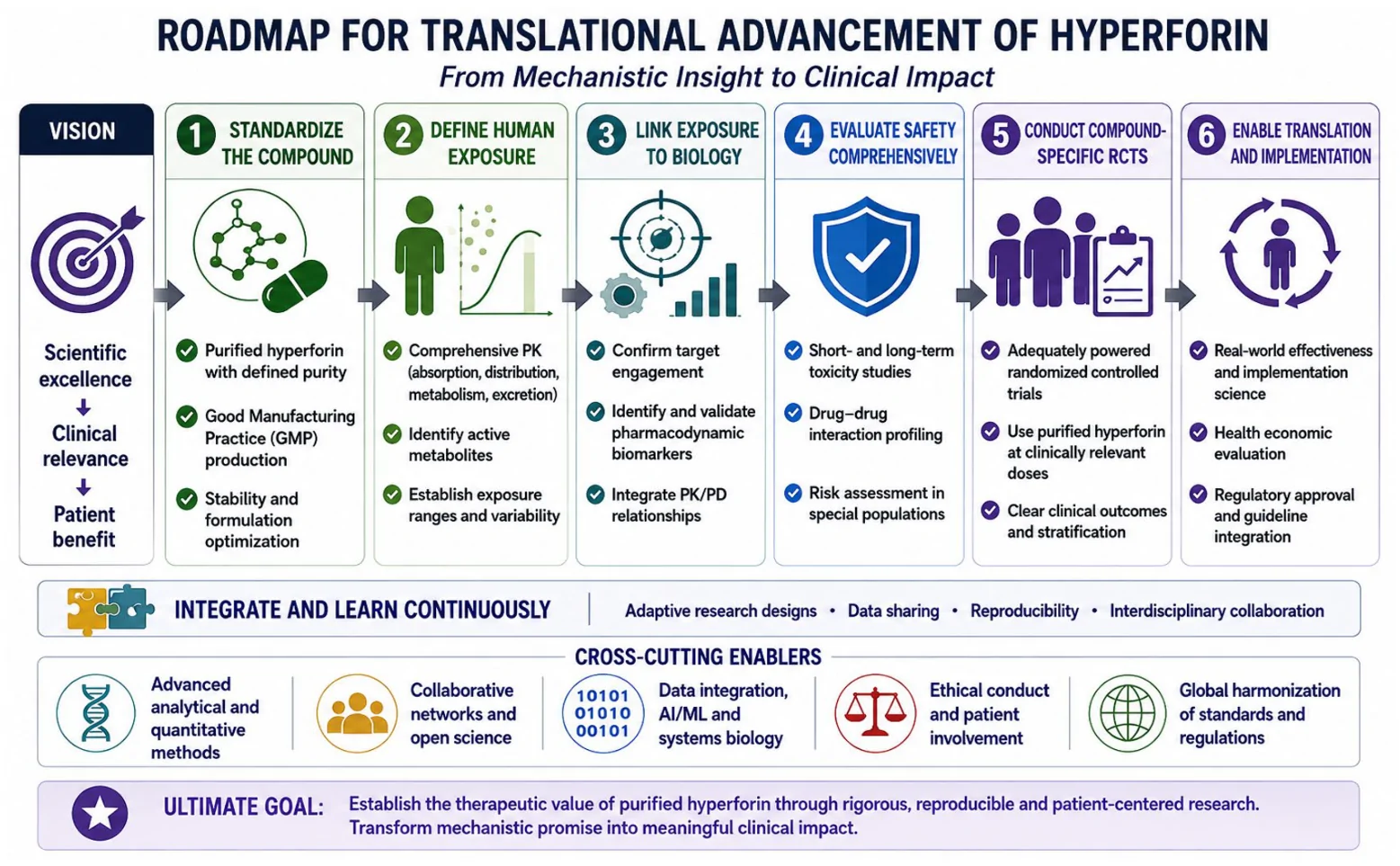
Proposed roadmap for the translational development of hyperforin from mechanistic evidence to clinical implementation. The framework prioritizes sequential development through compound standardization and formulation optimization, characterization of human pharmacokinetics and exposure variability, demonstration of pharmacodynamic target engagement, comprehensive safety and drug-interaction assessment, and adequately powered randomized controlled trials using chemically defined hyperforin interventions. Later-stage implementation would require confirmation of clinical effectiveness, regulatory evaluation, and integration into appropriate therapeutic settings. Cross-cutting priorities include methodological rigor, reproducibility, interdisciplinary collaboration, data integration, ethical conduct, and harmonization of analytical and regulatory standards.

Figure 6 summarizes the proposed stepwise pathway from chemical standardization and formulation optimization through human pharmacokinetics, target engagement, safety and interaction assessment, proof-of-concept studies, and ultimately adequately powered randomized trials and regulatory evaluation. Progression to each stage should depend on satisfactory evidence from the preceding stage.

### 15.2. Mechanistic Validation and Biomarker-Guided Development

A central priority for hyperforin development is to distinguish primary pharmacological actions from secondary or context-dependent responses. Although experimental evidence implicates TRPC6-mediated Ca^2+^ signaling and multiple neurobiological, mitochondrial, inflammatory, redox, autophagic, apoptotic, and metabolic pathways, their causal and temporal hierarchy remains incompletely defined. Future studies should therefore move beyond descriptive pathway associations by using target-specific pharmacological inhibition, genetic manipulation, dose–response approaches, and tissue- or cell-specific models to establish causal relationships and identify mechanisms most relevant to disease-specific therapeutic effects.

Systems-biology and integrated multi-omics approaches may complement targeted mechanistic studies by characterizing tissue-specific molecular responses, efficacy or toxicity signatures, and candidate biomarkers associated with hyperforin exposure and responsiveness [438,439,440,441,442,443]. Such approaches may also help distinguish primary pharmacological effects from downstream network responses and identify sources of interindividual variability. However, findings from high-dimensional datasets require targeted experimental and independent validation before translational or clinical application.

In parallel, development should prioritize indication-specific pharmacodynamic biomarkers capable of linking hyperforin exposure to biological target engagement. Candidate panels may encompass inflammatory and redox, neurotrophic, mitochondrial, metabolic, immune, genomic/epigenetic, and microbiome-related measures, selected according to the mechanism most strongly supported for the therapeutic indication [444,445,446,447]. Their primary translational value is to determine whether biologically meaningful target modulation occurs at clinically achievable hyperforin exposures, thereby linking pharmacokinetics with biological response and clinical outcomes.

Validated biomarkers could subsequently support dose selection, confirmation of target engagement, identification of responder subgroups, patient enrichment, and evidence-based go/no-go decisions during early clinical development [444,445]. Integration of pharmacodynamic and multi-omics biomarkers may further facilitate patient stratification and adaptive dose optimization [444,445,446,447]. Nevertheless, biomarker-guided strategies should complement rather than replace clinically meaningful outcomes and adequately powered randomized evidence.

### 15.3. Pharmaceutical Optimization and Human Pharmacokinetics

Development of a chemically defined, stable, reproducible, and clinically suitable purified-hyperforin formulation is a prerequisite for compound-specific therapeutic evaluation. Pharmaceutical optimization should prioritize protection against chemical degradation, reproducible drug content and release, and predictable absorption using appropriate delivery technologies, including lipid-, polymer-, or cyclodextrin-based systems. However, formulation performance should be judged by demonstrated pharmaceutical and pharmacokinetic advantages rather than improvements in solubility or in vitro release alone.

Promising formulations should undergo standardized physicochemical and controlled human pharmacokinetic evaluation to characterize stability, bioavailability, systemic exposure, dose proportionality, elimination, interindividual variability, and, where relevant, food effects and tissue distribution. Critically, these studies should determine whether exposures associated with experimental pharmacological activity are achievable and sustainable at clinically acceptable doses.

Pharmacokinetic development should be integrated with the pharmacodynamic strategy described in Section 15.2. Early clinical studies should establish exposure–target engagement and exposure–response relationships using validated, indication-specific biomarkers rather than considering systemic exposure as an isolated endpoint. This approach would clarify whether increasing exposure produces biologically meaningful target modulation and provide a rational basis for dose selection and progression to proof-of-concept trials.

Particular attention should be given to the interaction liability associated with PXR activation and induction of CYP3A4 and P-glycoprotein. Improved bioavailability should not automatically be considered advantageous, because greater systemic exposure may also increase clinically relevant drug interactions. Formulation and dose-ranging studies should therefore incorporate exposure-dependent assessment of enzyme and transporter induction, with dedicated clinical interaction studies where appropriate, particularly for chronic indications and populations with multimorbidity or polypharmacy.

### 15.4. Clinical Trial Development and Precision Approaches

Following pharmaceutical optimization, human pharmacokinetic characterization, demonstration of target engagement, and initial safety assessment, clinical development should proceed through a sequential, indication-specific framework. Early-phase studies of chemically characterized purified hyperforin should establish tolerability, dose–exposure relationships, pharmacodynamic activity, interaction liability, and the dose range required for biologically meaningful target engagement before progression to proof-of-concept efficacy trials.

Subsequent proof-of-concept studies and RCTs should prioritize indications according to the evidence-maturity framework presented in Section 14.6 and Table 11. Trials should be adequately powered and indication-specific, with appropriate comparators, clinically meaningful predefined outcomes, standardized safety and drug-interaction monitoring, and suitable follow-up. Where compound-specific efficacy is being evaluated, purified hyperforin or a chemically defined hyperforin formulation should be used. Studies of *H. perforatum* preparations remain clinically relevant but require detailed phytochemical characterization, and their therapeutic effects should not be attributed exclusively to hyperforin.

Precision approaches may complement this pathway when supported by a validated biological rationale. Pharmacogenomic variation and other patient-level biological characteristics, including inflammatory, metabolic, neurobiological, and microbiome-related factors, may contribute to interindividual variability in hyperforin exposure, target engagement, safety, and therapeutic response [448,449,450,451]. Prospective evaluation of these determinants could help characterize response heterogeneity and inform subsequent patient stratification.

Integration of pharmacogenomic information with validated pharmacodynamic biomarkers and multi-omics profiles may further support biomarker-guided stratification, patient enrichment, individualized dose selection, and adaptive early-development decisions [443,447,448,449,450,451]. However, such approaches should remain hypothesis-driven and undergo prospective validation, complementing rather than replacing clinically meaningful endpoints and adequately powered randomized evidence.

Combination therapy with conventional pharmacological agents may represent an additional strategy in selected indications but should be considered only after the pharmacokinetic and interaction profile of purified hyperforin has been adequately characterized. Given its PXR-mediated interaction liability, potential combinations should undergo appropriate pharmacokinetic and interaction assessment to determine whether any pharmacodynamic benefit is accompanied by clinically relevant alterations in co-administered drug exposure.

Overall, clinical development should follow a stage-gated approach, with progression to larger randomized trials contingent on reproducible human exposure, demonstrable target engagement, acceptable safety and interaction liability, and preliminary therapeutic efficacy. This strategy would concentrate resources on the indications, formulations, doses, and patient populations with the greatest likelihood of clinically meaningful benefit.

### 15.5. Standardization, Regulation, and Clinical Implementation

Successful translation of hyperforin into clinical practice will require regulatory and quality-control frameworks that clearly distinguish purified hyperforin as a chemically defined therapeutic candidate from hyperforin-containing *H. perforatum* preparations. Because botanical medicines, phytopharmaceuticals, nutraceuticals, and dietary supplements are regulated differently across jurisdictions, development programs should define the intended product category and apply appropriate quality, manufacturing, safety, and efficacy requirements. Purified hyperforin intended for therapeutic use should increasingly be developed according to conventional pharmaceutical principles.

Reproducible product quality is a central requirement. For purified hyperforin, standardization should incorporate validated analytical characterization, predefined purity and stability specifications, assessment of degradation products, appropriate storage conditions, and transparent formulation reporting. Hyperforin-containing botanical preparations should similarly undergo quantitative characterization of hyperforin and other relevant bioactive constituents, together with harmonized manufacturing and batch-to-batch quality assessment. These measures are necessary to ensure that interventions evaluated across preclinical and clinical studies are sufficiently characterized and comparable.

Regulatory development of purified hyperforin should integrate chemistry, manufacturing, and quality control with pharmacokinetic, pharmacodynamic, toxicological, and clinical evidence. Product specifications and formulation characteristics should be linked to reproducible human exposure, while dose selection should be guided by pharmacokinetic and target-engagement data rather than nominal administered dose alone. Long-term safety and drug-interaction liability, particularly those associated with PXR-dependent induction of drug-metabolizing enzymes and transporters, should be incorporated prospectively throughout development.

Clinical implementation would additionally require appropriate pharmacovigilance and risk-management strategies, particularly for patients receiving concomitant medications or experiencing polypharmacy, prolonged treatment, or higher formulation-specific systemic exposure. Clinically relevant interactions should be reflected in appropriate labeling, contraindications, precautions, and monitoring recommendations.

Progress toward regulatory acceptance would also benefit from coordinated collaboration among research, clinical, industry, and regulatory stakeholders. Harmonization of intervention characterization, analytical and pharmacokinetic methods, safety assessment, and clinical outcome definitions would improve comparability across studies and reduce fragmentation of the evidence base.

Ultimately, regulatory and clinical progression should be evidence-gated rather than activity-driven, requiring convergence of reproducible pharmaceutical quality, predictable human exposure, validated target engagement, clinically meaningful efficacy, and an acceptable long-term safety and interaction profile. Only when these requirements are demonstrated for a clearly defined hyperforin intervention can its extensive experimental pharmacology be meaningfully translated into a clinically validated therapeutic agent.

## 16. Conclusions

Hyperforin is a pharmacologically pleiotropic natural compound with a broad spectrum of biological activities supported by extensive mechanistic, cellular, and animal evidence. Its ability to modulate interconnected processes involving neurotransmission, neuroplasticity, inflammation, oxidative stress, mitochondrial homeostasis, immune regulation, cell survival, and metabolic signaling provides a biologically plausible basis for its potential relevance across multiple disease states. Nevertheless, the breadth of these experimentally demonstrated effects should not be interpreted as equivalent to therapeutic efficacy, and the maturity of the available evidence differs substantially among proposed clinical applications.

A clear evidence hierarchy emerges from the current literature. Depression and mood disorders represent the most clinically developed therapeutic domain, with randomized trials and evidence syntheses supporting standardized hyperforin-containing *H. perforatum* preparations for mild-to-moderate depressive disorders. However, because these preparations contain multiple pharmacologically active constituents, their clinical effects cannot establish the independent therapeutic contribution of hyperforin. For most other proposed applications—including neurodegenerative, inflammatory and immune-mediated, cardiometabolic, oncological, antimicrobial, gastrointestinal, dermatological, musculoskeletal, and healthy-aging indications—the evidence remains substantially less mature and is predominantly mechanistic or preclinical. Robust compound-specific clinical evidence for purified hyperforin therefore remains lacking.

The central translational challenge is no longer simply to identify additional molecular pathways or biological activities influenced by hyperforin, but to determine whether its extensive experimental pharmacology can be converted into a chemically defined, pharmacologically reproducible, safe, and clinically effective intervention. This transition remains constrained by chemical instability, formulation-dependent exposure, limited compound-specific human pharmacokinetic information, uncertainty regarding target engagement at clinically achievable concentrations, and clinically relevant interaction liability, particularly through PXR-dependent regulation of drug-metabolizing enzymes and transporters.

Accordingly, hyperforin should currently be regarded as a promising but clinically underdeveloped pharmacological candidate rather than an established therapeutic agent. Its future therapeutic relevance will ultimately depend on whether a standardized and chemically characterized intervention can achieve reproducible human exposure, demonstrable target engagement, an acceptable long-term safety and drug-interaction profile, and clinically meaningful efficacy. Establishing these requirements will determine whether the substantial experimental promise of hyperforin can ultimately be translated into evidence-based therapeutic application.

## Figures and Tables

**Table 1:** Principal mechanisms of action of hyperforin and associated biological targets and regulatory systems *.

Mechanistic Domain	Principal Biological Targets/Regulatory Systems	Reported Action of Hyperforin	Principal Biological Consequences	References
**Neurotransmitter regulation**	Serotonergic, dopaminergic, noradrenergic, GABAergic and glutamatergic systems; intracellular Na^+^/Ca^2+^ homeostasis	Modulation of neurotransmitter uptake through alteration of transmembrane ion gradients rather than conventional competitive transporter inhibition	↑ extracellular monoamine availability; modulation of excitatory/inhibitory neurotransmission; support of mood regulation, stress adaptation and synaptic function	[14,51,52,53,54,55]
**TRPC6-mediated Ca^2+^ signaling**	TRPC6; Ca^2+^; CaMKII; CREB; ERK1/2; PI3K/Akt; BDNF	Activation of TRPC6-mediated Ca^2+^ influx and downstream neurotrophic signaling	↑ neuronal differentiation, dendritic arborization, synaptic maturation and plasticity; ↑ neuronal resilience	[14,55,56,57,58,59,60]
**Inflammatory signaling**	NF-κB; MAPK/ERK/JNK/p38; TNF-α; IL-1β; IL-6; COX-2/prostaglandins	Suppression/modulation of pro-inflammatory transcriptional and kinase signaling and inflammatory mediator production	↓ inflammatory signaling and cytokine production; attenuation of chronic inflammatory responses	[5,14,51,60,61,62,63]
**Redox regulation and antioxidant defense**	ROS; SOD; catalase; glutathione peroxidase; glutathione redox system; mitochondria	↓ intracellular ROS and oxidative damage; ↑ endogenous antioxidant defenses; preservation of mitochondrial integrity	Restoration of redox homeostasis; ↓ oxidative macromolecular damage; increased cellular resilience to oxidative stress	[61,64,65,66,67]
**Apoptotic regulation**	Bcl-2/Bax balance; mitochondrial membrane; cytochrome c; caspase-9/-3; death-receptor pathways	Context-dependent regulation of intrinsic and extrinsic apoptosis	Protection from pathological apoptosis in stressed non-malignant cells; promotion of apoptotic signaling in transformed/malignant cells	[66,67,68,69,70,71,72,73,74,75,76]
**Autophagy and proteostasis**	PI3K/Akt/mTOR; AMPK; autophagic machinery	Modulation of autophagic signaling and autophagic flux	Clearance of damaged cellular components; maintenance of proteostasis, stress adaptation and intracellular quality control	[55,67,77,78,79]
**Transcriptional regulation**	NF-κB; CREB; AP-1; Nrf2; STAT proteins	Modulation of transcription-factor activity and downstream gene-expression programs	Coordinated regulation of inflammatory, redox, neuroplastic, apoptotic and metabolic responses	[55,59,67]
**Epigenetic/post-transcriptional regulation**	Histone modifications; chromatin remodeling; DNA accessibility; microRNAs	Potential modulation of epigenetic and post-transcriptional regulatory processes	Longer-term regulation of gene-expression programs relevant to inflammation, neurodegeneration, carcinogenesis and metabolic homeostasis	[65,67,77,80,81]
**Mitochondrial bioenergetics**	Mitochondrial membrane potential; electron transport chain; oxidative phosphorylation; ATP production	Preservation of mitochondrial function and bioenergetic efficiency; ↓ mitochondrial oxidative stress	↑ cellular energy availability; maintenance of mitochondrial integrity and metabolic resilience	[67,82,83]
**Energy-sensing and metabolic signaling**	AMPK; PI3K/Akt; mitochondrial-biogenesis pathways	Modulation of energy-sensing and nutrient-utilization pathways	↑ metabolic flexibility and adaptation to energetic stress; maintenance of cellular energy homeostasis	[67,84]

* Note: The mechanisms summarized in this table primarily reflect experimental evidence obtained using purified hyperforin in in vitro systems, with supportive in vivo evidence available for selected pathways. Several signaling pathways listed, including NF-κB, MAPK, PI3K/Akt, and AMPK, should be regarded predominantly as downstream regulatory systems rather than established direct molecular targets of hyperforin. TRPC6 currently represents one of the most consistently supported proximal mechanistic targets. Evidence derived from hyperforin-containing *H. perforatum* preparations should be interpreted cautiously because the contribution of other phytochemical constituents cannot be excluded. The table summarizes reported biological mechanisms and does not imply that all listed pathways are engaged at clinically achievable human exposure. For several mechanisms, the concentration–exposure relationship and target-tissue pharmacokinetics remain insufficiently defined.

**Table 2:** Key preclinical evidence for the neuroprotective and neuropsychiatric effects of purified hyperforin and hyperforin-containing preparations *.

Disease/Functional Domain	Main Preclinical Models	Type of Intervention	Key Mechanisms	Most Important Findings	References
**Depression and mood disorders**	Rodent chronic-stress models; forced-swimming and tail-suspension tests	Purified hyperforin and hyperforin-containing preparations	Monoaminergic regulation; TRPC6; BDNF/neuroplasticity; neurogenesis; anti-inflammatory, antioxidant and mitochondrial effects	↓ depressive-like behavior and immobility; normalization of stress-related monoaminergic alterations; ↑ hippocampal BDNF and neurogenesis; improved synaptic plasticity; ↓ neuroinflammation and oxidative damage	[54,91,95,96,97,98,99,100]
**Anxiety and stress-related disorders**	Rodent anxiety and chronic-stress models	Purified hyperforin and hyperforin-containing preparations	Serotonergic, GABAergic and glutamatergic regulation; stress-response modulation; anti-inflammatory and antioxidant actions	↓ anxiety-like behavior; improved stress resilience; normalization of stress-induced neurochemical changes; ↓ inflammatory and oxidative responses	[54,93,95,97,98,99,111,112,113,114]
**Cognitive function, learning and memory**	Learning/memory and aging-related animal models; complementary neuronal models	Purified hyperforin and hyperforin-containing preparations	TRPC6/BDNF-associated signaling; neurotransmission; synaptic plasticity; neuroprotection	Improved spatial learning and memory retention; enhanced neuronal connectivity and cognitive performance; protection against oxidative and inflammatory injury	[52,54,120,121,122,123,124]
**Synaptic plasticity and neurogenesis**	Neuronal cultures; hippocampal/cortical and in vivo neurogenesis models	Purified hyperforin and hyperforin-containing preparations	TRPC6 → CREB/BDNF signaling; neurotrophic signaling; mitochondrial preservation	↑ dendritic spine formation, synaptogenesis and synaptic density; enhanced neuronal differentiation, maturation and survival	[14,54,57,91,96,120,121]
**Alzheimer’s disease**	Aβ-related cellular systems and AD animal models	Purified hyperforin and hyperforin-containing preparations	Aβ aggregation/clearance; tau-regulatory pathways; NF-κB-associated inflammation; redox and mitochondrial regulation	↓ Aβ aggregation and neurotoxicity; enhanced amyloid clearance; possible attenuation of tau hyperphosphorylation; ↓ neuroinflammatory mediators and oxidative injury	[94,96,120,122,125,126,127,128,129,130]
**Parkinson’s disease**	Cellular and toxin-induced animal models of Parkinsonian neurodegeneration	Purified hyperforin and hyperforin-containing preparations	Dopaminergic neuroprotection; TRPC6/pro-survival signaling; antioxidant and mitochondrial pathways; anti-inflammatory mechanisms	Improved dopaminergic neuronal resilience; ↓ ROS and oxidative damage; preservation of mitochondrial integrity; attenuation of inflammatory injury	[14,55,63,96,120,131,132,133,134,135,136]
**Multiple sclerosis**	Disease-relevant inflammatory/demyelinating experimental models	Hyperforin-related interventions, including purified hyperforin and/or hyperforin-containing preparations	Cytokine suppression; immune-cell regulation; anti-inflammatory, antioxidant and neuroprotective pathways	Reduced inflammatory activity and oxidative stress; modulation of immune activation; potential preservation of neuronal integrity	[138,139,140,141]
**Huntington’s disease**	No robust disease-specific efficacy program identified; evidence mainly extrapolated from mechanistic models	Mainly mechanistic evidence for purified hyperforin/hyperforin-related interventions	Autophagy; mitochondrial protection; antioxidant and anti-inflammatory pathways	Biological rationale for neuronal resilience and proteostasis, but no convincing demonstration of mutant-huntingtin clearance or disease modification	[5,14,137]
**Amyotrophic lateral sclerosis**	Disease-specific preclinical evidence very limited	Mainly mechanistic evidence for hyperforin	Mitochondrial protection; autophagy; antioxidant and anti-inflammatory pathways	Theoretical neuroprotective relevance, but no established efficacy in ALS-specific experimental models	[5,14,142]

* Note: Preclinical evidence in Section 5 includes studies using purified hyperforin as well as hyperforin-containing *H. perforatum* preparations. Findings obtained with botanical preparations cannot be attributed unequivocally to hyperforin because other bioactive constituents may contribute to the observed effects. The strength of evidence also differs substantially across disease domains; therefore, the findings summarized above should be interpreted as preclinical evidence rather than confirmation of clinical efficacy.

**Table 3:** Key preclinical evidence for the immunomodulatory and autoimmune-related effects of purified hyperforin and hyperforin-containing preparations *.

Immune Target/Disease Area	Main Experimental Context	Type of Intervention	Key Mechanisms/Targets	Most Important Preclinical Findings	References
**Macrophage regulation**	In vitro macrophage systems and in vivo inflammatory models	Purified hyperforin and hyperforin-containing preparations	NF-κB; MAPK; TNF-α; IL-1β; IL-6; nitric oxide; PGE_2_; macrophage polarization	↓ pro-inflammatory mediator production; reduced macrophage inflammatory activity; possible shift from M1-associated toward M2-associated reparative phenotype	[61,94,120,146]
**Neutrophil regulation**	In vitro and in vivo inflammatory models	Purified hyperforin and hyperforin-containing preparations	Oxidative burst; elastase; myeloperoxidase; chemotaxis; adhesion; transmigration	↓ neutrophil activation, ROS generation, mediator release and tissue recruitment; reduced elastase and myeloperoxidase activity	[63,66,148]
**Dendritic-cell regulation**	Experimental dendritic-cell models	Purified hyperforin and hyperforin-containing preparations	Co-stimulatory signaling; pro-inflammatory cytokine production; dendritic-cell maturation	↓ co-stimulatory molecule expression and inflammatory cytokine production; tendency toward a less inflammatory dendritic-cell phenotype	[94,149]
**T-cell responses**	In vitro T-cell systems and in vivo inflammatory/autoimmune models	Purified hyperforin; in vivo studies also include hyperforin-containing preparations	NF-κB; STAT; MAPK; Ca^2+^-dependent signaling; Th1/Th17 pathways	Modulation of T-cell activation, proliferation and differentiation; ↓ Th1/Th17-associated responses and IFN-γ, TNF-α and IL-17 production	[94,139,140]
**B-cell/humoral immunity**	Experimental immune-cell models	Purified hyperforin and hyperforin-containing preparations	Indirect regulation through inflammatory signaling and T-cell–B-cell interactions	Possible indirect modulation of humoral immunity; direct effects on B-cell differentiation, plasma cells and autoantibody production remain insufficiently defined	[139,140,149]
**Rheumatoid arthritis-related inflammation**	Inflammatory and immune models relevant to RA; robust RA-specific models remain limited	Hyperforin-related interventions; exact preparation varies across cited evidence	NF-κB; inflammatory cytokines; oxidative stress; immune-cell activation	Suppression of inflammatory and oxidative pathways relevant to synovitis; sustained protection against joint damage has not yet been established	[61,63,140,146,149,150]
**Inflammatory bowel disease/mucosal inflammation**	Experimental colitis and mucosal-barrier models	Hyperforin-related interventions	Inflammatory cytokine signaling; epithelial-barrier regulation; oxidative stress	Attenuation of inflammatory signaling and cytokine production; support of epithelial barrier integrity	[14,63,150,151]
**Psoriasis-like inflammation**	Psoriasis-like inflammatory and keratinocyte models	Hyperforin-related interventions	Inflammatory signaling; keratinocyte proliferation and differentiation	↓ psoriasis-like inflammation; modulation of keratinocyte growth and differentiation	[153,154,155]
**Type 1 diabetes-related β-cell injury**	Pancreatic β-cell inflammatory/oxidative injury models	Hyperforin and hyperforin-containing preparations	Cytokine-associated inflammation; oxidative stress	Protection of β cells against inflammatory and oxidative injury	[156,157]
**Other autoimmune/immune-mediated disorders**	Mainly mechanistic extrapolation rather than disease-specific models	Hyperforin and hyperforin-containing preparations	Shared anti-inflammatory, antioxidant, hepatoprotective, antifibrotic and wound-healing pathways	Potential relevance to SLE, autoimmune thyroid disease, uveitis, autoimmune hepatitis, Sjögren’s syndrome, systemic sclerosis, vitiligo and alopecia areata remains largely exploratory	[158,159,160,161]

* Note: The preclinical evidence summarized in Section 6 includes studies using purified hyperforin and hyperforin-containing *H. perforatum* preparations. Where botanical preparations were evaluated, the observed effects cannot be attributed unequivocally to hyperforin alone. Evidence is comparatively more developed for macrophage- and T-cell-related effects, whereas direct B-cell regulation, dendritic-cell tolerance, and several proposed autoimmune indications remain insufficiently characterized. For some disease-specific applications, particularly rheumatoid arthritis and several systemic autoimmune disorders, the evidence is largely mechanistic or indirect rather than derived from robust disease-specific models.

**Table 4:** Key preclinical evidence for the cardiovascular and metabolic effects of purified hyperforin and hyperforin-containing preparations *.

Cardiometabolic Domain	Main Preclinical Models/Evidence	Type of Intervention	Principal Mechanisms/Targets	Most Important Preclinical Findings	References
**Endothelial function and vascular protection**	Endothelial and vascular experimental models; limited in vivo vascular studies	Predominantly hyperforin-containing preparations; purified hyperforin to a lesser extent	Oxidative stress; inflammatory signaling; eNOS/NO bioavailability; NF-κB; adhesion molecules; mitochondrial protection	Preservation of NO-related signaling; reduced endothelial inflammatory activation and oxidative injury; potential reduction in leukocyte recruitment and vascular macromolecular oxidation	[63,120,150,160,164,165,166]
**Atherosclerosis-related inflammation and foam-cell formation**	In vitro macrophage/vascular models and limited in vivo models	Purified hyperforin and hyperforin-containing preparations	NF-κB; TNF-α; IL-1β; IL-6; macrophage inflammatory responses; foam-cell formation	↓ inflammatory mediator expression; modulation of macrophage responses and foam-cell formation; potential attenuation of early atherogenic processes	[61,63,68,120,164,166,168,169]
**Atherosclerosis-related oxidative stress**	Cellular and in vivo oxidative-stress models relevant to vascular disease	Purified hyperforin and hyperforin-containing preparations	ROS; SOD; catalase; glutathione systems; mitochondrial integrity	↓ intracellular ROS; ↑ endogenous antioxidant defenses; reduced oxidative processes relevant to LDL modification and vascular injury	[65,66,156,170]
**Cholesterol homeostasis**	In vitro and preclinical metabolic models	Purified hyperforin and hyperforin-containing preparations	Inflammatory/metabolic signaling; nuclear receptors and transcriptional regulators of lipid metabolism; LDL oxidation	Potential modulation of cholesterol synthesis, transport and cellular handling; reduced oxidative modification of LDL	[63,160,164,170,171,172,173,174,175,176]
**Triglyceride and fatty-acid metabolism**	In vitro and in vivo metabolic models	Purified hyperforin and hyperforin-containing preparations	Mitochondrial function; energy sensing; fatty-acid utilization; lipid-storage pathways	Potential ↑ fatty-acid oxidation and mitochondrial metabolic capacity; ↓ intracellular lipid accumulation	[96,160,170]
**Adipogenesis and adipose-tissue expansion**	Preadipocyte/adipocyte models and in vivo obesity-related studies	Predominantly hyperforin-containing *H. perforatum* preparations	PPARγ; C/EBPα; C/EBPβ; aP2/FABP4; adiponectin; lipoprotein lipase	↓ adipocyte differentiation and intracellular lipid accumulation; suppression of major adipogenic transcription factors and downstream markers	[179,180,181,182,183]
**Energy metabolism in obesity**	In vitro and in vivo metabolic/obesity-related models	Purified hyperforin and hyperforin-containing preparations	AMPK; mitochondrial function; oxidative phosphorylation; mitochondrial biogenesis; fatty-acid oxidation	Improved cellular energy metabolism and metabolic flexibility; potential ↑ glucose utilization and fatty-acid oxidation and ↓ lipogenesis	[66,67,84,96,170]
**Adipokine regulation**	Adipocyte/adipose-tissue in vitro and in vivo models	Purified hyperforin and hyperforin-containing preparations	Adiponectin; inflammatory mediators; metabolic regulatory pathways	↑ adiponectin and ↓ pro-inflammatory mediator expression in adipose tissue; potential improvement in insulin-sensitive metabolic signaling	[170,179,182,183]
**Glucose homeostasis and insulin resistance**	In vitro metabolic systems and animal models of metabolic dysfunction	Purified hyperforin and hyperforin-containing preparations	Glucose transport; oxidative/inflammatory signaling; mitochondrial bioenergetics; AMPK; metabolic signaling	Potential improvement in cellular glucose utilization; attenuation of metabolic inflammation and oxidative stress; support of glucose homeostasis	[84,96,170,181,182,188]
**Insulin signaling and β-cell protection**	Predominantly in vitro models with limited animal support	Purified hyperforin and hyperforin-containing preparations	TNF-α; IL-6; AMPK; mitochondrial function; antioxidant pathways	Potential improvement of insulin-related metabolic responses; protection of pancreatic β cells and insulin-responsive tissues from inflammatory/oxidative injury	[65,84,156,164,165,170,188]

* Note: The preclinical literature summarized in Section 7 includes studies using purified hyperforin as well as hyperforin-containing *H. perforatum* preparations. Where botanical preparations were used, compound-specific attribution remains uncertain because other phytochemicals may contribute to the observed effects. The most consistent preclinical evidence concerns obesity-associated metabolic dysfunction, adipose-tissue biology, insulin resistance, and glucose homeostasis, whereas evidence for atherosclerosis, endothelial dysfunction, and clinically relevant cardiovascular outcomes remains comparatively limited and often mechanistic or indirect.

**Table 5:** Key preclinical evidence for the anticancer effects of purified hyperforin and hyperforin-containing preparations *.

Cancer Domain/Model	Main Preclinical Models	Type of Intervention	Principal Mechanisms/Targets	Most Important Findings	References
**General antiproliferative activity/cell-cycle regulation**	Multiple human cancer cell lines; limited in vivo confirmation	Purified hyperforin and hyperforin-containing preparations	Cyclins D1/E/A; CDK2/4/6; p21; p27; PI3K/Akt; ERK/MAPK; STAT3; NF-κB	↓ cancer-cell proliferation; cell-cycle arrest; downregulation of cyclins/CDKs and upregulation of cell-cycle inhibitors	[70,73,192]
**Apoptosis induction**	Multiple malignant cell lines	Purified hyperforin and hyperforin-containing preparations	Mitochondrial membrane depolarization; cytochrome *c*; caspase-9/-3; Bcl-2/Bcl-xL; Bax/Bak; death-receptor pathways	Activation of intrinsic and extrinsic apoptosis; ↓ anti-apoptotic proteins; ↑ pro-apoptotic mediators	[69,73,74,75,76,194]
**Autophagy regulation**	Predominantly in vitro cancer models	Purified hyperforin and hyperforin-containing preparations	PI3K/Akt/mTOR; AMPK; autophagic signaling	Context-dependent modulation of autophagy with possible contribution to growth inhibition and treatment sensitivity	[74,75,76]
**Breast cancer**	Human breast cancer cell lines, including MCF-7 and MDA-MB-468; limited animal models	Purified hyperforin and hyperforin-containing preparations	NF-κB; PI3K/Akt; ERK/MAPK; STAT3; estrogen-responsive signaling; apoptosis; EMT-related pathways	↓ proliferation, migration and invasion; ↑ apoptosis; suppression of EMT/stemness-associated signaling; limited in vivo evidence of ↓ tumor growth, angiogenesis and metastatic dissemination	[70,73,74,75,76,192,196,197,198,199,200,201]
**Colorectal cancer**	Human colorectal cancer cell lines; limited in vivo/xenograft evidence	Purified hyperforin and hyperforin-containing preparations	JAK1/STAT3; PI3K/Akt; ERK/MAPK; NF-κB; Fas/FasL; caspases; PARP; Wnt/β-catenin	↓ proliferation, clonogenic survival, migration and invasion; ↑ intrinsic/extrinsic apoptosis; suppression of pro-survival signaling	[67,69,73,75,76,202,203]
**Prostate cancer**	LNCaP, PC-3 and DU145 prostate cancer cells	Predominantly purified hyperforin	PI3K/Akt; Bcl-2; MCL-1; XIAP; cytochrome *c*; caspase-9/-3; BAK, BAD and NOXA	↓ proliferation and DNA synthesis; ↑ mitochondrial and caspase-dependent apoptosis	[69,70,73,75,192]
**Leukemia and hematological malignancies**	AML, CLL and other leukemic cell models	Predominantly purified hyperforin; hyperforin-containing preparations to a lesser extent	Akt; Bcl-2 family; mitochondrial depolarization; cytochrome *c*; caspases; NF-κB; STAT signaling	↓ leukemic-cell proliferation; induction of mitochondrial dysfunction and apoptosis; some evidence of greater malignant-cell susceptibility than normal hematopoietic cells	[66,71,192,204,205,206]
**Tumor-microenvironment inflammatory signaling**	Tumor-related cellular and inflammatory models	Mainly purified hyperforin; hyperforin-containing preparations to a lesser extent	NF-κB; TNF-α; IL-1β; IL-6; PGE_2_; COX-2	Suppression of inflammatory signaling relevant to tumor–stromal and tumor–immune interactions; direct in vivo TME reprogramming remains unproven	[62,66,75,76,203,206,207]
**Tumor angiogenesis**	Endothelial-cell assays; capillary-tube models; tumor vascularization and lymphangiogenesis models	Predominantly purified hyperforin	VEGF-associated signaling; NF-κB-dependent endothelial responses; multiple angiogenic growth-factor pathways	↓ endothelial proliferation and migration; ↓ capillary-tube formation, tumor vascularization and lymphangiogenesis	[199,203,210,211,212]
**Tumor-associated immune modulation**	Predominantly in vitro immune/TME-related models	Purified hyperforin and hyperforin-containing preparations	TNF-α; IL-1β; IL-6; IL-17A; IFN-γ; NF-κB; STAT3; Th1/Th17 responses	Modulation of inflammatory cytokines and adaptive immune signaling; direct evidence for antitumor immune reprogramming remains limited	[66,153,205]
**Combination with chemotherapy**	In vitro cancer-cell sensitization studies	Purified hyperforin and hyperforin-containing preparations	PI3K/Akt; NF-κB; STAT3; apoptosis sensitization	Potential enhancement of selected anticancer-agent activity and increased susceptibility to apoptosis; in vivo combination evidence remains limited	[67,70,192,206]

* Note: The anticancer evidence summarized in Section 8 is overwhelmingly preclinical and is derived predominantly from in vitro studies. Purified hyperforin has been used frequently in mechanistic cancer research, although some studies have employed hyperforin-containing *H. perforatum* preparations. Findings obtained with botanical preparations cannot be attributed unequivocally to hyperforin because other phytochemicals may contribute to the observed effects. In vivo validation remains comparatively limited, and clinically achievable exposure, tumor-specific pharmacokinetics, therapeutic selectivity, and combination-related drug–herb interactions remain important unresolved issues.

**Table 6:** Key preclinical evidence for the antimicrobial and antiviral effects of purified hyperforin and hyperforin-containing preparations *.

Antimicrobial Domain	Main Experimental Models/Pathogens	Type of Intervention	Principal Mechanisms	Most Important Preclinical Findings	References
**Gram-positive antibacterial activity**	In vitro studies against *Staphylococcus aureus*, MRSA, *Streptococcus pneumoniae*, *S. pyogenes*, *Enterococcus faecalis*, *Bacillus subtilis* and related species	Purified hyperforin and hyperforin-containing *H. perforatum* preparations	Disruption of bacterial membrane integrity; collapse of transmembrane electrochemical gradients; altered proton motive force, ion homeostasis and ATP generation	Strongest antibacterial evidence; low MICs reported for several Gram-positive species, particularly resistant staphylococci; inhibition of bacterial growth and viability	[220,221,222,223,224,225,226,227,228,229,230]
**Gram-negative antibacterial activity**	In vitro studies against *Escherichia coli*, *Pseudomonas aeruginosa*, *Klebsiella pneumoniae*, *Salmonella enterica* and *Helicobacter pylori*	Purified hyperforin and hyperforin-containing preparations	Membrane perturbation and increased permeability; disruption of bacterial energetics	Antibacterial activity reported, but generally weaker than against Gram-positive species; activity may be enhanced in combination with conventional antibiotics	[220,223,226,229,231,232,233,234]
**Multidrug-resistant Gram-negative pathogens/antibiotic potentiation**	MDR *P. aeruginosa*, *K. pneumoniae* and *Acinetobacter baumannii*	Purified hyperforin + polymyxin B	Increased membrane permeability; membrane disruption; oxidative stress; biofilm interference	Potentiation of polymyxin B activity and enhanced bacterial killing, supporting a potential role as an antimicrobial adjuvant	[229]
**Antibiofilm activity**	Biofilm-forming *S. aureus*/MRSA, *E. faecalis* and other bacterial models	Purified hyperforin, hyperforin-related compounds and hyperforin-containing preparations	Inhibition of biofilm formation; disruption of established biofilms; membrane-related effects	↓ biofilm-associated growth; disruption of established biofilms; enhanced susceptibility of biofilm-associated microorganisms to antimicrobial agents	[228,229,259,260]
**Antifungal activity**	In vitro models of *Candida*, *Cryptococcus*, *Trichophyton* and *Microsporum* spp.; more limited data for *Aspergillus*	Predominantly hyperforin-containing preparations; purified hyperforin or related compounds to a lesser extent	Fungal membrane disruption; altered ion gradients; metabolic/oxidative stress; disturbance of bioenergetics	Inhibition of fungal growth and viability, particularly in *Candida* spp.; possible inhibition of fungal biofilms and virulence-associated processes	[84,237,238,239,240,241,242]
**Enveloped viruses**	In vitro studies involving HSV, HIV and influenza viruses; limited HCV and coronavirus-related evidence	Predominantly hyperforin-containing preparations; purified hyperforin to a lesser extent	Interference with intracellular viral replication/propagation; possible post-entry effects; inhibition of viral RNA synthesis; host–cell pathway modulation	Antiviral activity reported for selected enveloped viruses; however, the contribution of hyperforin differs among viruses and preparations	[244,245,246,247,248,249,250,251]
**Coronaviruses/SARS-CoV-2-related evidence**	In vitro coronavirus models and *H. perforatum* preparation studies	Mainly *H. perforatum* preparations; some purified-hyperforin evidence	Viral replication/host–cell pathway modulation	Antiviral activity has been reported, but much of the activity of botanical preparations appears attributable to hypericin and pseudohypericin rather than hyperforin	[244,245,246,247,248,249,250]
**Non-enveloped viruses**	Limited mechanistic and virus-specific in vitro studies	Hyperforin-containing preparations and, to a lesser extent, purified hyperforin	Modulation of NF-κB, MAPK, Ca^2+^-dependent and ER-stress signaling; possible interference with intracellular viral propagation	Evidence remains sparse and largely indirect; specific antiviral efficacy of hyperforin has not been established	[8,66,219,242,248,249,254,255]
**Activity against antimicrobial-resistant Gram-positive pathogens**	MRSA, *S. epidermidis*, *Streptococcus* spp. and *Enterococcus* spp.	Predominantly hyperforin-containing preparations; purified hyperforin to a lesser extent	Multitarget membrane-active antibacterial actions; biofilm disruption	Activity against resistant Gram-positive strains; available evidence suggests that hyperforin resistance may not necessarily confer cross-resistance to conventional antibiotics	[83,229,233,258]
**Host-directed anti-inflammatory effects during infection**	Experimental inflammatory/infection-associated models	Purified hyperforin and hyperforin-containing preparations	IL-6; prostaglandins; leukotrienes; NF-κB; STAT; Ca^2+^ signaling; oxidative stress	Suppression of infection-associated inflammatory mediators and oxidative injury; may reduce host tissue damage but should not be interpreted as direct antimicrobial efficacy	[66,207,219,261]

* Note: The antimicrobial evidence summarized in Section 9 is predominantly preclinical and largely based on in vitro studies. The strongest and most consistent findings concern antibacterial activity against Gram-positive organisms, whereas activity against Gram-negative bacteria is generally weaker and may be particularly relevant in combination or adjuvant strategies. Antifungal and antiviral studies more frequently employ hyperforin-containing *Hypericum perforatum* preparations rather than purified hyperforin; consequently, effects observed with botanical preparations cannot be attributed unequivocally to hyperforin. This is particularly important in antiviral studies, where hypericin and pseudohypericin may account for a substantial proportion of the activity reported for *H. perforatum* extracts.

**Table 7:** Key preclinical evidence for the gastrointestinal and gut-health effects of purified hyperforin and hyperforin-containing preparations *.

Gastrointestinal Domain/Model	Main Preclinical Models	Type of Intervention	Principal Mechanisms/Targets	Most Important Preclinical Findings	References
**Intestinal barrier integrity**	Experimental epithelial/barrier models; indirect preclinical evidence	Purified hyperforin and hyperforin-containing preparations	Suppression of TNF-α, IL-1β and IL-6; attenuation of oxidative injury; protection of junctional proteins	Potential preservation of epithelial cohesion and reduction in pathological intestinal permeability; direct restoration of tight-junction architecture by purified hyperforin remains insufficiently demonstrated	[160,219,269]
**Mucosal protection and epithelial repair**	Cellular and tissue models relevant to epithelial protection and repair	Hyperforin and hyperforin-containing preparations	TRPC6; ERK1/2; mitochondrial resilience/dynamics; Lon protease-1; anti-inflammatory and antioxidant pathways	Potential protection against cytokine-mediated and oxidative epithelial injury; support of cellular survival and mucosal repair	[207,219,261,271,272]
**Intestinal inflammatory signaling**	Experimental inflammatory and mucosal immune models	Predominantly hyperforin-related experimental interventions	NF-κB; STAT1; TNF-α; IL-1β; IL-6; iNOS; COX-2; ROS	↓ pro-inflammatory cytokines and inflammatory enzymes; attenuation of oxidative stress and immune-cell activation	[8,61,66,170,176,219,270,274]
**Experimental colitis/IBD-related inflammation**	Chemically induced rodent colitis, including TNBS-associated models	Predominantly hyperforin-containing *H. perforatum* preparations/extracts	NF-κB inhibition; cytokine modulation; antioxidant activity; epithelial-barrier protection	↓ colonic inflammation, cytokine responses, immune-cell infiltration and oxidative stress; improved histopathology, mucosal architecture, epithelial-barrier function and disease indices	[170,282,283]
**Gut microbiota and microbiota-derived metabolites**	Chronic-stress mouse models with microbiota and metabolomic assessments	Hyperforin administration	Gut microbial composition; microbiota-derived metabolites; colonic mucus-barrier regulation	Alterations in beneficial microbial taxa and fecal metabolite profiles; improved colonic mucus-barrier integrity; microbiota-associated contribution to gut–brain effects proposed	[277,278]
**IBS-related mechanisms**	No established IBS-specific hyperforin model; indirect mechanistic and animal evidence	Hyperforin	Anti-inflammatory and antioxidant signaling; leukocyte activation; ROS; intestinal barrier and microbiota-related mechanisms	Potential improvement in mucus-barrier integrity and microbiota-associated metabolic profiles, but no direct demonstration of efficacy in validated IBS models	[176,277,278,281]
**Gastric mucosal injury/gastric lesions**	Ethanol-induced gastric mucosal injury and restraint-stress gastric lesion models in rats	Hyperforin-containing *H. perforatum* extracts/preparations	Anti-inflammatory and antioxidant effects; preservation of mucosal integrity; possible H^+^/K^+^-ATPase-related effects	Reduced gastric mucosal injury and lesion severity; enhanced gastric protection/healing in experimental models	[284,285,286]
**Pancreatic and hepatobiliary injury**	Experimental inflammatory/oxidative injury models	Predominantly hyperforin-containing preparations; some hyperforin-related evidence	NF-κB/STAT signaling; oxidative stress; inflammatory cytokines; cellular integrity	Potential attenuation of inflammatory and oxidative tissue injury and preservation of parenchymal-cell function; disease-specific evidence remains limited	[62,63,66,156,160,188,284,285,286]
**Gastrointestinal antimicrobial effects**	In vitro studies against *Helicobacter pylori* and other GI-associated pathogens	Predominantly hyperforin-containing preparations	Direct antimicrobial activity; membrane-related effects; possible antibiofilm activity	Inhibition of selected gastrointestinal pathogens; possible adjunctive relevance to mucosal protection, although the independent contribution of hyperforin remains unclear	[176,224,229,233,259]

* **Note:** The gastrointestinal evidence summarized in Section 10 is predominantly preclinical and heterogeneous with respect to experimental model and intervention. Experimental colitis currently represents the most developed disease-specific application, whereas evidence for intestinal barrier restoration, IBS, microbiota-mediated effects, gastric disorders, pancreatic injury, and hepatobiliary conditions remains comparatively limited. Importantly, many gastrointestinal studies have employed hyperforin-containing *H. perforatum* preparations rather than purified hyperforin; therefore, observed effects cannot be attributed unequivocally to hyperforin alone.

**Table 8:** Key preclinical evidence for the emerging therapeutic applications of purified hyperforin and hyperforin-containing preparations *.

Application/Domain	Main Preclinical Models	Type of Intervention	Principal Mechanisms/Targets	Most Important Preclinical Findings	References
**Wound healing—keratinocyte differentiation and re-epithelialization**	Keratinocyte cultures and ex vivo skin/wound models	Purified hyperforin and hyperforin-containing preparations	TRPC6-dependent Ca^2+^ signaling; keratinocyte differentiation; epidermal-barrier regulation	↑ epidermal differentiation markers; accelerated wound closure; enhanced re-epithelialization and epidermal-barrier restoration	[154,271,288]
**Wound healing—anti-inflammatory and antimicrobial effects**	Cellular inflammatory models and wound-related experimental systems	Purified hyperforin and hyperforin-containing preparations	↓ TNF-α, IL-1β, IL-6, IL-17A and IL-23; ↓ prostaglandin synthesis and oxidative stress; antibacterial/antibiofilm activity	Reduced inflammatory responses and microbial burden potentially interfering with wound repair; activity against *S. aureus*, MRSA, *Streptococcus* spp. and selected resistant bacteria	[63,153,161,176,219,220,223,271,289]
**Wound healing—in vivo tissue repair**	Animal wound-healing models	Predominantly hyperforin-rich *H. perforatum* preparations/formulations	Anti-inflammatory, antioxidant, antimicrobial and regenerative mechanisms; angiogenesis; ECM remodeling	Accelerated wound closure; ↑ granulation tissue and angiogenesis; improved collagen deposition/organization, re-epithelialization, ECM remodeling and histological healing	[290,291,292,293]
**Psoriasis and keratinocyte hyperproliferation**	Keratinocyte models and psoriasis-related experimental systems	Predominantly purified hyperforin; some hyperforin-containing preparations	TRPC6-dependent Ca^2+^ signaling; keratin-1/-10, involucrin and transglutaminase; Th17/TNF-α-associated inflammation	↓ pathological keratinocyte proliferation and DNA synthesis; ↑ differentiation; suppression of inflammatory cytokine responses relevant to psoriasis	[153,154,271,297]
**Atopic dermatitis and inflammatory skin dysfunction**	Atopic dermatitis-related and inflammatory skin models	Purified hyperforin and hyperforin-containing preparations	Epidermal-barrier regulation; inflammatory and oxidative signaling; keratinocyte differentiation	Improved experimental epidermal-barrier function and attenuation of inflammatory and oxidative responses; disease-specific evidence remains limited	[271,274,298]
**Dermatological antimicrobial protection**	Skin-associated microbial systems, particularly *S. aureus*	Purified hyperforin and hyperforin-containing preparations	Direct antibacterial activity; membrane-active antimicrobial mechanisms	Antibacterial activity against organisms relevant to inflammatory skin disease, potentially complementing barrier-restorative and anti-inflammatory effects	[70,233,299]
**Neuropathic and inflammatory pain**	Rodent neuropathic-pain models, including chronic constriction injury, diabetic neuropathy and oxaliplatin-induced neuropathy	Predominantly hyperforin-containing *H. perforatum* extracts/preparations	Opioid-dependent mechanisms; ↓ PGE_2_ and inflammatory signaling; possible monoaminergic/GABAergic, TRPC6, redox and neuroinflammatory modulation	↓ mechanical hyperalgesia and antinociceptive responses; improved nociceptive thresholds in several neuropathic-pain models	[63,302,303,304]
**Osteoarthritis/cartilage degeneration**	Experimental osteoarthritis models	Tetrahydrohyperforin, a chemical analogue of hyperforin	Autophagy regulation; anti-inflammatory signaling; cartilage-protective mechanisms	↓ cartilage degeneration and inflammatory changes; ↑ protective autophagy	[306]
**Bone remodeling and skeletal homeostasis**	Osteoblast/osteoclast-related experimental systems	Hyperforin-related interventions; compound-specific purified-hyperforin evidence remains limited	↓ PGE_2_ and inflammatory cytokines; ↓ oxidative stress; osteoblast differentiation/mineralization; osteoclast regulation	Potential ↑ osteoblast differentiation and mineralization and ↓ osteoclast-mediated bone degradation; possible preservation of bone mass	[62,63,307]
**Healthy aging/cellular resilience**	Predominantly mechanistic cellular and experimental evidence rather than dedicated lifespan models	Hyperforin-related experimental interventions; no well-developed aging-specific intervention program	Antioxidant defense; inflammation/inflammaging; mitochondrial homeostasis; autophagy/proteostasis; TRPC6/BDNF-associated neuroprotection	Potential attenuation of oxidative and inflammatory damage, support of mitochondrial function and autophagic quality control, and improved cellular resilience; direct lifespan extension has not been established	[5,6,8,309]

* Note: The preclinical evidence summarized in Section 11 differs substantially in maturity and intervention specificity. Wound healing and dermatological applications are supported by comparatively broader experimental evidence, including purified hyperforin studies and tissue-specific models. In contrast, pain studies rely largely on hyperforin-containing *H. perforatum* preparations, while musculoskeletal evidence includes studies of tetrahydrohyperforin and other hyperforin-related interventions. Evidence concerning healthy aging remains predominantly mechanistic and indirect, with limited aging-specific or lifespan studies. Accordingly, findings obtained with botanical preparations or hyperforin analogues should not be interpreted as equivalent to compound-specific efficacy of purified hyperforin.

**Table 9:** Summary of available clinical evidence on hyperforin-containing preparations or formulations across disease domains.

Clinical Indication	Representative Clinical Evidence	Population	Intervention and Approximate Duration	Main Clinical Findings	References
**Major depressive disorder, mild-to-moderate**	RCTs, comparative trials, systematic reviews, and meta-analyses	Adults with mild-to-moderate major depressive disorder	Standardized hyperforin-containing preparations, typically administered for approximately 4–12 weeks	Significant reductions in HAM-D, MADRS, BDI, and CGI scores; efficacy superior to placebo and broadly comparable to SSRIs and TCAs	[104,106,108,109,118,310,311,312,313,314,315,316,317,318,319,320,321]
**Stress-associated depressive symptoms and adjustment disorders**	RCTs and observational studies	Adults with subclinical, adjustment-related, or stress-associated depressive symptoms	Hyperforin-containing preparations, generally administered for approximately 4–12 weeks	Improvements in depressive symptoms, emotional well-being, stress coping, and psychological functioning	[311,312,313,314,315,316]
**Dysthymia and persistent depressive symptoms**	Clinical trials and observational studies	Adults with chronic low-grade or persistent depressive symptoms	Standardized hyperforin-containing preparations, generally administered for approximately 6–12 weeks	Improvements in mood symptoms and psychological functioning	[310,311,312,313,314,315,316]
**Generalized anxiety symptoms and mixed anxiety–depressive states**	Small RCTs and observational studies	Adults with anxiety symptoms, mixed anxiety–depression, or stress-related psychopathology	Hyperforin-containing preparations, typically administered for approximately 4–12 weeks	Reductions in anxiety severity and associated stress-related symptoms	[311,322,323,324,325]
**Stress-related psychological disorders**	Clinical and observational studies	Individuals experiencing chronic psychosocial stress or adjustment-related symptoms	Standardized hyperforin-containing preparations, generally administered for approximately 4–12 weeks	Improvements in stress tolerance, emotional stability, psychological well-being, and quality of life	[311,322,323,324,325]
**Cognitive dysfunction and age-related cognitive decline**	Pilot studies and small clinical investigations	Older adults and individuals with depressive symptoms, chronic stress, or age-related cognitive complaints	Hyperforin-containing preparations, evaluated over approximately 4–12 weeks in most short-term investigations	Possible improvements in attention, memory, executive function, and overall cognitive efficiency	[326,327,328,329]
**Dementia and neuropsychiatric symptoms associated with cognitive impairment**	Small open-label and exploratory studies	Patients with dementia presenting agitation, apathy, depression, or other behavioral symptoms	Hyperforin-containing preparations, evaluated over an estimated 4–12-week treatment period	Improvements reported in selected behavioral and psychological symptoms of dementia	[329,336]
**Inflammatory skin disorders, including atopic dermatitis and psoriasis**	Small RCTs, pilot trials, and dermatological studies	Patients with atopic dermatitis, psoriasis, or related inflammatory skin conditions	Topical hyperforin-rich formulations, generally applied for approximately 2–8 weeks	Reductions in erythema, pruritus, inflammation, and lesion severity; possible improvement in skin-barrier function	[271,299,332,333,334,335]
**Wound healing and tissue repair**	Small clinical studies and topical intervention trials	Patients with acute or chronic wounds	Topical hyperforin-rich formulations, generally evaluated for approximately 2–12 weeks, depending on wound type and healing time	Enhanced epithelial regeneration, tissue repair, and wound closure reported in selected studies	[271,299,332,333,334,335]

**Table 10:** Source-specific evidence for the safety, toxicological profile, and clinical significance of hyperforin and hyperforin-containing preparations *.

Category	Evidence Source & Preparations Evaluated	Source-Specific Findings	Clinical Significance	References
**Overall safety and tolerability**	RCTs, comparative trials, systematic reviews, and post-marketing studies of standardized hyperforin-containing preparations	Good tolerability with low discontinuation and serious adverse-event rates; fewer sexual, sedative, cognitive, metabolic, and anticholinergic adverse effects than several antidepressants.	Supports short- to medium-term use in appropriately selected patients.	[1,101,103,107,317,319,322,339,346,353,354,355,356,357,358,359,360,361]
**Acute toxicity**	Experimental animal studies of purified hyperforin and hyperforin-containing preparations	High LD_50_ values and minimal organ toxicity; adverse effects mainly at supratherapeutic doses.	Suggests a broad acute safety margin; human extrapolation remains limited.	[1,2,136,346,355,364,365,366,367,368]
**Chronic toxicity**	Repeated-dose animal studies of purified hyperforin and long-term studies of standardized hyperforin-containing preparations	Minimal cumulative organ toxicity reported; purified hyperforin and long-term data remain limited.	Long-term safety requires further investigation, particularly in polypharmacy.	[43,174,317,339,352,360,361,363,364,366,369,370,371,372,373,374,375,376,377]
**Hepatotoxicity**	Systematic reviews, RCTs, pharmacovigilance studies, and case reports of hyperforin-containing preparations	Sporadic reports without a consistent hepatotoxic signal or causal association.	Clinically significant liver injury appears uncommon; monitoring may be warranted in susceptible patients.	[1,43,361,389,390]
**Common adverse effects**	Clinical trials and post-marketing studies of standardized hyperforin-containing preparations	Mild gastrointestinal symptoms, headache, dizziness, fatigue, xerostomia, agitation, restlessness, and sleep disturbance.	Adverse effects are generally mild, transient, and rarely treatment-limiting.	[1,101,108,355,360,361]
**Neurological and psychiatric safety**	Clinical studies, case reports, and pharmacovigilance analyses of hyperforin-containing preparations	Mild agitation and sleep disturbance; rare mania or hypomania in susceptible individuals.	Monitor patients with bipolar disorder or mood instability.	[1,101,108,355,360,361,384,385,386,387,388]
**Serotonergic toxicity**	Case reports, pharmacovigilance studies, and systematic reviews of hyperforin-containing preparations used with serotonergic drugs	Occasional serotonin syndrome with concomitant serotonergic medications.	Avoid concomitant use with serotonergic medications when possible.	[346,349,382,383,394]
**Photosensitivity**	Clinical reports and safety studies of hyperforin-containing preparations	Uncommon at therapeutic doses; primarily attributed to hypericin.	Advise photoprotection in highly photosensitive individuals.	[378,379,380,381]
**Pregnancy and lactation**	Limited clinical studies and regulatory assessments of hyperforin-containing preparations	Maternal and fetal safety remains insufficiently characterized.	Generally not recommended because of insufficient safety data.	[346,411,412,413,414,415,416,417,418,419,420,421,422]
**Genotoxicity and carcinogenicity**	Experimental toxicological studies of purified hyperforin and hyperforin-containing *H. perforatum* preparations	No consistent genotoxic or carcinogenic signal identified; evidence remains limited.	Current evidence is reassuring but remains insufficient for definitive conclusions.	[66,67,70,200]
**PXR activation and CYP3A4 induction**	CYP3A4 induction Mechanistic, pharmacokinetic, and interaction studies of purified hyperforin and hyperforin-containing preparations	Hyperforin activates PXR and induces CYP3A4 in a dose-dependent manner.	May reduce efficacy of concomitant CYP3A4 substrates.	[43,173,174,340,346,347,348,352,391]
**P-glycoprotein induction**	Mechanistic and clinical pharmacokinetic studies of purified hyperforin and hyperforin-containing preparations	Increased P-glycoprotein expression reduces systemic drug exposure.	May decrease systemic exposure to P-glycoprotein substrates.	[173,174,382,392,393]
**Clinically relevant drug interactions**	Clinical interaction studies, pharmacovigilance analyses, and regulatory assessments of hyperforin-containing preparations, with mechanistic evidence from purified hyperforin	Documented interactions with antidepressants, oral contraceptives, anticoagulants, immunosuppressants, antiretrovirals, anticancer agents, and cardiovascular drugs.	Major safety concern; medication review is essential before use.	[43,173,174,340,346,347,348,349,350,351,352,382,383,391,392,393,394,395,396,397,398,399,400,401,402,403,404,405,406,407,408,409,410,418,419,420,421,422]

* Clinical findings derived from multicomponent *H. perforatum* preparations should be interpreted as preparation-specific evidence and do not establish the independent efficacy, safety, or interaction profile of purified hyperforin unless supported by compound-specific evidence.

**Table 11:** Qualitative appraisal of the relative strength and translational maturity of evidence across the principal disease areas reviewed and corresponding research priorities ^1,2^.

Therapeutic Area	Mechanistic Evidence	Animal Evidence	Human Evidence	Overall Evidence Maturity	Principal Limitation	Research Priority
**Depression and mood disorders**	★★★★★	★★★★★	★★★☆☆	Moderate–strong	Human efficacy is supported primarily by standardized hyperforin-containing *H. perforatum* preparations rather than purified hyperforin; compound-specific efficacy remains unresolved	RCTs of chemically defined/purified hyperforin; human PK, dose-finding, and target-engagement studies
**Anxiety and stress-related disorders**	★★★★☆	★★★★☆	★★☆☆☆	Moderate	Human studies are fewer and frequently involve mixed anxiety–depressive populations and complete botanical preparations	Dedicated anxiety RCTs using chemically characterized interventions and validated anxiety-specific outcomes
**Cognitive dysfunction and neurodegenerative diseases (AD and related cognitive decline)**	★★★★★	★★★★☆	★☆☆☆☆	Emerging/preliminary	Extensive mechanistic and experimental evidence but very limited disease-specific human evidence; available clinical observations largely concern botanical preparations or neuropsychiatric symptoms rather than disease modification	Translational PK/PD studies followed by early-phase disease-specific clinical trials
**Parkinson’s disease and other neurodegenerative disorders**	★★★★☆	★★★☆☆	☆☆☆☆☆	Preliminary	Evidence remains predominantly mechanistic and preclinical, with insufficient compound-specific human validation	Replication in disease-relevant animal models, exposure–response studies, and subsequent early-phase clinical evaluation
**Inflammatory and immune-mediated disorders**	★★★★☆	★★★☆☆	★☆☆☆☆	Preliminary–emerging	Strong anti-inflammatory/immunomodulatory rationale, but evidence varies substantially among diseases and many proposed indications are based on mechanistic extrapolation	Disease-specific in vivo validation using purified hyperforin followed by biomarker-guided translational studies
**Cardiometabolic disorders, including obesity and T2DM**	★★★★☆	★★★★☆	★☆☆☆☆	Emerging	Consistent metabolic and inflammatory mechanisms with supportive preclinical findings, but limited direct clinical evidence for hyperforin	Human PK/PD studies and proof-of-concept trials with metabolic and inflammatory endpoints
**Cancer**	★★★★★	★★★☆☆	☆☆☆☆☆	Preliminary	Extensive anticancer mechanisms and in vitro findings substantially exceed in vivo and human evidence; clinically achievable exposure remains uncertain	Pharmacologically realistic in vivo validation, tumor-specific PK/PD assessment, and translational oncology studies
**Dermatological disorders and wound healing**	★★★☆☆	★★★☆☆	★★☆☆☆	Moderate	Encouraging topical human evidence exists, but studies are relatively small, and formulations are heterogeneous	Larger controlled trials using standardized topical formulations and clinically relevant endpoints
**Gastrointestinal disorders**	★★★★☆	★★★☆☆	☆☆☆☆☆	Preliminary	Mechanistic and animal evidence is promising, but disease-specific clinical validation is essentially absent	Additional disease-specific in vivo studies followed by early-phase clinical investigation
**Infectious diseases**	★★★★☆	★★☆☆☆	☆☆☆☆☆	Preliminary	Antimicrobial/antiviral activity is supported mainly by in vitro evidence, with limited in vivo confirmation and uncertain clinically achievable exposure	Pharmacologically realistic in vivo validation, PK/PD characterization, and resistance/safety assessment
**Musculoskeletal disorders and pain**	★★★☆☆	★★☆☆☆	☆☆☆☆☆	Preliminary	Biological plausibility derives largely from anti-inflammatory, antioxidant, and tissue-protective mechanisms, with limited disease-specific validation	Disease-specific animal studies using defined hyperforin preparations before clinical investigation
**Healthy aging and age-related functional decline**	★★★☆☆	★★☆☆☆	☆☆☆☆☆	Exploratory	Evidence is largely indirect and extrapolated from neuroprotective, metabolic, antioxidant, and anti-inflammatory actions	Mechanistic validation in aging-specific models and identification of clinically relevant aging endpoints

^1^ Evidence ratings represent a qualitative narrative appraisal by the authors and are not derived from a validated certainty-of-evidence framework. Ratings were based on the relative quantity, consistency, disease specificity, intervention specificity, and translational maturity of mechanistic, animal, and human evidence identified in this review. ^2^ The star-based evidence ratings represent an author-derived qualitative synthesis rather than a validated evidence-grading system. Ratings were based on the relative quantity, consistency, methodological strength, disease specificity, intervention specificity, and translational maturity of mechanistic, animal, and human evidence identified in this narrative review. The ratings represent a qualitative comparative appraisal developed for the purposes of this narrative review and should not be interpreted as formal GRADE assessments, systematic certainty-of-evidence ratings, or quantitative scores. In addition, ratings represent an author-derived qualitative consensus assessment based on the predefined criteria described in the text and should not be interpreted as formal certainty-of-evidence grading. ★☆☆☆☆: very limited evidence; predominantly mechanistic or exploratory; ★★☆☆☆: limited evidence; some reproducible preclinical support but substantial gaps; ★★★☆☆: moderate evidence; consistent mechanistic/preclinical findings with emerging human support; ★★★★☆: substantial evidence; strong preclinical evidence with supportive but incomplete clinical validation; ★★★★★: extensive evidence within the relevant evidence domain, based on multiple consistent and methodologically informative studies; this rating does not necessarily indicate established clinical efficacy of purified hyperforin.

## Data Availability

No new data were created or analyzed in this study. Data sharing is not applicable to this article.

## References

[1] Nobakht S.Z., Akaberi M., Mohammadpour A.H., Tafazoli Moghadam A., Emami S.A. (2022). Hypericum perforatum: Traditional Uses, Clinical Trials, and Drug Interactions. Iran. J. Basic Med. Sci..

[2] Otero M.C., Ceric F., Miranda-Rojas S., Carreño C., Escares R., Escobar M.J., Saracini C., Atala C., Ramírez-Barrantes R., Gordillo-Fuenzalida F. (2024). Documentary Analysis of *Hypericum perforatum* (St. John’s Wort) and Its Effect on Depressive Disorders. Pharmaceuticals.

[3] Kilibarda S., Jović M.D., Milinčić D.D., Vuković S., Trifković J.Đ., Pešić M.B., Kostić A.Ž. (2025). Phytochemical Profile and Biological Activities of Rtanj’s *Hypericum perforatum* Infusion Tea and Methanolic Extracts: Insights from LC-MS/MS and HPTLC–Bioautography. Plants.

[4] Güzel M.A., Kolaç T., Menevşe İ.N., Dündar M., Zengin R., Güzel A., Uğur Y. (2026). Integrated Chemical and Biological Characterization of *Hypericum perforatum* Extract Using LC-MS/MS and In Vitro Functional Assays. Sci. Rep..

[5] Suryawanshi M.V., Gujarathi P.P., Mulla T., Bagban I. (2024). *Hypericum perforatum*: A Comprehensive Review on Pharmacognosy, Preclinical Studies, Putative Molecular Mechanism, and Clinical Studies in Neurodegenerative Diseases. Naunyn-Schmiedeberg’s Arch. Pharmacol..

[6] Poulaki S., Vlachonasios K. (2024). Secondary Metabolites of Medicinal Use in *Hypericum* spp.: A Rich History and a Promising Future. Med. Plant Biol..

[7] Wang X., Liu W., Chen S., Gao Y., Tian J., Gao J. (2024). Four New Polyprenylated Acylphloroglucinols from *Hypericum perforatum* L.. Molecules.

[8] Li X.-X., Yan Y., Zhang J., Ding K., Xia C.-Y., Pan X.-G., Shi Y.-J., Xu J.-K., He J., Zhang W.-K. (2023). Hyperforin: A Natural Lead Compound with Multiple Pharmacological Activities. Phytochemistry.

[9] Liu S., Yu B., Dai J., Chen R. (2022). Targeting the Biological Activity and Biosynthesis of Hyperforin: A Mini-Review. Chin. J. Nat. Med..

[10] Wurglics M., Schubert-Zsilavecz M. (2006). *Hypericum perforatum*: A “Modern” Herbal Antidepressant: Pharmacokinetics of Active Ingredients. Clin. Pharmacokinet..

[11] Traeger A., Voelker S., Shkodra-Pula B., Kretzer C., Schubert S., Gottschaldt M., Schubert U.S., Werz O. (2020). Improved Bioactivity of the Natural Product 5-Lipoxygenase Inhibitor Hyperforin by Encapsulation into Polymeric Nanoparticles. Mol. Pharm..

[12] Balkrishna A., Sharma N., Srivastava D., Kukreti A., Srivastava S., Arya V. (2024). Exploring the Safety, Efficacy, and Bioactivity of Herbal Medicines: Bridging Traditional Wisdom and Modern Science in Healthcare. Future Integr. Med..

[13] El-Saadony M.T., Saad A.M., Mohammed D.M., Korma S.A., Alshahrani M.Y., Ahmed A.E., Ibrahim E.H., Salem H.M., Alkafaas S.S., Saif A.M. (2025). Medicinal Plants: Bioactive Compounds, Biological Activities, Combating Multidrug-Resistant Microorganisms, and Human Health Benefits—A Comprehensive Review. Front. Immunol..

[14] Bouron A. (2024). Cellular Neurobiology of Hyperforin. Phytother. Res..

[15] Green B.N., Johnson C.D., Adams A. (2006). Writing Narrative Literature Reviews for Peer-Reviewed Journals: Secrets of the Trade. J. Chiropr. Med..

[16] Ferrari R. (2015). Writing Narrative Style Literature Reviews. Med. Writ..

[17] Baethge C., Goldbeck-Wood S., Mertens S. (2019). SANRA—A Scale for the Quality Assessment of Narrative Review Articles. Res. Integr. Peer Rev..

[18] Ioannidis J.P. (2005). Why Most Published Research Findings are False. PLoS Med..

[19] Song F., Parekh S., Hooper L., Loke Y.K., Ryder J., Sutton A.J., Hing C., Kwok C.S., Pang C., Harvey I. (2010). Dissemination and Publication of Research Findings: An Updated Review of Related Biases. Health Technol. Assess..

[20] Bruňáková K., Bálintová M., Henzelyová J., Kolarčik V., Kimáková A., Petijová L., Čellárová E. (2021). Phytochemical Profiling of Several *Hypericum* Species Identified Using Genetic Markers. Phytochemistry.

[21] Çirak C., Radušienė J., Janulis V., Ivanauskas L. (2007). Secondary Metabolites in Hypericum perfoliatum: Variation Among Plant Parts and Phenological Stages. Bot. Helv..

[22] Bridi H., de Carvalho Meirelles G., von Poser G.L. (2018). Structural Diversity and Biological Activities of Phloroglucinol Derivatives from Hypericum Species. Phytochemistry.

[23] Soelberg J., Jørgensen L.B., Jäger A.K. (2007). Hyperforin Accumulates in the Translucent Glands of *Hypericum perforatum*. Ann. Bot..

[24] Kuo C.-H., Chou Y.-C., Liao K.-C., Shieh C.-J., Deng T.-S. (2020). Optimization of Light Intensity, Temperature, and Nutrients to Enhance the Bioactive Content of Hyperforin and Rutin in St. John’s Wort. Molecules.

[25] Adam P., Arigoni D., Bacher A., Eisenreich W. (2002). Biosynthesis of Hyperforin in *Hypericum perforatum*. J. Med. Chem..

[26] Wu S., Malaco Morotti A.L., Wang S., Wang Y., Xu X., Chen J., Wang G., Tatsis E.C. (2022). Convergent Gene Clusters Underpin Hyperforin Biosynthesis in St John’s Wort. New Phytol..

[27] Wu S., Morotti A.L.M., Yang J., Wang E., Tatsis E.C. (2024). Single-Cell RNA Sequencing Facilitates the Elucidation of the Complete Biosynthesis of the Antidepressant Hyperforin in St. John’s Wort. Mol. Plant.

[28] Boubakir Z., Beuerle T., Liu B., Beerhues L. (2005). The First Prenylation Step in Hyperforin Biosynthesis. Phytochemistry.

[29] Wu S., Tatsis E.C. (2024). Specialized Metabolism in St. John’s Wort. Curr. Opin. Plant Biol..

[30] Devi A.M., Devi K.K., Devi P.P., Devi M.L., Das S. (2023). Metabolic Engineering of Plant Secondary Metabolites: Prospects and its Technological Challenges. Front. Plant Sci..

[31] Orth H.C., Rentel C., Schmidt P.C. (1999). Isolation, Purity Analysis and Stability of Hyperforin as a Standard Material from *Hypericum perforatum* L.. J. Pharm. Pharmacol..

[32] Cossuta D., Vatai T., Báthori M., Hohmann J., Keve T., Simándi B. (2012). Extraction of Hyperforin and Hypericin from St. John’s Wort (*Hypericum perforatum* L.) with Different Solvents. J. Food Process Eng..

[33] Ion V., Ielciu I., Cârje A.-G., Muntean D.L., Crişan G., Păltinean R. (2022). *Hypericum* spp.—An Overview of the Extraction Methods and Analysis Techniques. Separations.

[34] Khaw K.-Y., Parat M.-O., Shaw P.N., Falconer J.R. (2017). Solvent Supercritical Fluid Technologies to Extract Bioactive Compounds from Natural Sources: A Review. Molecules.

[35] Römpp H., Seger C., Kaiser C.S., Haslinger E., Schmidt P.C. (2004). Enrichment of Hyperforin from St. John’s Wort (*Hypericum perforatum*) by Pilot-Scale Supercritical Carbon Dioxide Extraction. Eur. J. Pharm. Sci..

[36] Seger C., Römpp H., Sturm S., Haslinger E., Schmidt P.C., Hadacek F. (2004). Characterization of Supercritical Fluid Extracts of St. John’s Wort (*Hypericum perforatum* L.) by HPLC-MS and GC-MS. Eur. J. Pharm. Sci..

[37] Laina K.T., Drosou C., Stergiopoulos C., Eleni P.M., Krokida M. (2024). Optimization of Combined Ultrasound and Microwave-Assisted Extraction for Enhanced Bioactive Compounds Recovery from Four Medicinal Plants: Oregano, Rosemary, Hypericum, and Chamomile. Molecules.

[38] Agrosi M., Mischiatti S., Harrasser P.C., Savio D. (2000). Oral Bioavailability of Active Principles from Herbal Products in Humans: A Study on *Hypericum perforatum* Extracts Using the Soft Gelatin Capsule Technology. Phytomedicine.

[39] Jürgenliemk G., Nahrstedt A. (2003). Dissolution, Solubility and Cooperativity of Phenolic Compounds from *Hypericum perforatum* L. in Aqueous Systems. Pharmazie.

[40] Schulz H.U., Schürer M., Bässler D., Weiser D. (2005). Investigation of the Bioavailability of Hypericin, Pseudohypericin, Hyperforin and the Flavonoids Quercetin and Isorhamnetin Following Single and Multiple Oral Dosing of a Hypericum Extract Containing Tablet. Arzneimittelforschung.

[41] Caccia S., Gobbi M. (2009). St. John’s Wort Components and the Brain: Uptake, Concentrations Reached and the Mechanisms Underlying Pharmacological Effects. Curr. Drug Metab..

[42] El Biali M., Wölfl-Duchek M., Jackwerth M., Mairinger S., Weber M., Bamminger K., Poschner S., Rausch I., Schindler N., Lozano I.H. (2024). St. John’s Wort Extract with a High Hyperforin Content Does Not Induce P-Glycoprotein Activity at the Human Blood–Brain Barrier. Clin. Transl. Sci..

[43] Nicolussi S., Drewe J., Butterweck V., Meyer zu Schwabedissen H.E. (2020). Clinical Relevance of St. John’s Wort Drug Interactions Revisited. Br. J. Pharmacol..

[44] Sabarathinam S., Ganamurali N. (2026). Hyperforin as a Non-Steroidal Modulator of the PXR–CYP3A4 Regulatory Axis: Mechanistic and Computational Insights into Xenobiotic Metabolism. J. Steroid Biochem. Mol. Biol..

[45] Ang C.Y.W., Hu L., Heinze T.M., Cui Y., Freeman J.P., Kozak K., Luo W., Liu F.F., Mattia A., DiNovi M. (2004). Instability of *St. John’s Wort* (*Hypericum perforatum* L.) and Degradation of Hyperforin in Aqueous Solutions and Functional Beverage. J. Agric. Food Chem..

[46] Kurt A.A., Ibrahim B., Çınar H., Atsü A.N., Bursalıoğlu E.O., Bayır İ., Özmen Ö., Aslan İ. (2025). Nanoemulsion Hydrogel Delivery System of *Hypericum perforatum* L.: In Silico Design, In Vitro Antimicrobial–Toxicological Profiling, and In Vivo Wound-Healing Evaluation. Gels.

[47] Kurt A.A., Aslan İ. (2025). A Novel Liposomal In-Situ Hydrogel Formulation of *Hypericum perforatum* L.: In Vitro Characterization and In Vivo Wound Healing Studies. Gels.

[48] Talebi M., Shahbazi K., Dakkali M.S., Akbari M., Ghale R.A., Hashemi S., Sashourpour M., Mojab F., Aminzadeh S. (2025). Phytosomes: A Promising Nanocarrier System for Enhanced Bioavailability and Therapeutic Efficacy of Herbal Products. Phytomed. Plus.

[49] Zuccari G., Alfei S. (2023). Development of Phytochemical Delivery Systems by Nano-Suspension and Nano-Emulsion Techniques. Int. J. Mol. Sci..

[50] Gawai K., Rathod H., Deshmukh S. (2025). Preparation of Nanoemulsion to Enhance Delivery of Hydrophobic Drugs. Int. J. Pharm. Sci..

[51] Merk V.M., Boonen G., Butterweck V. (2025). St. John’s Wort for Depression: From Neurotransmitters to Membrane Plasticity. Int. J. Mol. Sci..

[52] Sivamaruthi B.S., Kesika P., Chaiyasut C., Ragu Varman D. (2026). Modulation of Monoaminergic Neurotransmission in Substance Use Disorders: A Neuropharmacological Perspective Focusing on Plant-Derived Metabolites. Front. Pharmacol..

[53] Singer A., Wonnemann M., Müller W.E. (1999). Hyperforin, a Major Antidepressant Constituent of, Inhibits Serotonin Uptake by Elevating Free Intracellular Na^+^. J. Pharmacol. Exp. Ther..

[54] El Hamdaoui Y., Zheng F., Fritz N., Ye L., Tran M.A., Schwickert K., Schirmeister T., Braeuning A., Lichtenstein D., Hellmich U.A. (2022). Analysis of Hyperforin (St. John’s Wort) Action at TRPC6 Channel Leads to the Development of a New Class of Antidepressant Drugs. Mol. Psychiatry.

[55] Leuner K., Kazanski V., Müller M., Essin K., Henke B., Gollasch M., Harteneck C., Müller W.E. (2007). Hyperforin—A Key Constituent of St. John’s Wort Specifically Activates TRPC6 Channels. FASEB J..

[56] Sarkar A., Srinivasan K., Datusalia A.K., Sharma S.S. (2026). Transient Receptor Potential Channels in Modulating Synaptic Plasticity and Cognitive Function: Potential Role of Pharmacological Agents. Basic Clin. Pharmacol. Toxicol..

[57] Zernov N., Popugaeva E. (2023). Role of Neuronal TRPC6 Channels in Synapse Development, Memory Formation and Animal Behavior. Int. J. Mol. Sci..

[58] Wang C.S., Kavalali E.T., Monteggia L.M. (2022). BDNF Signaling in Context: From Synaptic Regulation to Psychiatric Disorders. Cell.

[59] Tai Y., Feng S., Ge R., Du W., Zhang X., He Z., Wang Y. (2008). TRPC6 Channels Promote Dendritic Growth via the CaMKIV–CREB Pathway. J. Cell Sci..

[60] Cox D.J., Maldonado-Puebla R., Carr B. (2025). Molecular Mechanisms of Hypericin and Hyperforin in Modulating Mammalian Neurotransmitter Systems: A Review. Eur. Psychiatry.

[61] Novelli M., Menegazzi M., Beffy P., Porozov S., Gregorelli A., Giacopelli D., De Tata V., Masiello P. (2016). St. John’s Wort Extract and Hyperforin Inhibit Multiple Phosphorylation Steps of Cytokine Signaling and Prevent Inflammatory and Apoptotic Gene Induction in Pancreatic β Cells. Int. J. Biochem. Cell Biol..

[62] Hammer K.D., Hillwig M.L., Solco A.K., Dixon P.M., Delate K., Murphy P.A., Wurtele E.S., Birt D.F. (2007). Inhibition of Prostaglandin E_2_ Production by Anti-Inflammatory *Hypericum perforatum* Extracts and Constituents in RAW264.7 Mouse Macrophage Cells. J. Agric. Food Chem..

[63] Koeberle A., Rossi A., Bauer J., Dehm F., Verotta L., Northoff H., Sautebin L., Werz O. (2011). Hyperforin, an Anti-Inflammatory Constituent from St. John’s Wort, Inhibits Microsomal Prostaglandin E_2_ Synthase-1 and Suppresses Prostaglandin E_2_ Formation In Vivo. Front. Pharmacol..

[64] Oliveira A.I., Pinho C., Sarmento B., Dias A.C. (2016). Neuroprotective Activity of *Hypericum perforatum* and Its Major Components. Front. Plant Sci..

[65] Sánchez-Reus M.I., Gómez del Rio M.A., Iglesias I., Elorza M., Slowing K., Benedí J. (2007). Standardized *Hypericum perforatum* Reduces Oxidative Stress and Increases Gene Expression of Antioxidant Enzymes in Rotenone-Exposed Rats. Neuropharmacology.

[66] Menegazzi M., Masiello P., Novelli M. (2021). Anti-Tumor Activity of *Hypericum perforatum* L. and Hyperforin through Modulation of Inflammatory Signaling, ROS Generation and Proton Dynamics. Antioxidants.

[67] Cardile A., Zanrè V., Campagnari R., Asson F., Addo S.S., Orlandi E., Menegazzi M. (2023). Hyperforin Elicits Cytostatic/Cytotoxic Activity in Human Melanoma Cell Lines, Inhibiting Pro-Survival NF-κB, STAT3, AP1 Transcription Factors and the Expression of Functional Proteins Involved in Mitochondrial and Cytosolic Metabolism. Int. J. Mol. Sci..

[68] Pazarcı P., Kaplan H.M. (2025). In Vitro Apoptotic and Antiproliferative Activity of *Hypericum perforatum* Extract on Human Osteosarcoma Cell Line. J. Med. Food.

[69] Chiang I.-T., Chen W.-T., Tseng C.-W., Chen Y.-C., Kuo Y.-C., Chen B.-J., Weng M.-C., Lin H.-J., Wang W.-S. (2017). Hyperforin Inhibits Cell Growth by Inducing Intrinsic and Extrinsic Apoptotic Pathways in Hepatocellular Carcinoma Cells. Anticancer Res..

[70] Hostanska K., Reichling J., Bommer S., Weber M., Saller R. (2003). Hyperforin, a Constituent of St. John’s Wort (*Hypericum perforatum* L.) Extract, Induces Apoptosis by Triggering Activation of Caspases and, with Hypericin, Synergistically Exerts Cytotoxicity towards Human Malignant Cell Lines. Eur. J. Pharm. Biopharm..

[71] Zaher M., Tang R., Bombarda I., Merhi F., Bauvois B., Billard C. (2012). Hyperforin Induces Apoptosis of Chronic Lymphocytic Leukemia Cells through Upregulation of the BH3-Only Protein Noxa. Int. J. Oncol..

[72] You M.-K., Kim H.-J., Kook J.H., Kim H.-A. (2018). St. John’s Wort Regulates Proliferation and Apoptosis in MCF-7 Human Breast Cancer Cells by Inhibiting AMPK/mTOR and Activating the Mitochondrial Pathway. Int. J. Mol. Sci..

[73] Schempp C.M., Kirkin V., Simon-Haarhaus B., Kersten A., Kiss J., Termeer C.C., Gilb B., Kaufmann T., Borner C., Sleeman J.P. (2002). Inhibition of Tumour Cell Growth by Hyperforin, a Novel Anticancer Drug from St. John’s Wort That Acts by Induction of Apoptosis. Oncogene.

[74] Merhi F., Tang R., Piedfer M., Mathieu J., Bombarda I., Zaher M., Kolb J.-P., Billard C., Bauvois B. (2011). Hyperforin Inhibits Akt1 Kinase Activity and Promotes Caspase-Mediated Apoptosis Involving Bad and Noxa Activation in Human Myeloid Tumor Cells. PLoS ONE.

[75] Liu Y.-C., Lin K.-H., Hsieh J.-H., Chung J.-G., Tan Z.-L., Hsu F.-T., Chiang C.-H. (2019). Hyperforin Induces Apoptosis Through Extrinsic/Intrinsic Pathways and Inhibits NF-κB-Modulated Survival and Invasion Potential in Bladder Cancer. Vivo.

[76] Hsu L.-C., Kuo C.-Y., Hsu F.-T., Chang H.-F., Ou J.-J. (2023). Hyperforin Suppresses Oncogenic Kinases and Induces Apoptosis in Colorectal Cancer Cells. Vivo.

[77] You M., Lee Y.-H., Kim H.-J., Kook J.H., Kim H.-A. (2020). St. John’s Wort Suppresses Growth in Triple-Negative Breast Cancer Cell Line MDA-MB-231 by Inducing Prodeath Autophagy and Apoptosis. Nutrients.

[78] Nixon R.A., Rubinsztein D.C. (2024). Mechanisms of Autophagy–Lysosome Dysfunction in Neurodegenerative Diseases. Nat. Rev. Mol. Cell Biol..

[79] Palmer J.E., Wilson N., Son S.M., Obrocki P., Wrobel L., Rob M., Takla M., Korolchuk V.I., Rubinsztein D.C. (2025). Autophagy, Aging, and Age-Related Neurodegeneration. Neuron.

[80] Li S., Lei Z., Sun T. (2023). The Role of microRNAs in Neurodegenerative Diseases: A Review. Cell Biol. Toxicol..

[81] Ma Y.M., Zhao L. (2023). Mechanism and Therapeutic Prospect of miRNAs in Neurodegenerative Diseases. Behav. Neurol..

[82] Tu P., Gibon J., Bouron A. (2010). The TRPC6 Channel Activator Hyperforin Induces the Release of Zinc and Calcium from Mitochondria. J. Neurochem..

[83] Sell T.S., Belkacemi T., Flockerzi V., Beck A. (2014). Protonophore Properties of Hyperforin Are Essential for Its Pharmacological Activity. Sci. Rep..

[84] Chen S., Liu X., Peng C., Tan C., Sun H., Liu H., Zhang Y., Wu P., Cui C., Liu C. (2021). The Phytochemical Hyperforin Triggers Thermogenesis in Adipose Tissue via a Dlat–AMPK Signaling Axis to Curb Obesity. Cell Metab..

[85] Cui L., Li S., Wang S., Wu X., Liu Y., Yu W., Wang Y., Tang Y., Xia M., Li B. (2024). Major Depressive Disorder: Hypothesis, Mechanism, Prevention and Treatment. Signal Transduct. Target. Ther..

[86] Sălcudean A., Bodo C.-R., Popovici R.-A., Cozma M.-M., Păcurar M., Crăciun R.-E., Crisan A.-I., Enatescu V.-R., Marinescu I., Cimpian D.-M. (2025). Neuroinflammation—A Crucial Factor in the Pathophysiology of Depression—A Comprehensive Review. Biomolecules.

[87] Lei A.A., Phang V.W.X., Lee Y.Z., Kow A.S.F., Tham C.L., Ho Y.-C., Lee M.T. (2025). Chronic Stress-Associated Depressive Disorders: The Impact of HPA Axis Dysregulation and Neuroinflammation on the Hippocampus—A Mini Review. Int. J. Mol. Sci..

[88] Zhao K., Zhang Y., Yang S., Xiang L., Wu S., Dong J., Li H., Yu H., Hu W. (2025). Neuroinflammation and Stress-Induced Pathophysiology in Major Depressive Disorder: Mechanisms and Therapeutic Implications. Front. Cell. Neurosci..

[89] Wonnemann M., Singer A., Müller W.E. (2000). Inhibition of Synaptosomal Uptake of ^3^H-L-Glutamate and ^3^H-GABA by Hyperforin, a Major Constituent of St. John’s Wort: The Role of Amiloride-Sensitive Sodium Conductive Pathways. Neuropsychopharmacology.

[90] Treiber K., Singer A., Henke B., Müller W.E. (2005). Hyperforin Activates Nonselective Cation Channels (NSCCs). Br. J. Pharmacol..

[91] Leuner K., Li W., Amaral M.D., Rudolph S., Calfa G., Schuwald A.M., Harteneck C., Inoue T., Pozzo-Miller L. (2013). Hyperforin Modulates Dendritic Spine Morphology in Hippocampal Pyramidal Neurons by Activating Ca^2+^-Permeable TRPC6 Channels. Hippocampus.

[92] Li T., Shen F., Li L., Chen P., Zhu Z., Zheng Y., Li H., Suo G., Wang Y., Shi J. (2025). Congenital Hypothyroidism Dysregulates TRPC6 to Mediate Abnormal Dendritic Spine Growth of Hippocampal Neurons. CNS Neurosci. Ther..

[93] Gao X., Chen J., Yin G., Liu Y., Gu Z., Sun R., Sun X., Jiao X., Wang L., Wang N. (2024). Hyperforin Ameliorates Neuroinflammation and White Matter Lesions by Regulating the Microglial VEGFR2/SRC Pathway in Vascular Cognitive Impairment Mice. CNS Neurosci. Ther..

[94] Lee S., Kim J., Kim Y., Kim M., Kang T.C. (2014). Hyperforin Attenuates Microglia Activation and Inhibits p65-Ser276 NF-κB Phosphorylation in the Rat Piriform Cortex Following Status Epilepticus. Neurosci. Res..

[95] Wang H., Shao B., Yu H., Xu F., Wang P., Yu K., Han Y., Song M., Li Y., Cao Z. (2019). Neuroprotective Role of Hyperforin on Aluminum Maltolate-Induced Oxidative Damage and Apoptosis in PC12 Cells and SH-SY5Y Cells. Chem.-Biol. Interact..

[96] Wang Y., Zhang Y., He J., Zhang H., Xiao L., Nazarali A., Zhang Z., Zhang D., Tan Q., Kong J. (2011). Hyperforin Promotes Mitochondrial Function and Development of Oligodendrocytes. J. Neurochem..

[97] Szewczyk B., Pochwat B., Muszyńska B., Opoka W., Krakowska A., Rafało-Ulińska A., Friedland K., Nowak G. (2019). Antidepressant-Like Activity of Hyperforin and Changes in BDNF and Zinc Levels in Mice Exposed to Chronic Unpredictable Mild Stress. Behav. Brain Res..

[98] Crupi R., Mazzon E., Marino A., La Spada G., Bramanti P., Battaglia F., Cuzzocrea S., Spina E. (2011). Hypericum perforatum Treatment: Effect on Behaviour and Neurogenesis in a Chronic Stress Model in Mice. BMC Complement. Altern. Med..

[99] Müller W.E., Singer A., Wonnemann M., Hafner U., Rolli M., Schäfer C. (1998). Hyperforin Represents the Neurotransmitter Reuptake Inhibiting Constituent of *Hypericum* Extract. Pharmacopsychiatry.

[100] Khalil H.M.A., El Henafy H.M.A., Khalil I.A., Bakr A.F., Fahmy M.I., Younis N.S., El-Shiekh R.A. (2023). *Hypericum perforatum* L. Nanoemulsion Mitigates Cisplatin-Induced Chemobrain via Reducing Neurobehavioral Alterations, Oxidative Stress, Neuroinflammation, and Apoptosis in Adult Rats. Toxics.

[101] Woelk H. (2000). Comparison of St John’s Wort and Imipramine for Treating Depression: Randomised Controlled Trial. BMJ.

[102] Schrader E. (2000). Equivalence of St John’s Wort Extract (Ze 117) and Fluoxetine: A Randomized, Controlled Study in Mild–Moderate Depression. Int. Clin. Psychopharmacol..

[103] Szegedi A., Kohnen R., Dienel A., Kieser M. (2005). Acute Treatment of Moderate to Severe Depression with Hypericum Extract WS 5570 (St John’s Wort): Randomised Controlled Double-Blind Non-Inferiority Trial versus Paroxetine. BMJ.

[104] Cui Y.H., Zheng Y. (2016). A Meta-Analysis on the Efficacy and Safety of St John’s Wort Extract in Depression Therapy in Comparison with Selective Serotonin Reuptake Inhibitors in Adults. Neuropsychiatr. Dis. Treat..

[105] Lecrubier Y., Clerc G., Didi R., Kieser M. (2002). Efficacy of St. John’s Wort Extract WS 5570 in Major Depression: A Double-Blind, Placebo-Controlled Trial. Am. J. Psychiatry.

[106] Zhao X., Zhang H., Wu Y., Yu C. (2023). The Efficacy and Safety of St. John’s Wort Extract in Depression Therapy Compared to SSRIs in Adults: A Meta-Analysis of Randomized Clinical Trials. Adv. Clin. Exp. Med..

[107] Ng Q.X., Venkatanarayanan N., Ho C.Y. (2017). Clinical Use of *Hypericum perforatum* (St John’s Wort) in Depression: A Meta-Analysis. J. Affect. Disord..

[108] Kalbermatten N., Saller R. (2025). St John’s Wort (Hypericum perforatum) Fresh Plant Tincture for Patients with Mild to Moderate Depression: A Prospective Observational Study. Complement. Med. Res..

[109] Laakmann G., Schüle C., Baghai T., Kieser M. (1998). St. John’s Wort in Mild to Moderate Depression: The Relevance of Hyperforin for the Clinical Efficacy. Pharmacopsychiatry.

[110] European Medicines Agency, Committee on Herbal Medicinal Products (HMPC) (2022/2023). European Union Herbal Monograph on Hypericum perforatum L., Herba, Revision 1; EMA/HMPC/7695/2021.

[111] Kaehler S.T., Sinner C., Chatterjee S.S., Philippu A. (1999). Hyperforin Enhances the Extracellular Concentrations of Catecholamines, Serotonin and Glutamate in the Rat Locus Coeruleus. Neurosci. Lett..

[112] Langosch J.M., Zhou X.Y., Heinen M., Kupferschmid S., Chatterjee S.S., Nöldner M., Walden J. (2002). St. John’s Wort (*Hypericum perforatum*) Modulates Evoked Potentials in Guinea Pig Hippocampal Slices via AMPA and GABA Receptors. Eur. Neuropsychopharmacol..

[113] Cervo L., Rozio M., Ekalle-Soppo C.B., Guiso G., Morazzoni P., Caccia S. (2002). Role of Hyperforin in the Antidepressant-Like Activity of *Hypericum perforatum* Extracts. Psychopharmacology.

[114] Sevastre-Berghian A.C., Toma V.A., Sevastre B., Hanganu D., Vlase L., Benedec D., Oniga I., Baldea I., Olteanu D., Moldovan R. (2018). Characterization and Biological Effects of *Hypericum* Extracts on Experimentally Induced Anxiety, Oxidative Stress and Inflammation in Rats. J. Physiol. Pharmacol..

[115] Mannel M., Kuhn U., Schmidt U., Ploch M., Murck H. (2010). St. John’s Wort Extract LI160 for the Treatment of Depression with Atypical Features—A Double-Blind, Randomized, and Placebo-Controlled Trial. J. Psychiatr. Res..

[116] Seifritz E., Hatzinger M., Holsboer-Trachsler E. (2016). Efficacy of Hypericum Extract WS^®^ 5570 Compared with Paroxetine in Patients with a Moderate Major Depressive Episode—A Subgroup Analysis. Int. J. Psychiatry Clin. Pract..

[117] Volz H.P., Murck H., Kasper S., Möller H.J. (2002). St. John’s Wort Extract (LI 160) in Somatoform Disorders: Results of a Placebo-Controlled Trial. Psychopharmacology.

[118] Philipp M., Kohnen R., Hiller K.O. (1999). Hypericum Extract versus Imipramine or Placebo in Patients with Moderate Depression: Randomised Multicentre Study of Treatment for Eight Weeks. BMJ.

[119] Xiang Y., Wang L., Gu P., Wang C., Tian Y., Shi W., Deng F., Zhang Y., Gao L., Wang K. (2025). A Randomized, Double-Blind, Positive-Controlled, Multicenter Clinical Trial on the Efficacy and Safety of ShuganJieyu Capsule and St. John’s Wort for Major Depressive Disorder with Somatic Complaints. Int. J. Ment. Health Addict..

[120] Gao F., Peng H., Gou R., Zhou Y., Ren S., Li F. (2025). Exploring Neutrophil Extracellular Traps: Mechanisms of Immune Regulation and Future Therapeutic Potential. Exp. Hematol. Oncol..

[121] Gibon J., Deloulme J.-C., Chevallier T., Ladevèze E., Abrous D.N., Bouron A. (2013). The Antidepressant Hyperforin Increases the Phosphorylation of CREB and the Expression of TrkB in a Tissue-Specific Manner. Int. J. Neuropsychopharmacol..

[122] Chen Y., Zhao X., Kassab R.B., Elhefny M.A., Yang Y., Zheng M., Zhang Q. (2026). Hyperforin Attenuates Scopolamine-Induced Alzheimer’s Pathology in a Rat Model: Abrogation of Caspase-1/11-Mediated Pyroptosis via Inhibition of the HMGB1-Mediated RAGE Pathway. J. Biochem. Mol. Toxicol..

[123] Widy-Tyszkiewicz E., Piechal A., Joniec I., Blecharz-Klin K. (2002). Long-Term Administration of *Hypericum perforatum* Improves Spatial Learning and Memory in the Water Maze. Biol. Pharm. Bull..

[124] Trofimiuk E., Braszko J.J. (2008). Alleviation by *Hypericum perforatum* of the Stress-Induced Impairment of Spatial Working Memory in Rats. Naunyn-Schmiedeberg’s Arch. Pharmacol..

[125] Dinamarca M.C., Cerpa W., Garrido J., Hancke J.L., Inestrosa N.C. (2006). Hyperforin Prevents β-Amyloid Neurotoxicity and Spatial Memory Impairments by Disaggregation of Alzheimer’s Amyloid-β Deposits. Mol. Psychiatry.

[126] Cerpa W., Hancke J.L., Morazzoni P., Bombardelli E., Riva A., Marin P.P., Inestrosa N.C. (2010). The Hyperforin Derivative IDN5706 Occludes Spatial Memory Impairments and Neuropathological Changes in a Double Transgenic Alzheimer’s Mouse Model. Curr. Alzheimer Res..

[127] Inestrosa N.C., Tapia-Rojas C., Griffith T.N., Carvajal F.J., Benito M.J., Rivera-Dictter A., Alvarez A.R., Serrano F.G., Hancke J.L., Burgos P.V. (2011). Tetrahydrohyperforin Prevents Cognitive Deficit, Aβ Deposition, Tau Phosphorylation and Synaptotoxicity in the APPswe/PSEN1ΔE9 Model of Alzheimer’s Disease: A Possible Effect on APP Processing. Transl. Psychiatry.

[128] El Menuawy A., Brüning T., Eiriz I., Hähnel U., Marthe F., Möhle L., Górska A.M., Santos-García I., Wangensteen H., Wu J. (2024). Apolar Extracts of St. John’s Wort Alleviate the Effects of β-Amyloid Toxicity in Early Alzheimer’s Disease. Int. J. Mol. Sci..

[129] Griffith T.N., Varela-Nallar L., Dinamarca M.C., Inestrosa N.C. (2010). Neurobiological Effects of Hyperforin and Its Potential in Alzheimer’s Disease Therapy. Curr. Med. Chem..

[130] Montecinos-Oliva C., Schüller A., Inestrosa N.C. (2015). Tetrahydrohyperforin: A Neuroprotective Modified Natural Compound against Alzheimer’s Disease. Neural Regen. Res..

[131] Gómez del Rio M.A., Sánchez-Reus M.I., Iglesias I., Pozo M.A., García-Arencibia M., Fernández-Ruiz J., García-García L., Delgado M., Benedí J. (2013). Neuroprotective Properties of Standardized Extracts of Hypericum perforatum on a Rotenone Model of Parkinson’s Disease. CNS Neurol. Disord. Drug Targets.

[132] Kiasalari Z., Baluchnejadmojarad T., Roghani M. (2016). Hypericum perforatum Hydroalcoholic Extract Mitigates Motor Dysfunction and Is Neuroprotective in an Intrastriatal 6-Hydroxydopamine Rat Model of Parkinson’s Disease. Cell. Mol. Neurobiol..

[133] Prikhodko V., Chernyuk D., Sysoev Y., Zernov N., Okovityi S., Popugaeva E. (2020). Potential Drug Candidates to Treat TRPC6 Channel Deficiencies in the Pathophysiology of Alzheimer’s Disease and Brain Ischemia. Cells.

[134] Sukumaran P., Sun Y., Schaar A., Selvaraj S., Singh B.B. (2017). TRPC Channels and Parkinson’s Disease. Adv. Exp. Med. Biol..

[135] Hong C., Jeong B., Park H.J., Chung J.Y., Lee J.E., Kim J., Shin Y.-C., So I. (2020). TRP Channels as Emerging Therapeutic Targets for Neurodegenerative Diseases. Front. Physiol..

[136] Zanoli P. (2004). Role of Hyperforin in the Pharmacological Activities of St. John’s Wort. CNS Drug Rev..

[137] Tong H., Yang T., Xu S., Li X., Liu L., Zhou G., Yang S., Yin S., Li X.-J., Li S. (2024). Huntington’s Disease: Complex Pathogenesis and Therapeutic Strategies. Int. J. Mol. Sci..

[138] Mahmoudi M., Rastin M., Arababadi M.K., Anaeigoudari A., Nosratabadi R. (2022). Enhancing the Efficacy of *Hypericum perforatum* in the Treatment of an Experimental Model of Multiple Sclerosis Using Gold Nanoparticles: An In Vivo Study. Avicenna J. Phytomed..

[139] Nosratabadi R., Rastin M., Sankian M., Haghmorad D., Tabasi N., Zamani S., Aghaee A., Salehipour Z., Mahmoudi M. (2016). St. John’s Wort and Its Component Hyperforin Alleviate Experimental Autoimmune Encephalomyelitis through Expansion of Regulatory T-Cells. J. Immunotoxicol..

[140] Nosratabadi R., Rastin M., Sankian M., Haghmorad D., Mahmoudi M. (2016). Hyperforin-Loaded Gold Nanoparticle Alleviates Experimental Autoimmune Encephalomyelitis by Suppressing Th1 and Th17 Cells and Upregulating Regulatory T Cells. Nanomedicine.

[141] Pegoretti V., Swanson K.A., Bethea J.R., Probert L., Eisel U.L.M., Fischer R. (2020). Inflammation and Oxidative Stress in Multiple Sclerosis: Consequences for Therapy Development. Oxid. Med. Cell. Longev..

[142] Al-Khayri J.M., Ravindran M., Banadka A., Vandana C.D., Priya K., Nagella P., Kukkemane K. (2024). Amyotrophic Lateral Sclerosis: Insights and New Prospects in Disease Pathophysiology, Biomarkers and Therapies. Pharmaceuticals.

[143] Cifuentes M., Verdejo H.E., Castro P.F., Corvalan A.H., Ferreccio C., Quest A.F.G., Kogan M.J., Lavandero S. (2025). Low-Grade Chronic Inflammation: A Shared Mechanism for Chronic Diseases. Physiology.

[144] Manful C.F., Fordjour E., Ikumoinein E., Abbey L., Thomas R. (2025). Therapeutic Strategies Targeting Oxidative Stress and Inflammation: A Narrative Review. BioChem.

[145] Dousdampanis P., Aggeletopoulou I., Mouzaki A. (2025). The Role of M1/M2 Macrophage Polarization in the Pathogenesis of Obesity-Related Kidney Disease and Related Pathologies. Front. Immunol..

[146] Zhang J., Cheng F., Rong G., Tang Z., Gui B. (2024). IDN5706 Inhibits Synovial Inflammation via Inducing M2 Polarization of Synovial Macrophages in Osteoarthritis Rats. Pharmacology.

[147] Wang H., Kim S.J., Lei Y., Wang S., Wang H., Huang H., Zhang H., Tsung A. (2024). Neutrophil Extracellular Traps in Homeostasis and Disease. Signal Transduct. Target. Ther..

[148] Dell’Aica I., Niero R., Piazza F., Cabrelle A., Sartor L., Colalto C., Brunetta E., Lorusso G., Benelli R., Albini A. (2007). Hyperforin Blocks Neutrophil Activation of Matrix Metalloproteinase-9, Motility and Recruitment, and Restrains Inflammation-Triggered Angiogenesis and Lung Fibrosis. J. Pharmacol. Exp. Ther..

[149] Cabrelle A., Dell’Aica I., Melchiori L., Carraro S., Brunetta E., Niero R., Scquizzato E., D’Intino G., Calzà L., Garbisa S. (2008). Hyperforin Down-Regulates Effector Function of Activated T Lymphocytes and Shows Efficacy against Th1-Triggered CNS Inflammatory-Demyelinating Disease. J. Leucoc. Biol..

[150] Berköz M., Allahverdiyev O., Yıldırım M. (2018). Investigation of the Effect of Hyperforin and Hypericin on Inflammatory Response in RAW 264.7 Macrophages. Van Med. J..

[151] Xu C., Su W. (2023). Hyperforin Modulates MAPK/CCL11 Signaling to Reduce the Inflammatory Response of Nasal Mucosal Epithelial Cells Caused by Allergic Rhinitis by Targeting BCL6. Exp. Ther. Med..

[152] Arneth B. (2025). Molecular Mechanisms of Immune Regulation: A Review. Cells.

[153] Zhang S., Zhang J., Yu J., Chen X., Zhang F., Wei W., Zhang L., Chen W., Lin N., Wu Y. (2021). Hyperforin Ameliorates Imiquimod-Induced Psoriasis-Like Murine Skin Inflammation by Modulating IL-17A-Producing γδ T Cells. Front. Immunol..

[154] Müller M., Essin K., Hill K., Beschmann H., Rubant S., Schempp C.M., Gollasch M., Boehncke W.H., Harteneck C., Müller W.E. (2008). Specific TRPC6 Channel Activation, a Novel Approach to Stimulate Keratinocyte Differentiation. J. Biol. Chem..

[155] Gendrisch F., Haarhaus B., Krieger N., Quirin K.-W., Schempp C.M., Wölfle U. (2021). The Effect of Herbal Medicinal Products on Psoriasis-Like Keratinocytes. Biomolecules.

[156] Menegazzi M., Novelli M., Beffy P., D’Aleo V., Tedeschi E., Lupi R., Zoratti E., Marchetti P., Suzuki H., Masiello P. (2008). Protective Effects of St. John’s Wort Extract and Its Component Hyperforin against Cytokine-Induced Cytotoxicity in a Pancreatic Beta-Cell Line. Int. J. Biochem. Cell Biol..

[157] Theodorakopoulou A., Pylarinou I., Anastasiou I.A., Tentolouris N. (2025). The Putative Antidiabetic Effect of *Hypericum perforatum* on Diabetes Mellitus. Int. J. Mol. Sci..

[158] Marrelli M., Statti G., Conforti F. (2020). *Hypericum* spp.: An Update on the Biological Activities and Metabolic Profiles. Mini Rev. Med. Chem..

[159] Huang P., Zhu Y., Qin Y., Hu J., Wang J., Qin J. (2025). *Hypericum perforatum* L. Extract Alleviates Metabolic-Associated Fatty Liver Disease through Inflammation, Lipid Metabolism and Ferroptosis Modulation: A Multi-Omics Perspective. Chin. Med..

[160] Mohagheghzadeh A., Badr P., Mohagheghzadeh A., Hemmati S. (2023). *Hypericum perforatum* L. and the Underlying Molecular Mechanisms for Its Choleretic, Cholagogue, and Regenerative Properties. Pharmaceuticals.

[161] Farasati Far B., Gouranmohit G., Naimi-Jamal M.R., Neysani E., El-Nashar H.A.S., El-Shazly M., Khoshnevisan K. (2024). The Potential Role of *Hypericum perforatum* in Wound Healing: A Literature Review on the Phytochemicals, Pharmacological Approaches, and Mechanistic Perspectives. Phytother. Res..

[162] Xu Z., Yang S., Tan Y., Zhang Q., Wang H., Tao J., Liu Q., Wang Q., Feng W., Li Z. (2025). Inflammation in Cardiovascular-Kidney-Metabolic Syndrome: Key Roles and Underlying Mechanisms—A Comprehensive Review. Mol. Cell. Biochem..

[163] Horton W.B., Love K.M., Gregory J.M., Liu Z., Barrett E.J. (2025). Metabolic and Vascular Insulin Resistance: Partners in the Pathogenesis of Cardiovascular Disease in Diabetes. Am. J. Physiol. -Heart Circ. Physiol..

[164] Fan H., He X., Tong H., Chen K. (2024). Preventive Effect of Hyperforin on Lipopolysaccharide-Induced Acute Kidney Injury and Inflammation by Repressing the NF-κB/miR-21 Axis. Cent. Eur. J. Immunol..

[165] Uslusoy F., Nazıroğlu M., Çiğ B. (2017). Inhibition of the TRPM2 and TRPV1 Channels through *Hypericum perforatum* in Sciatic Nerve Injury-Induced Rats Demonstrates Their Key Role in Apoptosis and Mitochondrial Oxidative Stress of Sciatic Nerve and Dorsal Root Ganglion. Front. Physiol..

[166] Benedí J., Arroyo R., Romero C., Martín-Aragón S., Villar A.M. (2004). Antioxidant Properties and Protective Effects of a Standardized Extract of *Hypericum perforatum* on Hydrogen Peroxide-Induced Oxidative Damage in PC12 Cells. Life Sci..

[167] Libby P., Soehnlein O. (2025). Inflammation in Atherosclerosis: Lessons and Therapeutic Implications. Immunity.

[168] Niedźwiedzka-Rystwej P., Siwicka D., Eljaszewicz A. (2024). Exploring the Role of Hyperforin in Modulating the NF-κB/miR-21 Axis in Sepsis-Induced Acute Kidney Injury. Cent. Eur. J. Immunol..

[169] Saki Wang X., He B. (2024). Endothelial Dysfunction: Molecular Mechanisms and Clinical Implications. MedComm.

[170] Novelli M., Masiello P., Beffy P., Menegazzi M. (2020). Protective Role of St. John’s Wort and Its Components Hyperforin and Hypericin against Diabetes through Inhibition of Inflammatory Signaling: Evidence from In Vitro and In Vivo Studies. Int. J. Mol. Sci..

[171] Schäfer A.M., Potterat O., Seibert I., Fertig O., Meyer zu Schwabedissen H.E. (2019). Hyperforin-Induced Activation of the Pregnane X Receptor Is Influenced by the Organic Anion-Transporting Polypeptide 2B1. Mol. Pharmacol..

[172] Lv Y., Luo Y.-Y., Ren H.-W., Li C.-J., Xiang Z.-X., Luan Z.-L. (2022). The Role of Pregnane X Receptor (PXR) in Substance Metabolism. Front. Endocrinol..

[173] Watkins R.E., Maglich J.M., Moore L.B., Wisely G.B., Noble S.M., Davis-Searles P.R., Lambert M.H., Kliewer S.A., Redinbo M.R. (2003). A Crystal Structure of Human PXR in Complex with the St. John’s Wort Compound Hyperforin. Biochemistry.

[174] Moore L.B., Goodwin B., Jones S.A., Wisely G.B., Serabjit-Singh C.J., Willson T.M., Collins J.L., Kliewer S.A. (2000). St. John’s Wort Induces Hepatic Drug Metabolism through Activation of the Pregnane X Receptor. Proc. Natl. Acad. Sci. USA.

[175] Asgary S., Solhpour A., Parkhideh S., Madani H., Mahzouni P., Kabiri N. (2012). Effect of Hydroalcoholic Extract of *Hypericum perforatum* on Selected Traditional and Novel Biochemical Factors of Cardiovascular Diseases and Atherosclerotic Lesions in Hypercholesterolemic Rabbits: A Comparison between the Extract and Lovastatin. J. Pharm. Bioallied Sci..

[176] Feisst C., Werz O. (2004). Suppression of Receptor-Mediated Ca^2+^ Mobilization and Functional Leukocyte Responses by Hyperforin. Biochem. Pharmacol..

[177] Turner L., Wanasinghe A., Brunori P., Santosa S. (2025). Is Adipose Tissue Inflammation the Culprit of Obesity-Associated Comorbidities?. Obes. Rev..

[178] Yan K. (2024). Recent Advances in the Effect of Adipose Tissue Inflammation on Insulin Resistance. Cell. Signal..

[179] Mizuno H., Taketomi A., Nakabayashi T. (2018). Potentially Beneficial Effects of St. John’s Wort (*Hypericum perforatum*) in Patients with Metabolic Syndrome. OBM Integr. Complement. Med..

[180] Richard A.J., Amini Z.J., Ribnicky D.M., Stephens J.M. (2012). St. John’s Wort Inhibits Insulin Signaling in Murine and Human Adipocytes. Biochim. Biophys. Acta.

[181] Amini Z., Boyd B., Doucet J., Ribnicky D.M., Stephens J.M. (2009). St. John’s Wort Inhibits Adipocyte Differentiation and Induces Insulin Resistance in Adipocytes. Biochem. Biophys. Res. Commun..

[182] Hatano T., Sameshima Y., Kawabata M., Yamada S., Shinozuka K., Nakabayashi T., Mizuno H. (2014). St. John’s Wort Promotes Adipocyte Differentiation and Modulates NF-κB Activation in 3T3-L1 Cells. Biol. Pharm. Bull..

[183] Fuller S., Richard A.J., Ribnicky D.M., Beyl R., Mynatt R., Stephens J.M. (2014). St. John’s Wort Has Metabolically Favorable Effects on Adipocytes In Vivo. Evid.-Based Complement Altern. Med..

[184] Zong Y., Li H., Liao P., Chen L., Pan Y., Zheng Y., Zhang C., Liu D., Zheng M., Gao J. (2024). Mitochondrial Dysfunction: Mechanisms and Advances in Therapy. Signal Transduct. Target. Ther..

[185] Liang Q., Ai L., He L., Wang L.C., Wang J.L. (2026). Targeting the AMP-Activated Protein Kinase Pathway: The Active Metabolites of Botanical Drugs Represent Potential Strategies for Treating Metabolic-Associated Fatty Liver Disease. Front. Pharmacol..

[186] Clemente-Suárez V.J., Redondo-Flórez L., Beltrán-Velasco A.I., Martín-Rodríguez A., Yáñez-Sepúlveda R., Tornero-Aguilera J.F. (2023). The Role of Adipokines in Health and Disease. Biomedicines.

[187] Młynarska E., Czarnik W., Dzieża N., Jędraszak W., Majchrowicz G., Prusinowski F., Stabrawa M., Rysz J., Franczyk B. (2025). Type 2 Diabetes Mellitus: New Pathogenetic Mechanisms, Treatment and the Most Important Complications. Int. J. Mol. Sci..

[188] Novelli M., Beffy P., Menegazzi M., De Tata V., Martino L., Sgarbossa A., Porozov S., Pippa A., Masini M., Marchetti P. (2014). St. John’s Wort Extract and Hyperforin Protect Rat and Human Pancreatic Islets against Cytokine Toxicity. Acta Diabetol..

[189] Boire A., Burke K., Cox T.R., Guise T., Jamal-Hanjani M., Janowitz T., Kaplan R., Lee R., Swanton C., Vander Heiden M.G. (2024). Why Do Patients with Cancer Die?. Nat. Rev. Cancer.

[190] Papadopoulou S.K., Poulios E., Pritsa A., Psara E., Migdanis A., Giaginis C. (2026). Natural Products as Promising Pharmacological Agents Against Cancer: A Holistic Overview of Their Anti-Cancer Mechanisms of Action of the Last Five Years. Pharmaceuticals.

[191] Pellarin I., Dall’Acqua A., Favero A., Segatto I., Rossi V., Crestan N., Karimbayli J., Belletti B., Baldassarre G. (2025). Cyclin-Dependent Protein Kinases and Cell Cycle Regulation in Biology and Disease. Signal Transduct. Target. Ther..

[192] Donà M., Dell’Aica I., Pezzato E., Sartor L., Calabrese F., Della Barbera M., Donella-Deana A., Appendino G., Borsarini A., Caniato R. (2004). Hyperforin Inhibits Cancer Invasion and Metastasis. Cancer Res..

[193] Qian S., Long Y., Tan G., Li X., Xiang B., Tao Y., Xie Z., Zhang X. (2024). Programmed Cell Death: Molecular Mechanisms, Biological Functions, Diseases, and Therapeutic Targets. Med. Commun..

[194] Wiechmann K., Müller H., Fischer D., Jauch J., Werz O. (2015). The Acylphloroglucinols Hyperforin and Myrtucommulone A Cause Mitochondrial Dysfunctions in Leukemic Cells by Direct Interference with Mitochondria. Apoptosis.

[195] Jalali P., Shahmoradi A., Samii A., Mazloomnejad R., Hatamnejad M.R., Saeed A., Namdar A., Salehi Z. (2025). The Role of Autophagy in Cancer: From Molecular Mechanism to Therapeutic Window. Front. Immunol..

[196] Kwon J., Oh K.S., Cho S.Y., Bang M.A., Kim H.S., Vaidya B., Kim D. (2016). Estrogenic Activity of Hyperforin in MCF-7 Human Breast Cancer Cells Transfected with Estrogen Receptor. Planta Med..

[197] Nakamura K., Aizawa K., Yamauchi J., Tanoue A. (2013). Hyperforin Inhibits Cell Proliferation and Differentiation in Mouse Embryonic Stem Cells. Cell Prolif..

[198] Barathan M., Zulpa A.K., Mee Hoong S., Vellasamy K.M., Vadivelu J. (2021). Synergistic Effect of Hyperforin and Paclitaxel on Growth Inhibition and Apoptotic Mediator Activation in MCF-7 Human Breast Cancer Cells. J. Taibah Univ. Sci..

[199] Schempp C.M., Kiss J., Kirkin V., Averbeck M., Simon-Haarhaus B., Kremer B., Termeer C.C., Sleeman J., Simon J.C. (2005). Hyperforin Acts as an Angiogenesis Inhibitor. Planta Med..

[200] Imreova P., Feruszova J., Kyzek S., Bodnarova K., Zduriencikova M., Kozics K., Mucaji P., Galova E., Sevcovicova A., Miadokova E. (2017). Hyperforin Exhibits Antigenotoxic Activity on Human and Bacterial Cells. Molecules.

[201] Matić I.Z., Ergün S., Đorđić Crnogorac M., Misir S., Aliyazicioğlu Y., Damjanović A., Džudžević-Čančar H., Stanojković T., Konanç K., Petrović N. (2021). Cytotoxic Activities of *Hypericum perforatum* L. Extracts against 2D and 3D Cancer Cell Models. Cytotechnology.

[202] Šemeláková M., Mikeš J., Jendželovský R., Fedoročko P. (2012). The Pro-Apoptotic and Anti-Invasive Effects of Hypericin-Mediated Photodynamic Therapy Are Enhanced by Hyperforin or Aristoforin in HT-29 Colon Adenocarcinoma Cells. J. Photochem. Photobiol. B.

[203] Rothley M., Schmid A., Thiele W., Schacht V., Plaumann D., Gartner M., Yektaoglu A., Bruyère F., Noël A., Giannis A. (2009). Hyperforin and Aristoforin Inhibit Lymphatic Endothelial Cell Proliferation in Vitro and Suppress Tumor-Induced Lymphangiogenesis in Vivo. Int. J. Cancer.

[204] Quiney C., Billard C., Faussat A.-M., Salanoubat C., Ensaf A., Naït-Si Y., Fourneron J.-D., Kolb J.-P. (2006). Pro-Apoptotic Properties of Hyperforin in Leukemic Cells from Patients with B-Cell Chronic Lymphocytic Leukemia. Leukemia.

[205] Billard C., Merhi F., Bauvois B. (2013). Mechanistic Insights into the Antileukemic Activity of Hyperforin. Curr. Cancer Drug Targets.

[206] Quiney C., Billard C., Salanoubat C., Ensaf A., Naït-Si Y., Fourneron J.-D., Kolb J.-P. (2006). Hyperforin, a New Lead Compound against the Progression of Cancer and Leukemia?. Leukemia.

[207] Albert D., Zündorf I., Dingermann T., Müller W.E., Steinhilber D., Werz O. (2002). Hyperforin Is a Dual Inhibitor of Cyclooxygenase-1 and 5-Lipoxygenase. Biochem. Pharmacol..

[208] Almazrouei K.M., Mishra V., Pandya H., Sambhav K., Bhavsar S.N. (2025). Tumor Microenvironment and Its Role in Cancer Progression: An Integrative Review. Cureus.

[209] Xu J., Tang Z. (2024). Progress on Angiogenic and Antiangiogenic Agents in the Tumor Microenvironment. Front. Oncol..

[210] Majerník M., Jendželovský R., Babinčák M., Košuth J., Ševc J., Tonelli Gombalová Z., Jendželovská Z., Buríková M., Fedoročko P. (2019). Novel Insights into the Effect of Hyperforin and Photodynamic Therapy with Hypericin on Chosen Angiogenic Factors in Colorectal Micro-Tumors Created on Chorioallantoic Membrane. Int. J. Mol. Sci..

[211] Lorusso G., Vannini N., Sogno I., Generoso L., Garbisa S., Noonan D.M., Albini A. (2009). Mechanisms of Hyperforin as an Anti-Angiogenic Angioprevention Agent. Eur. J. Cancer.

[212] Martínez-Poveda B., Verotta L., Bombardelli E., Quesada A.R., Medina M.Á. (2010). Tetrahydrohyperforin and Octahydrohyperforin Are Two New Potent Inhibitors of Angiogenesis. PLoS ONE.

[213] Zhang J., Yao C., Chen J., Zhang Y., Yuan S., Lin Y. (2016). Hyperforin Promotes Post-Stroke Functional Recovery through Interleukin (IL)-17A-Mediated Angiogenesis. Brain Res..

[214] Racacho K.J., Shiau Y.P., Villa R., Mahri S., Tang M., Lin T.Y., Li Y. (2025). The Tumor Immune Microenvironment: Implications for Cancer Immunotherapy, Treatment Strategies, and Monitoring Approaches. Front. Immunol..

[215] Kraus B., Wolff H., Elstner E.F., Heilmann J. (2010). Hyperforin Is a Modulator of Inducible Nitric Oxide Synthase and Phagocytosis in Microglia and Macrophages. Naunyn-Schmiedeberg’s Arch. Pharmacol..

[216] Wang S., Li W., Wang Z., Yang W., Li E., Xia X., Yan F., Chiu S. (2024). Emerging and Reemerging Infectious Diseases: Global Trends and New Strategies for Their Prevention and Control. Signal Transduct. Target. Ther..

[217] Blaskovich M.A.T., Cooper M.A. (2025). Antibiotics Re-Booted—Time to Kick Back against Drug Resistance. npj Antimicrob. Resist..

[218] SeyedAlinaghi S., Mehraeen E., Mirzapour P., Yarmohammadi S., Dehghani S., Zare S., Gholami S., Attarian N., Abiri A., Farahani Rad F. (2025). A Systematic Review on Natural Products with Antimicrobial Potential against WHO’s Priority Pathogens. Eur. J. Med. Res..

[219] Canenguez Benitez J.S., Hernandez T.E., Sundararajan R., Sarwar S., Arriaga A.J., Khan A.T., Matayoshi A., Quintanilla H.A., Kochhar H., Alam M. (2022). Advantages and Disadvantages of Using *St. John’s Wort* as a Treatment for Depression. Cureus.

[220] Schempp C.M., Pelz K., Wittmer A., Schöpf E., Simon J.C. (1999). Antibacterial Activity of Hyperforin from *St. John’s Wort*, against Multiresistant *Staphylococcus aureus* and Gram-Positive Bacteria. Lancet.

[221] Voss A., Verweij P.E. (1999). Antibacterial Activity of Hyperforin from *St. John’s Wort*. Lancet.

[222] Fiebich B.L., Heinrich M., Langosch J.M., Kammerer N., Lieb K. (1999). Antibacterial Activity of Hyperforin from *St. John’s Wort*. Lancet.

[223] Saddiqe Z., Naeem I., Maimoona A. (2010). A Review of the Antibacterial Activity of *Hypericum perforatum* L.. J. Ethnopharmacol..

[224] Süntar I., Oyardı O., Akkol E.K., Özçelik B. (2016). Antimicrobial Effect of the Extracts from *Hypericum perforatum* against Oral Bacteria and Biofilm Formation. Pharm. Biol..

[225] Kakouri E., Daferera D., Trigas P., Charalambous D., Pantelidou M., Tarantilis P.A., Kanakis C.D. (2023). Comparative Study of the Antibacterial Activity, Total Phenolic and Total Flavonoid Content of Nine *Hypericum* Species Grown in Greece. Appl. Sci..

[226] Sherif M.M., Elshikh H.H., Abdel-Aziz M.M., Elaasser M.M., Yosri M. (2023). In Vitro Antibacterial and Phytochemical Screening of *Hypericum perforatum* Extract as Potential Antimicrobial Agents against Multi-Drug-Resistant (MDR) Strains of Clinical Origin. BioMed Res. Int..

[227] Kisa O., Oksuz L., Servi H., Aysal A.I. (2025). Antibacterial Activity of *Hypericum perforatum* L. (*St. John’s Wort*) Extracts against Gram-Positive Bacteria and Characterisation of Its Secondary Metabolites. Nat. Prod. Res..

[228] Schiavone B.I.P., Verotta L., Rosato A., Marilena M., Gibbons S., Bombardelli E., Franchini C., Corbo F. (2014). Anticancer and Antibacterial Activity of Hyperforin and Its Derivatives. Anticancer Agents Med. Chem..

[229] Hussein M., Crawford S., Baker M., Floyd H., Allobawi R., Blaskovich M.A.T., Rao G.G., Zuegg J., Li J., Velkov T. (2025). Hyperforin Potentiates Polymyxin B against Multidrug-Resistant Gram-Negative Pathogens via Membrane Disruption, Biofilm Eradication, and Oxidative Stress. Antimicrob. Agents Chemother..

[230] Roz N., Rehavi M. (2003). Hyperforin Inhibits Vesicular Uptake of Monoamines by Dissipating pH Gradient across Synaptic Vesicle Membrane. Life Sci..

[231] Savage P.B. (2001). Multidrug-Resistant Bacteria: Overcoming Antibiotic Permeability Barriers of Gram-Negative Bacteria. Ann. Med..

[232] Cwikla C., Schmidt K., Matthias A., Bone K.M., Lehmann R., Tiralongo E. (2010). Investigations into the Antibacterial Activities of Phytotherapeutics against *Helicobacter pylori* and *Campylobacter jejuni*. Phytother. Res..

[233] Reichling J., Weseler A., Saller R. (2001). A Current Review of the Antimicrobial Activity of *Hypericum perforatum* L.. Pharmacopsychiatry.

[234] Sotirova Y., Ivanova N., Ermenlieva N., Vilhelmova-Ilieva N., Simeonova L., Metodiev M., Gugleva V., Andonova V. (2025). Antimicrobial and Antiherpetic Properties of Nanoencapsulated *Hypericum perforatum* Extract. Pharmaceuticals.

[235] Giannella M., Lanternier F., Dellière S., Groll A.H., Mueller N.J., Alastruey-Izquierdo A., Slavin M.A. (2025). ECCMID Study Groups on Invasive Fungal Infection and Infection in Immunocompromised Hosts. Invasive Fungal Disease in the Immunocompromised Host: Changing Epidemiology, New Antifungal Therapies, and Management Challenges. Clin. Microbiol. Infect..

[236] Yiu B., Robbins N., Cowen L.E. (2024). Interdisciplinary Approaches for the Discovery of Novel Antifungals. Trends Mol. Med..

[237] Vajs V., Vugdelija S., Trifunović S., Karadžić I., Juranić N., Macura S., Milosavljević S. (2003). Further Degradation Product of Hyperforin from *Hypericum perforatum* (St. John’s Wort). Fitoterapia.

[238] Budantsev A.L., Prikhodko V.A., Varganova I.V., Okovityi S.V. (2021). Biological Activity of *Hypericum perforatum* L. (Hypericaceae): A Review. Pharm. Pharmacol..

[239] Tocci N., D’Auria F.D., Simonetti G., Panella S., Palamara A.T., Debrassi A., Rodrigues C.A., Filho V.C., Sciubba F., Pasqua G. (2013). Bioassay-Guided Fractionation of Extracts from *Hypericum perforatum* In Vitro Roots Treated with Carboxymethylchitosans and Determination of Antifungal Activity against Human Fungal Pathogens. Plant Physiol. Biochem..

[240] Simonetti G., Tocci N., Valletta A., Brasili E., D’Auria F.D., Idoux A., Pasqua G. (2016). In Vitro Antifungal Activity of Extracts Obtained from *Hypericum perforatum* Adventitious Roots Cultured in a Mist Bioreactor against Planktonic Cells and Biofilm of *Malassezia furfur*. Nat. Prod. Res..

[241] Thevissen K., Terras F.R.G., Broekaert W.F. (1999). Permeabilization of Fungal Membranes by Plant Defensins Inhibits Fungal Growth. Appl. Environ. Microbiol..

[242] Thiel G., Rössler O.G. (2017). Hyperforin Activates Gene Transcription Involving Transient Receptor Potential C6 Channels. Biochem. Pharmacol..

[243] Almulhim M., Ghasemian A., Memariani M., Karami F., Yassen A.S.A., Alexiou A., Papadakis M., Batiha G.E. (2025). Drug Repositioning as a Promising Approach for the Eradication of Emerging and Re-Emerging Viral Agents. Mol. Divers..

[244] Mohamed F.F., Anhlan D., Schöfbänker M., Schreiber A., Classen N., Hensel A., Hempel G., Scholz W., Kühn J., Hrincius E.R. (2022). *Hypericum perforatum* and Its Ingredients Hypericin and Pseudohypericin Demonstrate an Antiviral Activity against SARS-CoV-2. Pharmaceuticals.

[245] Fritz D., Venturi C.R., Cargnin S., Schripsema J., Roehe P.M., Montanha J.A., von Poser G.L. (2007). Herpes Virus Inhibitory Substances from *Hypericum connatum* Lam., a Plant Used in Southern Brazil to Treat Oral Lesions. J. Ethnopharmacol..

[246] Pan X., Li J., Sun R., Zhang L., Wang X., Li Y. (2012). Therapeutic Efficacy of *Hypericum perforatum* L. Extract for Mice Infected with an Influenza A Virus. Can. J. Physiol. Pharmacol..

[247] Akram M., Tahir I.M., Mehboob H. (2018). Antiviral Potential of Medicinal Plants against HIV, HSV, Influenza, Hepatitis, and Coxsackievirus: A Systematic Review. Phytother. Res..

[248] Raczkiewicz I., Rivière C., Bouquet P., Desmarets L., Tarricone A., Camuzet C., François N., Lefèvre G., Silva Angulo F., Robil C. (2024). Hyperforin, the Major Metabolite of *St. John’s Wort*, Exhibits Pan-Coronavirus Antiviral Activity. Front. Microbiol..

[249] Birt D.F., Widrlechner M.P., Hammer K.D.P., Hillwig M.L., Wei J., Kraus G.A., Murphy P.A., McCoy J., Wurtele E.S., Neighbors J.D. (2009). Hypericum in Infection: Identification of Anti-Viral and Anti-Inflammatory Constituents. Pharm. Biol..

[250] Bajrai L.H., El-Kafrawy S.A., Hassan A.M., Alandijany T.A., Alzahrani A.J., Al-Qahtani A.A., Azhar E.I., Damanhouri G.A., Abuzenadah A.M., Hakami A.R. (2022). In Vitro Screening of Anti-Viral and Virucidal Effects against SARS-CoV-2 by *Hypericum perforatum* and *Echinacea*. Sci. Rep..

[251] Maury W., Price J.P., Brindley M.A., Oh C., Neighbors J.D., Wiemer D.F., Wiemer A.J., Wills J.W. (2009). Identification of Light-Independent Inhibition of Human Immunodeficiency Virus-1 Infection through Bioguided Fractionation of *Hypericum perforatum*. Virol. J..

[252] Masiello P., Novelli M., Beffy P., Menegazzi M. (2020). Can *Hypericum perforatum* (SJW) Prevent Cytokine Storm in COVID-19 Patients?. Phytother. Res..

[253] Marceau T., Braibant M. (2024). Role of Viral Envelope Proteins in Determining Susceptibility of Viruses to IFITM Proteins. Viruses.

[254] Tang J., Colacino J.M., Larsen S.H., Spitzer W. (1990). Virucidal Activity of Hypericin against Enveloped and Non-Enveloped DNA and RNA Viruses. Antivir. Res..

[255] Martinotti S., Ranzato E. (2025). Targeting the Unfolded Protein Response with Natural Products: Therapeutic Potential in ER Stress-Related Diseases. Int. J. Mol. Sci..

[256] Angelini P. (2024). Plant-Derived Antimicrobials and Their Crucial Role in Combating Antimicrobial Resistance. Antibiotics.

[257] Tesema M.Y. (2025). Alternative Approaches to Combat Antimicrobial Resistance: A Silent Pandemic Outbreak Concern. Discov. Med..

[258] Hübner A.T., Pohl M., Reinhold D., Bräuer R., Göbel U.B., Lindequist U. (2003). Treatment with *Hypericum perforatum* L. Does Not Trigger Decreased Resistance in *Staphylococcus aureus* against Antibiotics and Hyperforin. Phytomedicine.

[259] Schiavone B.I., Rosato A., Marilena M., Gibbons S., Bombardelli E., Verotta L., Franchini C., Corbo F. (2013). Biological Evaluation of Hyperforin and Its Hydrogenated Analogue on Bacterial Growth and Biofilm Production. J. Nat. Prod..

[260] Jeong G.-J., Khan F., Tabassum N., Cho K.-J., Kim Y.-M. (2024). Strategies for Controlling Polymicrobial Biofilms: A Focus on Antibiofilm Agents. Int. J. Antimicrob. Agents.

[261] Gobbi M., Moia M., Funicello M., Riva A., Morazzoni P., Mennini T. (2004). In Vitro Effects of the Dicyclohexylammonium Salt of Hyperforin on Interleukin-6 Release in Different Experimental Models. Planta Med..

[262] McKay D.M., Defaye M., Rajeev S., MacNaughton W.K., Nasser Y., Sharkey K.A. (2024). Neuroimmunophysiology of the Gastrointestinal Tract. Am. J. Physiol.-Gastrointest. Liver Physiol..

[263] Mostafavi Abdolmaleky H., Zhou J.-R. (2024). Gut Microbiota Dysbiosis, Oxidative Stress, Inflammation, and Epigenetic Alterations in Metabolic Diseases. Antioxidants.

[264] Jiang Z.-M., Wang F.-F., Zhao Y.-Y., Lu L.-F., Jiang X.-Y., Huang T.-Q., Lin Y., Guo L., Weng Z.-B., Liu E.-H. (2024). *Hypericum perforatum* L. Attenuates Depression by Regulating *Akkermansia muciniphila*, Tryptophan Metabolism, and the NFκB–NLRP2–Caspase-1–IL-1β Pathway. Phytomedicine.

[265] Matar A., Damianos J.A., Jencks K.J., Camilleri M. (2024). Intestinal Barrier Impairment, Preservation, and Repair: An Update. Nutrients.

[266] Aburto M.R., Cryan J.F. (2024). Gastrointestinal and Brain Barriers: Unlocking Gates of Communication across the Microbiota–Gut–Brain Axis. Nat. Rev. Gastroenterol. Hepatol..

[267] Horowitz A., Chanez-Paredes S.D., Haest X., Turner J.R. (2023). Paracellular Permeability and Tight Junction Regulation in Gut Health and Disease. Nat. Rev. Gastroenterol. Hepatol..

[268] Di Vincenzo F., Del Gaudio A., Petito V., Lopetuso L.R., Scaldaferri F. (2024). Gut Microbiota, Intestinal Permeability, and Systemic Inflammation: A Narrative Review. Intern. Emerg. Med..

[269] Sahoo D.K., Heilmann R.M., Paital B., Patel A., Yadav V.K., Wong D., Jergens A.E. (2023). Oxidative Stress, Hormones, and Effects of Natural Antioxidants on Intestinal Inflammation in Inflammatory Bowel Disease. Front. Endocrinol..

[270] Zhang R., Perekatt A., Chen L. (2024). Metabolic Regulation of Intestinal Homeostasis: Molecular and Cellular Mechanisms and Diseases. MedComm.

[271] Takada H., Yonekawa J., Matsumoto M., Furuya K., Sokabe M. (2017). Hyperforin/HP-β-Cyclodextrin Enhances Mechanosensitive Ca^2+^ Signaling in HaCaT Keratinocytes and in Atopic Skin Ex Vivo Which Accelerates Wound Healing. BioMed Res. Int..

[272] Kim J.E., Park H., Choi S.H., Kong M.J., Kang T.C. (2019). TRPC6-Mediated ERK1/2 Activation Increases Dentate Granule Cell Resistance to Status Epilepticus via Regulating Lon Protease-1 Expression and Mitochondrial Dynamics. Cells.

[273] Neurath M.F. (2024). Strategies for Targeting Cytokines in Inflammatory Bowel Disease. Nat. Rev. Immunol..

[274] Schempp C.M., Winghofer B., Lüdtke R., Simon-Haarhaus B., Schöpf E., Simon J.C. (2000). Topical Application of *St. John’s Wort* (*Hypericum perforatum* L.) and of Its Metabolite Hyperforin Inhibits the Allostimulatory Capacity of Epidermal Cells. Br. J. Dermatol..

[275] Ross F.C., Patangia D., Grimaud G., Lavelle A., Dempsey E.M., Ross R.P., Stanton C. (2024). The Interplay between Diet and the Gut Microbiome: Implications for Health and Disease. Nat. Rev. Microbiol..

[276] Zhang Z., Yao C., Li M., Wang L.C., Huang W., Chen Q.J. (2022). Prophylactic Effects of Hyperforin on Anhedonia-Like Phenotype in a Chronic Restraint Stress Model: A Role of Gut Microbiota. Lett. Appl. Microbiol..

[277] Zhang Z., Xing B., Liu X., Shi K., Chen Q. (2025). Hyperforin-Induced Gut Microbiota Metabolite Carbocysteine Protects against Depressive-Like Behaviors in Mice by Modulating the Colonic Mucus Barrier. J. Affect. Disord..

[278] Chauveau A., Treyer A., Geirnaert A., Bircher L., Babst A., Abegg V.F., Simões-Wüst A.P., Lacroix C., Potterat O., Hamburger M. (2023). Intestinal Permeability and Gut Microbiota Interactions of Pharmacologically Active Compounds in Valerian and St. John’s Wort. Biomed. Pharmacother..

[279] Grafakou M.E., Pferschy-Wenzig E.M., Aziz-Kalbhenn H., Kelber O., Moissl-Eichinger C., Bauer R. (2025). Bidirectional Interactions between St. John’s Wort and Gut Microbiome: Potential Implications on Gut-Brain-Axis. Biomed. Pharmacother..

[280] Mayer E.A., Ryu H.J., Bhatt R.R. (2023). The Neurobiology of Irritable Bowel Syndrome. Mol. Psychiatry.

[281] Zhou C., Tabb M.M., Sadatrafiei A., Grün F., Sun A., Blumberg B. (2004). Hyperforin, the Active Component of *St. John’s Wort*, Induces IL-8 Expression in Human Intestinal Epithelial Cells via a MAPK-Dependent, NF-κB-Independent Pathway. J. Clin. Immunol..

[282] Dost T., Ozkayran H., Gokalp F., Yenisey C., Birincioglu M. (2009). The Effect of *Hypericum perforatum* (St. John’s Wort) on Experimental Colitis in Rats. Dig. Dis. Sci..

[283] Demirci B., Dost T. (2025). Effect of *St. John’s Wort* (*Hypericum perforatum* L.) on Colonic Inflammation and Tissue Damage in a Rat Model of TNBS-Induced Colitis. Indian J. Exp. Biol..

[284] Sofi S.H., Nuraddin S.M., Amin Z.A., Al-Bustany H.A., Nadir M.Q. (2020). Gastroprotective Activity of *Hypericum perforatum* Extract in Ethanol-Induced Gastric Mucosal Injury in Wistar Rats: A Possible Involvement of H+/K+-ATPase α Inhibition. Heliyon.

[285] Cayci M.K., Dayioglu H. (2009). *Hypericum perforatum* Extracts Healed Gastric Lesions Induced by Hypothermic Restraint Stress in Wistar Rats. Saudi Med. J..

[286] Arsić I., Žugić A., Antić D.R., Zdunić G., Dekanski D., Marković G., Tadić V. (2010). *Hypericum perforatum* L. Hypericaceae/Guttiferae Sunflower, Olive and Palm Oil Extracts Attenuate Cold Restraint Stress—Induced Gastric Lesions. Molecules.

[287] Cioce A., Cavani A., Cattani C., Scopelliti F. (2024). Role of the Skin Immune System in Wound Healing. Cells.

[288] Leuner K., Kraus M., Woelfle U., Beschmann H., Harteneck C., Boehncke W.H., Schempp C.M., Müller W.E. (2011). Reduced TRPC Channel Expression in Psoriatic Keratinocytes Is Associated with Impaired Differentiation and Enhanced Proliferation. PLoS ONE.

[289] Breijyeh Z., Karaman R. (2024). Antibacterial Activity of Medicinal Plants and Their Role in Wound Healing. Future J. Pharm. Sci..

[290] Sotirova Y., Kiselova-Kaneva Y., Vankova D., Tasinov O., Ivanova D., Popov H., Hristova M., Nikolova K., Andonova V. (2024). Tissue Regeneration and Remodeling in Rat Models after Application of *Hypericum perforatum* L. Extract-Loaded Bigels. Gels.

[291] El-Elimat T., El-Qaderi H.S., Hananeh W.M., Abu AlSamen M.M., Al Sharie A.H., Alshehabat M.A., Al-Gharaibeh M., Alali F.Q. (2023). Evaluation of the Wound Healing Potential of *Hypericum triquetrifolium* Turra: An Experimental Animal Study and Histopathological Examination. Sci. Pharm..

[292] Süntar I., Akkol E.K., Keleş H., Oktem A., Başer K.H.C., Yeşilada E. (2011). A Novel Wound Healing Ointment: A Formulation of *Hypericum perforatum* Oil and Sage and Oregano Essential Oils Based on Traditional Turkish Knowledge. J. Ethnopharmacol..

[293] Arsić I., Žugić A., Tadić V., Tasić-Kostov M., Mišić D., Primorac M., Runjaić-Antić D. (2012). Estimation of Dermatological Application of Creams with St. John’s Wort Oil Extracts. Molecules.

[294] Baker P., Huang C., Radi R., Moll S.B., Jules E., Arbiser J.L. (2023). Skin Barrier Function: The Interplay of Physical, Chemical, and Immunologic Properties. Cells.

[295] Lefèvre-Utile A., Braun C., Haftek M., Aubin F. (2021). Five Functional Aspects of the Epidermal Barrier. Int. J. Mol. Sci..

[296] Meinke M.C., Schanzer S., Haag S.F., Casetti F., Müller M.L., Wölfle U., Kleemann A., Lademann J., Schempp C.M. (2012). In Vivo Photoprotective and Anti-Inflammatory Effect of Hyperforin Is Associated with High Antioxidant Activity In Vitro and Ex Vivo. Eur. J. Pharm. Biopharm..

[297] Füller J., Kellner T., Gaid M., Beerhues L., Müller-Goymann C.C. (2018). Stabilization of Hyperforin Dicyclohexylammonium Salt with Dissolved Albumin and Albumin Nanoparticles for Studying Hyperforin Effects on 2D Cultivation of Keratinocytes In Vitro. Eur. J. Pharm. Biopharm..

[298] Kvedariene V., Vaskovic M., Semyte J.B. (2025). Role of Oxidative Stress and Antioxidants in the Course of Atopic Dermatitis. Int. J. Mol. Sci..

[299] Medina M.A., Martínez-Poveda B., Amores-Sánchez M.I., Quesada A.R. (2006). Hyperforin: More than an Antidepressant Bioactive Compound?. Life Sci..

[300] El-Sherbeni S.A., Negm W.A. (2023). The Wound Healing Effect of Botanicals and Pure Natural Substances Used in In Vivo Models. Inflammopharmacology.

[301] Cao B., Xu Q., Shi Y., Zhao R., Li H., Zheng J., Liu F., Wan Y., Wei B. (2024). Pathology of Pain and Its Implications for Therapeutic Interventions. Signal Transduct. Target. Ther..

[302] Galeotti N., Vivoli E., Bilia A.R., Vincieri F.F., Ghelardini C. (2010). *St. John’s Wort* Reduces Neuropathic Pain through a Hypericin-Mediated Inhibition of Protein Kinase C γ and ε Activity. Biochem. Pharmacol..

[303] D’Egidio F., Lombardozzi G., Kacem Ben Haj M’Barek H.E., Mastroiacovo G., Alfonsetti M., Cimini A. (2022). The Influence of Dietary Supplementations on Neuropathic Pain. Life.

[304] Stojanović N.M., Radulović N.S., Mitić K.V., Randjelović P.J., Stojanović-Radić Z.Z., Laketić D. (2016). Antinociceptive Properties of *St. John’s Wort* (*Hypericum perforatum*) and Other *Hypericum* Species. Nat. Prod. Commun..

[305] Chen H., Xiong R., Cheng J., Ye J., Qiu Y., Huang S., Li M., Liu Z., Pang J., Zhang X. (2024). Effects and Mechanisms of Polyunsaturated Fatty Acids on Age-Related Musculoskeletal Diseases: Sarcopenia, Osteoporosis, and Osteoarthritis—A Narrative Review. Nutrients.

[306] Zhang J., Wang S., Rong G., Cheng F., Gui B., Shen C. (2018). Tetrahydrohyperforin Prevents Articular Cartilage Degeneration and Affects Autophagy in Rats with Osteoarthritis. Exp. Ther. Med..

[307] You M.K., Kim D.W., Jeong K.S., Bang M.A., Kim H.S., Rhuy J., Kim H.A. (2015). *St. John’s Wort* (*Hypericum perforatum*) Stimulates Human Osteoblastic MG-63 Cell Proliferation and Attenuates Trabecular Bone Loss Induced by Ovariectomy. Nutr. Res. Pract..

[308] López-Otín C., Blasco M.A., Partridge L., Serrano M., Kroemer G. (2023). Hallmarks of Aging: An Expanding Universe. Cell.

[309] Zhou Y., Nan F., Zhang Q., Xu W., Fang S., Liu K., Zhao B., Han H., Xie X., Qin C. (2024). Natural Products That Alleviate Depression: The Putative Role of Autophagy. Pharmacol. Ther..

[310] Kasper S., Anghelescu I.G., Szegedi A., Dienel A., Kieser M. (2006). Superior Efficacy of St John’s Wort Extract WS 5570 Compared to Placebo in Patients with Major Depression: A Randomized, Double-Blind, Placebo-Controlled, Multi-Center Trial [ISRCTN77277298]. BMC Med..

[311] Melzer J., Brignoli R., Keck M.E., Saller R. (2010). A Hypericum Extract in the Treatment of Depressive Symptoms in Outpatients: An Open Study. Res. Complement. Med..

[312] Sadeghi A., Ghorayshi F., Baghshahi H., Akbari H., Memarzadeh M.R., Taghizadeh M., Safaei A. (2023). The Antidepressant Effect of Combined Extracts of *Hypericum perforatum* and *Echium amoenum* Supplementation in Patients with Depression Symptoms: A Randomized Clinical Trial. Avicenna J. Phytomed..

[313] Kasper S., Volz H.-P., Möller H.J., Dienel A., Kieser M. (2008). Continuation and Long-Term Maintenance Treatment with *Hypericum* Extract WS 5570 after Recovery from an Acute Episode of Moderate Depression—A Double-Blind, Randomized, Placebo-Controlled Long-Term Trial. Eur. Neuropsychopharmacol..

[314] Shelton R.C., Keller M.B., Gelenberg A., Dunner D.L., Hirschfeld R., Thase M.E., Russell J., Lydiard R.B., Crits-Cristoph P., Gallop R. (2001). Effectiveness of St John’s Wort in Major Depression: A Randomized Controlled Trial. JAMA.

[315] Rahman S.Z., Basilakis J., Rahmadi A., Lujic S., Musgrave I., Jorm L., Hay P., Münch G. (2013). Use of Serotonergic Antidepressants and St John’s Wort in Older Australians: A Population-Based Cohort Study. Australas. Psychiatry.

[316] Sarris J., Fava M., Schweitzer I., Mischoulon D. (2012). St John’s Wort (*Hypericum perforatum*) versus Sertraline and Placebo in Major Depressive Disorder: Continuation Data from a 26-Week Randomized Controlled Trial. Pharmacopsychiatry.

[317] Apaydin E.A., Maher A.R., Shanman R., Booth M.S., Miles J.N.V., Sorbero M.E., Hempel S. (2016). A Systematic Review of St. John’s Wort for Major Depressive Disorder. Syst. Rev..

[318] Linde K., Berner M., Egger M., Mulrow C. (2005). St John’s Wort for Depression: Meta-Analysis of Randomised Controlled Trials. Br. J. Psychiatry.

[319] Linde K., Berner M.M., Kriston L. (2008). St John’s Wort for Major Depression. Cochrane Database Syst. Rev..

[320] Whiskey E., Werneke U., Taylor D. (2001). A Systematic Review and Meta-Analysis of *Hypericum perforatum* in Depression: A Comprehensive Clinical Review. Int. Clin. Psychopharmacol..

[321] Rahimi R., Nikfar S., Abdollahi M. (2009). Efficacy and Tolerability of *Hypericum perforatum* in Major Depressive Disorder in Comparison with Selective Serotonin Reuptake Inhibitors: A Meta-Analysis. Prog. Neuropsychopharmacol. Biol. Psychiatry.

[322] Vorbach E.-U., Hübner W.-D., Arnoldt K.-H. (1994). Effectiveness and Tolerance of the Hypericum Extract LI 160 in Comparison with Imipramine: Randomized Double-Blind Study with 135 Outpatients. J. Geriatr. Psychiatry Neurol..

[323] Bitran S., Farabaugh A.H., Ameral V.E., LaRocca R.A., Clain A.J., Fava M., Mischoulon D. (2011). Do Early Changes in the HAM-D-17 Anxiety/Somatization Factor Items Affect the Treatment Outcome among Depressed Outpatients? Comparison of Two Controlled Trials of St John’s Wort (*Hypericum perforatum*) versus a SSRI. Int. Clin. Psychopharmacol..

[324] Kobak K.A., Taylor L.V.H., Warner G., Futterer R. (2005). St. John’s Wort versus Placebo in Social Phobia: Results from a Placebo-Controlled Pilot Study. J. Clin. Psychopharmacol..

[325] Sarris J., Kavanagh D.J., Deed G., Bone K.M. (2009). St. John’s Wort and Kava in Treating Major Depressive Disorder with Comorbid Anxiety: A Randomised Double-Blind Placebo-Controlled Pilot Trial. Hum. Psychopharmacol..

[326] Yechiam E., Ben-Eliezer D., Ashby N.J.S., Bar-Shaked M. (2019). The Acute Effect of *Hypericum perforatum* on Short-Term Memory in Healthy Adults. Psychopharmacology.

[327] Johnson D., Ksciuk H., Woelk H., Sauerwein-Giese E., Frauendorf A. (1994). Effects of *Hypericum* Extract LI 160 Compared with Maprotiline on Resting EEG and Evoked Potentials in 24 Volunteers. J. Geriatr. Psychiatry Neurol..

[328] Valvassori S.S., Borges C., Bavaresco D.V., Varela R.B., Resende W.R., Peterle B.R., Arent C.O., Budni J., Quevedo J. (2018). *Hypericum perforatum* Chronic Treatment Affects Cognitive Parameters and Brain Neurotrophic Factor Levels. Braz. J. Psychiatry.

[329] Lee J., Lee S., Jo W., Ji H.W., Pyeon M., Moon M., Yun J., Lee J.H., Sohn S.-O. (2024). Effect of a Salvia officinalis and Hypericum perforatum Mixture on Improving Memory and Cognitive Decline. Adv. Tradit. Med..

[330] Alzoubi K.H., Abdel-Hafiz L., Khabour O.F., El-Elimat T., Alzubi M.A., Alali F.Q. (2020). Evaluation of the Effect of *Hypericum triquetrifolium* Turra on Memory Impairment Induced by Chronic Psychosocial Stress in Rats: Role of BDNF. Drug Des. Dev. Ther..

[331] Trofimiuk E., Hołownia A., Braszko J.J. (2011). St. John’s Wort May Relieve Negative Effects of Stress on Spatial Working Memory by Changing Synaptic Plasticity. Naunyn-Schmiedeberg’s Arch. Pharmacol..

[332] Schempp C.M., Windeck T., Hezel S., Simon J.C. (2003). Topical Treatment of Atopic Dermatitis with St. John’s Wort Cream—A Randomized, Placebo-Controlled, Double-Blind Half-Side Comparison. Phytomedicine.

[333] Samadi S., Khadivzadeh T., Emami A., Sanjar Moosavi N., Tafaghodi M., Behnam H.R. (2010). The Effect of *Hypericum perforatum* on the Wound Healing and Scar of Cesarean. J. Altern. Complement. Med..

[334] Haag S.F., Tscherch K., Arndt S., Kleemann A., Gersonde I., Lademann J., Rohn S., Meinke M.C. (2014). Enhancement of Skin Radical Scavenging Activity and Stratum Corneum Lipids after the Application of a Hyperforin-Rich Cream. Eur. J. Pharm. Biopharm..

[335] Estrada M., Chávez-Nomberto R.E., Tsuneto A.P., Crestani A.S., Awad C., Chone C.T., Kimura D., Quiroz Robladillo D., Lopes E.A., Abdullah H.I. (2023). Anti-Inflammatory Effects of Topical *Hypericum perforatum*: A Systematic Review. Princ. Pract. Clin. Res..

[336] Masopust J., Protopopová D., Vališ M., Pavelek Z., Klímová B. (2018). Treatment of Behavioral and Psychological Symptoms of Dementias with Psychopharmaceuticals: A Review. Neuropsychiatr. Dis. Treat..

[337] Käufeler R., Meier B., Brattström A. (2001). Efficacy and Tolerability of Ze 117 St. John’s Wort Extract in Comparison with Placebo, Imipramine and Fluoxetine for the Treatment of Mild to Moderate Depression According to ICD-10: An Overview. Pharmacopsychiatry.

[338] Linde K., Ramirez G., Mulrow C.D., Pauls A., Weidenhammer W., Melchart D. (1996). St John’s Wort for Depression—An Overview and Meta-Analysis of Randomized Clinical Trials. BMJ.

[339] Kasper S., Gastpar M., Möller H.-J., Müller W.E., Volz H.-P., Dienel A., Kieser M. (2010). Better Tolerability of St. John’s Wort Extract WS 5570 Compared to Treatment with SSRIs: A Reanalysis of Data from Controlled Clinical Trials in Acute Major Depression. Int. Clin. Psychopharmacol..

[340] Staudinger J.L., Ding X., Lichti K. (2006). Pregnane X Receptor and Natural Products: Beyond Drug–Drug Interactions. Expert Opin. Drug Metab. Toxicol..

[341] Kandel B.A., Ekins S., Leuner K., Thasler W.E., Harteneck C., Zanger U.M. (2014). No Activation of Human Pregnane X Receptor by Hyperforin-Related Phloroglucinols. J. Pharmacol. Exp. Ther..

[342] Solomon D., Adams J., Graves N. (2013). Economic Evaluation of St. John’s Wort (*Hypericum perforatum*) for the Treatment of Mild to Moderate Depression. J. Affect. Disord..

[343] Solomon D., Ford E., Adams J., Graves N. (2011). Potential of St John’s Wort for the Treatment of Depression: The Economic Perspective. Aust. N. Z. J. Psychiatry.

[344] Chen Q., Cheng H.J., Bian Z.X., Lyu A.P., Yang Y., Chan K.W. (2025). Regulatory Frameworks and Evidence Requirements for Traditional, Complementary and Integrative Medicines. Bull. World Health Organ..

[345] Bodrogi J., Kaló Z. (2010). Principles of Pharmacoeconomics and Their Impact on Strategic Imperatives of Pharmaceutical Research and Development. Br. J. Pharmacol..

[346] Russo E., Scicchitano F., Whalley B.J., Mazzitello C., Ciriaco M., Esposito S., Patanè M., Upton R., Pugliese M., Chimirri S. (2014). Hypericum perforatum: Pharmacokinetic, Mechanism of Action, Tolerability, and Clinical Drug–Drug Interactions. Phytother. Res..

[347] Izzo A.A. (2012). Interactions between Herbs and Conventional Drugs: Overview of the Clinical Data. Med. Princ. Pract..

[348] Izzo A.A., Ernst E. (2009). Interactions between Herbal Medicines and Prescribed Drugs: An Updated Systematic Review. Drugs.

[349] Henderson L., Yue Q.Y., Bergquist C., Gerden B., Arlett P. (2002). St John’s Wort (*Hypericum perforatum*): Drug Interactions and Clinical Outcomes. Br. J. Clin. Pharmacol..

[350] Butterweck V., Schmidt M. (2007). St. John’s Wort: Role of Active Compounds for Its Mechanism of Action and Efficacy. Wien. Med. Wochenschr..

[351] Di Carlo G., Borrelli F., Ernst E., Izzo A.A. (2001). St John’s Wort: Prozac from the Plant Kingdom. Trends Pharmacol. Sci..

[352] Whitten D.L., Myers S.P., Hawrelak J.A., Wohlmuth H. (2006). The Effect of St John’s Wort Extracts on CYP3A: A Systematic Review of Prospective Clinical Trials. Br. J. Clin. Pharmacol..

[353] Stevinson C., Ernst E. (1999). Safety of Hypericum in Patients with Depression. Mol. Diagn. Ther..

[354] Greeson J.M., Sanford B., Monti D.A. (2001). St. John’s Wort (*Hypericum perforatum*): A Review of the Current Pharmacological, Toxicological, and Clinical Literature. Psychopharmacology.

[355] Barnes J., Anderson L.A., Phillipson J.D. (2001). St John’s Wort (*Hypericum perforatum* L.): A Review of Its Chemistry, Pharmacology and Clinical Properties. J. Pharm. Pharmacol..

[356] Anghelescu I.G., Kohnen R., Szegedi A., Klement S., Kieser M. (2006). Comparison of Hypericum Extract WS 5570 and Paroxetine in Ongoing Treatment after Recovery from an Episode of Moderate to Severe Depression: Results from a Randomized Multicenter Study. Pharmacopsychiatry.

[357] Brenner R., Azbel V., Madhusoodanan S., Pawlowska M. (2000). Comparison of an Extract of Hypericum (LI 160) and Sertraline in the Treatment of Depression: A Double-Blind, Randomized Pilot Study. Clin. Ther..

[358] van Gurp G., Meterissian G.B., Haiek L.N., McCusker J., Bellavance F. (2002). St John’s Wort or Sertraline? Randomized Controlled Trial in Primary Care. Can. Fam. Physician.

[359] Uebelhack R., Gruenwald J., Graubaum H.J., Busch R. (2004). Efficacy and Tolerability of Hypericum Extract STW 3-VI in Patients with Moderate Depression: A Double-Blind, Randomized, Placebo-Controlled Clinical Trial. Adv. Ther..

[360] Brattström A. (2009). Long-Term Effects of St. John’s Wort (*Hypericum perforatum*) Treatment: A 1-Year Safety Study in Mild to Moderate Depression. Phytomedicine.

[361] Knüppel L., Linde K. (2004). Adverse Effects of St. John’s Wort: A Systematic Review. J. Clin. Psychiatry.

[362] Chrubasik-Hausmann S., Vlachojannis J., McLachlan A.J. (2019). Understanding Drug Interactions with St John’s Wort (*Hypericum perforatum* L.): Impact of Hyperforin Content. J. Pharm. Pharmacol..

[363] Mueller S.C., Majcher-Peszynska J., Uehleke B., Klammt S., Mundkowski R.G., Miekisch W., Sievers H., Bauer S., Frank B., Kundt G. (2006). The Extent of Induction of CYP3A by St. John’s Wort Varies among Products and Is Linked to Hyperforin Dose. Eur. J. Clin. Pharmacol..

[364] Negreş S., Scutari C., Ionică F.E., Gonciar V., Velescu B.Ş., Şeremet O.C., Zanfirescu A., Zbârcea C.E., Ştefănescu E., Ciobotaru E. (2016). Influence of Hyperforin on the Morphology of Internal Organs and Biochemical Parameters, in Experimental Model in Mice. Rom. J. Morphol. Embryol..

[365] Biber A., Fischer H., Römer A., Chatterjee S.S. (1998). Oral Bioavailability of Hyperforin from *Hypericum* Extracts in Rats and Human Volunteers. Pharmacopsychiatry.

[366] Jabbar A.A.J., Alamri Z.Z. (2024). Acute Toxicity and Hepatoprotective Influence of *Hypericum perforatum* Against Thioacetamide-Induced Liver Fibrosis in Rats. J. Evid.-Based Integr. Med..

[367] Skalisz L.L., Beijamini V., Andreatini R. (2004). Effect of *Hypericum perforatum* on Marble-Burying by Mice. Phytother. Res..

[368] Steenkamp V., Parkar H., Dasgupta A. (2023). Utility of Therapeutic Drug Monitoring in Identifying Clinically Significant Interactions Between St. John’s Wort and Prescription Drugs. Ther. Drug Monit..

[369] Hübner W.-D., Arnoldt K.-H. (2000). St. John’s Wort: A One-Year Treatment Study [in German; English Abstract]. Z. Phytother..

[370] Trautmann-Sponsel R.D., Dienel A. (2004). Safety of Hypericum Extract in Mildly to Moderately Depressed Outpatients: A Review Based on Data from Three Randomized, Placebo-Controlled Trials. J. Affect. Disord..

[371] Kalb R., Trautmann-Sponsel R.D., Kieser M. (2001). Efficacy and Tolerability of Hypericum Extract WS 5572 versus Placebo in Mildly to Moderately Depressed Patients: A Randomized Double-Blind Multicenter Clinical Trial. Pharmacopsychiatry.

[372] Guefack M.-G.F., Damen F., Ngaffo C.M.N., Kuete V. (2022). Acute and Sub-Chronic Toxicities Assessment of Methanol Bark Extract of *Hypericum roeperianum* in Rats. S. Afr. J. Bot..

[373] Yang J.-F., Liu Y.-R., Huang C.-C., Ueng Y.-F. (2018). The Time-Dependent Effects of St John’s Wort on Cytochrome P450, Uridine Diphosphate-Glucuronosyltransferase, Glutathione S-Transferase, and NAD(P)H-Quinone Oxidoreductase in Mice. J. Food Drug Anal..

[374] Betti A.H., Stein A.C., Dallegrave E., Wouters A.T., Watanabe T.T., Driemeier D., Buffon A., Rates S.M. (2012). Acute and Repeated-Dose (28 Days) Toxicity Study of *Hypericum polyanthemum* Klotzsch ex Reichardt (Guttiferae) in Mice. Food Chem. Toxicol..

[375] Cantoni L., Rozio M., Mangolini A., Hauri L., Caccia S. (2003). Hyperforin Contributes to the Hepatic CYP3A-Inducing Effect of *Hypericum perforatum* Extract in the Mouse. Toxicol. Sci..

[376] Mai I., Störmer E., Bauer S., Krüger H., Budde K., Roots I. (2003). Impact of St John’s Wort Treatment on the Pharmacokinetics of Tacrolimus and Mycophenolic Acid in Renal Transplant Patients. Nephrol. Dial. Transplant..

[377] Gurley B.J., Swain A., Williams D.K., Barone G., Battu S.K. (2008). Gauging the Clinical Significance of P-Glycoprotein-Mediated Herb–Drug Interactions: Comparative Effects of St. John’s Wort, Echinacea, Clarithromycin, and Rifampin on Digoxin Pharmacokinetics. Mol. Nutr. Food Res..

[378] Jacobson J.M., Feinman L., Liebes L., Ostrow N., Koslowski V., Tobia A., Cabana B.E., Lee D., Spritzler J., Prince A.M. (2001). Pharmacokinetics, Safety, and Antiviral Effects of Hypericin, a Derivative of St. John’s Wort Plant, in Patients with Chronic Hepatitis C Virus Infection. Antimicrob. Agents Chemother..

[379] Brockmöller J., Reum T., Bauer S., Kerb R., Hübner W.-D., Roots I. (1997). Hypericin and Pseudohypericin: Pharmacokinetics and Effects on Photosensitivity in Humans. Pharmacopsychiatry.

[380] Schmitt L.A., Liu Y., Murphy P.A., Petrich J.W., Dixon P.M., Birt D.F. (2006). Reduction in Hypericin-Induced Phototoxicity by *Hypericum perforatum* Extracts and Pure Compounds. J. Photochem. Photobiol. B.

[381] Mühleisen L., Alev M., Unterweger H., Subatzus D., Pöttler M., Friedrich R.P., Alexiou C., Janko C. (2017). Analysis of Hypericin-Mediated Effects and Implications for Targeted Photodynamic Therapy. Int. J. Mol. Sci..

[382] Borrelli F., Izzo A.A. (2009). Herb–Drug Interactions with St John’s Wort (*Hypericum perforatum*): An Update on Clinical Observations. AAPS J..

[383] Lantz M.S., Buchalter E., Giambanco V. (1999). St. John’s Wort and Antidepressant Drug Interactions in the Elderly. J. Geriatr. Psychiatry Neurol..

[384] Nierenberg A.A., Burt T., Matthews J., Weiss A.P. (1999). Mania Associated with St. John’s Wort. Biol. Psychiatry.

[385] Fahmi M., Huang C., Schweitzer I. (2002). A Case of Mania Induced by Hypericum. World J. Biol. Psychiatry.

[386] Ferrara M., Mungai F., Starace F. (2017). St John’s Wort (*Hypericum perforatum*)-Induced Psychosis: A Case Report. J. Med. Case Rep..

[387] Bostock E., Kirkby K., Garry M., Taylor B., Hawrelak J.A. (2018). Mania Associated with Herbal Medicines, Other Than Cannabis: A Systematic Review and Quality Assessment of Case Reports. Front. Psychiatry.

[388] Dalwood J., Dhillon R., Tibrewal P., Gupta N., Bastiampillai T. (2015). St John’s Wort—Is It Safe in Bipolar Disorder?. Aust. N. Z. J. Psychiatry.

[389] Douros A., Bronder E., Andersohn F., Klimpel A., Kreutz R., Garbe E., Bolbrinker J. (2016). Herb-Induced Liver Injury in the Berlin Case-Control Surveillance Study. Int. J. Mol. Sci..

[390] Agollo M.C., Miszputen S.J., Diament J. (2014). *Hypericum perforatum*-Induced Hepatotoxicity with Possible Association with Copaiba (*Copaifera langsdorffii* Desf.): Case Report. Einstein.

[391] Komoroski B.J., Zhang S., Cai H., Hutzler J.M., Frye R., Tracy T.S., Strom S.C., Lehmann T., Ang C.Y., Cui Y.Y. (2004). Induction and Inhibition of Cytochromes P450 by the St. John’s Wort Constituent Hyperforin in Human Hepatocyte Cultures. Drug Metab. Dispos..

[392] Hennessy M., Kelleher D., Spiers J.P., Barry M., Kavanagh P., Back D., Mulcahy F., Feely J. (2002). St John’s Wort Increases Expression of P-Glycoprotein: Implications for Drug Interactions. Br. J. Clin. Pharmacol..

[393] Dürr D., Stieger B., Kullak-Ublick G.A., Rentsch K.M., Steinert H.C., Meier P.J., Fattinger K. (2000). St John’s Wort Induces Intestinal P-Glycoprotein/MDR1 and Intestinal and Hepatic CYP3A4. Clin. Pharmacol. Ther..

[394] Spanakis M., Bakaros E., Papadopoulou S.-N., Fournaraki A., Symvoulakis E.K. (2025). Pharmacoepidemiological Data on Drug–Herb Interactions: Serotonin Syndrome, Arrhythmias and the Emerging Role of Artificial Intelligence. Pharmacoepidemiology.

[395] Hall S.D., Wang Z., Huang S.-M., Hamman M.A., Vasavada N., Adigun A.Q., Hilligoss J.K., Miller M., Gorski J.C. (2003). The Interaction Between St John’s Wort and an Oral Contraceptive. Clin. Pharmacol. Ther..

[396] Murphy P.A., Kern S.E., Stanczyk F.Z., Westhoff C.L. (2005). Interaction of St. John’s Wort with Oral Contraceptives: Effects on the Pharmacokinetics of Norethindrone and Ethinyl Estradiol, Ovarian Activity and Breakthrough Bleeding. Contraception.

[397] Pfrunder A., Schiesser M., Gerber S., Haschke M., Bitzer J., Drewe J. (2003). Interaction of St John’s Wort with Low-Dose Oral Contraceptive Therapy: A Randomized Controlled Trial. Br. J. Clin. Pharmacol..

[398] Schwarz U.I., Büschel B., Kirch W. (2003). Unwanted Pregnancy on Self-Medication with St John’s Wort Despite Hormonal Contraception. Br. J. Clin. Pharmacol..

[399] Berry-Bibee E.N., Kim M.J., Tepper N.K., Riley H.E., Curtis K.M. (2016). Co-Administration of St. John’s Wort and Hormonal Contraceptives: A Systematic Review. Contraception.

[400] Ruschitzka F., Meier P.J., Turina M., Lüscher T.F., Noll G. (2000). Acute Heart Transplant Rejection Due to Saint John’s Wort. Lancet.

[401] Barone G.W., Gurley B.J., Ketel B.L., Lightfoot M.L., Abul-Ezz S.R. (2000). Drug Interaction Between St. John’s Wort and Cyclosporine. Ann. Pharmacother..

[402] Turton-Weeks S.M., Barone G.W., Gurley B.J., Ketel B.L., Lightfoot M.L., Abul-Ezz S.R. (2001). St John’s Wort: A Hidden Risk for Transplant Patients. Prog. Transplant..

[403] Mai I., Bauer S., Perloff E.S., Johne A., Uehleke B., Frank B., Budde K., Roots I. (2004). Hyperforin Content Determines the Magnitude of the St John’s Wort–Cyclosporine Drug Interaction. Clin. Pharmacol. Ther..

[404] Jiang X., Williams K.M., Liauw W.S., Ammit A.J., Roufogalis B.D., Duke C.C., Day R.O., McLachlan A.J. (2004). Effect of St John’s Wort and Ginseng on the Pharmacokinetics and Pharmacodynamics of Warfarin in Healthy Subjects. Br. J. Clin. Pharmacol..

[405] Scholz I., Liakoni E., Hammann F., Grafinger K.E., Duthaler U., Nagler M., Krähenbühl S., Haschke M. (2021). Effects of *Hypericum perforatum* (St John’s Wort) on the Pharmacokinetics and Pharmacodynamics of Rivaroxaban in Humans. Br. J. Clin. Pharmacol..

[406] Martins A.S., Monteiro C., Duarte A.P. (2025). Risks of Oral Anticoagulants: Interactions with Drugs and Medicinal Plants. Sci. Pharm..

[407] Mathijssen R.H., Verweij J., de Bruijn P., Loos W.J., Sparreboom A. (2002). Effects of St. John’s Wort on Irinotecan Metabolism. J. Natl. Cancer Inst..

[408] Frye R.F., Fitzgerald S.M., Lagattuta T.F., Hruska M.W., Egorin M.J. (2004). Effect of St John’s Wort on Imatinib Mesylate Pharmacokinetics. Clin. Pharmacol. Ther..

[409] Goey A.K., Meijerman I., Rosing H., Marchetti S., Mergui-Roelvink M., Keessen M., Burgers J.A., Beijnen J.H., Schellens J.H. (2014). The Effect of St John’s Wort on the Pharmacokinetics of Docetaxel. Clin. Pharmacokinet..

[410] Chan W.J., Adiwidjaja J., McLachlan A.J., Boddy A.V., Harnett J.E. (2023). Interactions Between Natural Products and Cancer Treatments: Underlying Mechanisms and Clinical Importance. Cancer Chemother. Pharmacol..

[411] European Food Safety Authority (EFSA) Scientific Committee (2009). Guidance on Safety Assessment of Botanicals and Botanical Preparations Intended for Use as Ingredients in Food Supplements. EFSA J..

[412] European Food Safety Authority (EFSA) (2012). Compendium of Botanicals Reported to Contain Naturally Occurring Substances of Possible Concern for Human Health When Used in Food and Food Supplements. EFSA J..

[413] Gödtel-Armbrust U., Metzger A., Kroll U., Kelber O., Wojnowski L. (2007). Variability in PXR-Mediated Induction of CYP3A4 by Commercial Preparations and Dry Extracts of St. John’s Wort. Naunyn Schmiedeberg’s Arch. Pharmacol..

[414] Ganzera M., Zhao J., Khan I.A. (2001). Batch-to-Batch Reproducibility of St. John’s Wort Preparations. Pharmacopsychiatry.

[415] U.S. Food and Drug Administration Information for Consumers on Using Dietary Supplements. FDA. https://www.fda.gov/food/dietary-supplements/information-consumers-using-dietary-supplements.

[416] U.S. Food and Drug Administration FDA’s Examples of Drugs That Interact with CYP Enzymes and Transporter Systems. FDA. https://www.fda.gov/drugs/drug-interactions-labeling/healthcare-professionals-fdas-examples-drugs-interact-cyp-enzymes-and-transporter-systems.

[417] U.S. Food and Drug Administration Mixing Medications and Dietary Supplements Can Endanger Your Health. FDA. https://www.fda.gov/consumers/consumer-updates/mixing-medications-and-dietary-supplements-can-endanger-your-health.

[418] European Medicines Agency (EMA) (2022). European Union Herbal Monograph on Hypericum perforatum L., Herba.

[419] European Medicines Agency (EMA) (2023). Assessment Report on Hypericum perforatum L., Herba.

[420] European Medicines Agency (EMA) Herbal Medicinal Products.

[421] European Medicines Agency (EMA) (2022). Guideline on Specifications: Test Procedures and Acceptance Criteria for Herbal Substances, Herbal Preparations and Herbal Medicinal Products/Traditional Herbal Medicinal Products—Revision 3.

[422] Mills E., Montori V.M., Wu P., Gallicano K., Clarke M., Guyatt G. (2004). St John’s Wort (*Hypericum perforatum*): Drug Interactions and Clinical Outcomes. Br. J. Clin. Pharmacol..

[423] Büter B., Orlacchio C., Soldati A., Berger K. (1998). Significance of Genetic and Environmental Aspects in the Field Cultivation of *Hypericum perforatum*. Planta Med..

[424] Çirak C., Radusiene J., Janulis V., Ivanauskas L. (2008). Pseudohypericin and Hyperforin in *Hypericum perforatum* from Northern Turkey: Variation among Populations, Plant Parts and Phenological Stages. J. Integr. Plant Biol..

[425] Carrubba A., Lazzara S., Giovino A., Ruberto G., Napoli E. (2021). Content Variability of Bioactive Secondary Metabolites in *Hypericum perforatum* L.. Phytochem. Lett..

[426] Saffariha M., Jahani A., Jahani R. (2021). A Comparison of Artificial Intelligence Techniques for Predicting Hyperforin Content in *Hypericum perforatum* L. in Different Ecological Habitats. Plant Direct.

[427] Bruni R., Sacchetti G. (2009). Factors Affecting Polyphenol Biosynthesis in Wild and Field-Grown St. John’s Wort (*Hypericum perforatum* L., Hypericaceae/Guttiferae). Molecules.

[428] Lazzara S., Carrubba A., Napoli E. (2021). Cultivating for the Industry: Cropping Experiences with *Hypericum perforatum* L. in a Mediterranean Environment. Agriculture.

[429] Wurglics M., Westerhoff K., Kaunzinger A., Wilke A., Baumeister A., Dressman J., Schubert-Zsilavecz M. (2001). Comparison of German St. John’s Wort Products According to Hyperforin and Total Hypericin Content. J. Am. Pharm. Assoc..

[430] Chandrasekera D.H., Welham K.J., Ashton D., Middleton R., Heinrich M. (2005). Quantitative Analysis of the Major Constituents of St John’s Wort with HPLC-ESI-MS. J. Pharm. Pharmacol..

[431] Booker A., Agapouda A., Frommenwiler D.A., Scotti F., Reich E., Heinrich M. (2018). St John’s Wort (*Hypericum perforatum*) Products—An Assessment of Their Authenticity and Quality. Phytomedicine.

[432] Isacchi B., Bergonzi M.C., Carnevali F., van der Esch S.A., Vincieri F.F., Bilia A.R. (2007). Analysis and Stability of the Constituents of St. John’s Wort Oils Prepared with Different Methods. J. Pharm. Biomed. Anal..

[433] Agapouda A., Booker A., Kiss T., Heinrich M., Csupor D. (2019). Quality Control of *Hypericum perforatum* L.: Analytical Challenges and Recent Progress. J. Pharm. Pharmacol..

[434] Gaid M., Biedermann E., Füller J., Haas P., Behrends S., Krull R., Scholl S., Wittstock U., Müller-Goymann C., Beerhues L. (2018). Biotechnological Production of Hyperforin for Pharmaceutical Formulation. Eur. J. Pharm. Biopharm..

[435] Temerdashev Z., Milevskaya V., Shpigun O., Prasad S., Vinitskaya E., Ryaboko L. (2020). Stability of Some Biologically Active Substances in Extracts and Preparations Based on St. John’s Wort (*Hypericum perforatum* L.) and Sage (*Salvia officinalis* L.). Ind. Crops Prod..

[436] Seyrekoğlu F., Temiz H., Eser F., Yıldırım C. (2024). Optimization of *Hypericum perforatum* Microencapsulation Process by Spray Drying Method. AAPS PharmSciTech.

[437] Hokkanen J., Tolonen A., Mattila S., Turpeinen M. (2011). Metabolism of Hyperforin, the Active Constituent of St. John’s Wort, in Human Liver Microsomes. Eur. J. Pharm. Sci..

[438] Chauvet S., Barras A., Boukherroub R., Bouron A. (2015). Lipid Nanocapsules Containing the Non-Ionic Surfactant Solutol HS15 Inhibit the Transport of Calcium through Hyperforin-Activated Channels in Neuronal Cells. Neuropharmacology.

[439] Hayes C.N., Nakahara H., Ono A., Tsuge M., Oka S. (2024). From Omics to Multi-Omics: A Review of Advantages and Tradeoffs. Genes.

[440] Wang N., Zhang G.C., Li M.M. (2024). Emerging Trends in Systems Biology: Multi-Omics Integration and Beyond. Comput. Mol. Biol..

[441] Meijer D., Beniddir M.A., Coley C.W., Mejri Y.M., Öztürk M., van der Hooft J.J.J., Medema M.H., Skiredj A. (2025). Empowering Natural Product Science with Artificial Intelligence: Leveraging Multimodal Data and Knowledge Graphs. Nat. Prod. Rep..

[442] Cui G., Li M., Guo W., Gao M., Zhu Q., Liao J. (2025). AI Driven Network Pharmacology: Multi-Scale Mechanisms of Traditional Chinese Medicine from Molecular to Patient Analysis. Comput. Struct. Biotechnol. J..

[443] Mohr A.E., Ortega-Santos C.P., Whisner C.M., Klein-Seetharaman J., Jasbi P. (2024). Navigating Challenges and Opportunities in Multi-Omics Integration for Personalized Healthcare. Biomedicines.

[444] Duan X.-P., Qin B.-D., Jiao X.-D., Liu K., Wang Z., Zang Y.-S. (2024). New Clinical Trial Design in Precision Medicine: Discovery, Development and Direction. Signal Transduct. Target. Ther..

[445] Li S., Wang J., Kang Z., Kang X., Chen F., Li W. (2025). Re-Innovation in Clinical Trial Designs Based on Precision Therapy. Cancer Innov..

[446] Fountzilas E., Tsimberidou A.M., Vo H.H., Kurzrock R. (2022). Clinical Trial Design in the Era of Precision Medicine. Genome Med..

[447] Antoniou M., Jorgensen A.L., Kolamunnage-Dona R. (2016). Biomarker-Guided Adaptive Trial Designs in Phase II and Phase III: A Methodological Review. PLoS ONE.

[448] de Toro-Martín J., Arsenault B.J., Després J.-P., Vohl M.-C. (2017). Precision Nutrition: A Review of Personalized Nutritional Approaches for the Prevention and Management of Metabolic Syndrome. Nutrients.

[449] Ramos-Lopez O., Martinez J.A., Milagro F.I. (2022). Holistic Integration of Omics Tools for Precision Nutrition in Health and Disease. Nutrients.

[450] Nourazarain A., Vaziri Y. (2025). Nutrigenomics Meets Multi-Omics: Integrating Genetic, Metabolic, and Microbiome Data for Personalized Nutrition Strategies. Genes Nutr..

[451] Molla G., Bitew M. (2024). Revolutionizing Personalized Medicine: Synergy with Multi-Omics Data Generation, Main Hurdles, and Future Perspectives. Biomedicines.

